# Near-field enhancement of optical second harmonic generation in hybrid gold–lithium niobate nanostructures

**DOI:** 10.1038/s41377-023-01092-8

**Published:** 2023-04-25

**Authors:** Rana Faryad Ali, Jacob A. Busche, Saeid Kamal, David J. Masiello, Byron D. Gates

**Affiliations:** 1 Department of Chemistry and 4D LABS, Simon Fraser University, Burnaby, BC V5A 1S6 Canada; 2 Department of Chemistry, University of Washington, Seattle, WA 98195 USA

**Keywords:** Metamaterials, Nanophotonics and plasmonics, Nonlinear optics, Nanoparticles

## Abstract

Nanophotonics research has focused recently on the ability of nonlinear optical processes to mediate and transform optical signals in a myriad of novel devices, including optical modulators, transducers, color filters, photodetectors, photon sources, and ultrafast optical switches. The inherent weakness of optical nonlinearities at smaller scales has, however, hindered the realization of efficient miniaturized devices, and strategies for enhancing both device efficiencies and synthesis throughput via nanoengineering remain limited. Here, we demonstrate a novel mechanism by which second harmonic generation, a prototypical nonlinear optical phenomenon, from individual lithium niobate particles can be significantly enhanced through nonradiative coupling to the localized surface plasmon resonances of embedded gold nanoparticles. A joint experimental and theoretical investigation of single mesoporous lithium niobate particles coated with a dispersed layer of ~10 nm diameter gold nanoparticles shows that a ~32-fold enhancement of second harmonic generation can be achieved without introducing finely tailored radiative nanoantennas to mediate photon transfer to or from the nonlinear material. This work highlights the limitations of current strategies for enhancing nonlinear optical phenomena and proposes a route through which a new class of subwavelength nonlinear optical platforms can be designed to maximize nonlinear efficiencies through near-field energy exchange.

## Abstract (web summary)

Upconversion of light from its fundamental wavelength (FW) to its second harmonic (SH) is enhanced 32× in micron-scale lithium niobate (LiNbO_3_) spheres through near-field interactions with gold (Au) nanoparticles.

## Graphical abstract

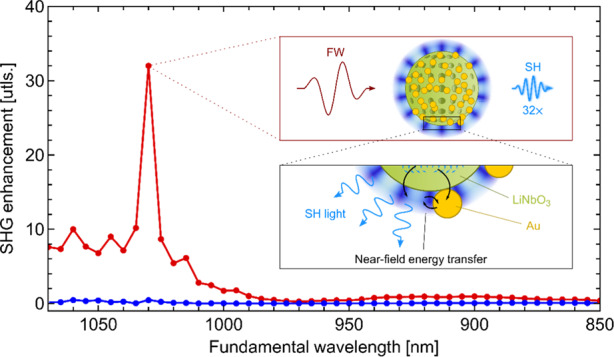

## Introduction

Ultrafast optical frequency conversion using nonlinear optical (NLO) harmonic generation in micro- and nanoscale materials is emerging as an important phenomenon in the basic and applied study of photonics^1^, as well as for the development of novel sensing and imaging techniques in materials science, chemistry, and biology^2^. Perhaps the simplest of the ultrafast NLO processes relevant to each of these fields is second harmonic generation (SHG), in which two input fields oscillating at a fundamental frequency are coherently combined into an output field oscillating at twice the fundamental frequency^3,4^. In general, SHG is prized for its narrow-band operation, relatively high conversion efficiency among NLO processes, and natural occurrence in a wide array of inorganic materials^3,4^.

However, even in “good” NLO materials, the nonlinear wave motion underpinning SHG is generally either weak^5^ or slower than the ~1 ps timescales desired in ultrafast applications^6^. To overcome this difficulty, SHG photonics designs have until recently centered around the use of relatively large NLO crystals within which the fundamental wavelength (FW) and SH light waves can be constructively interfered to overcome intrinsically low SHG efficiencies^7,8^. It has become clear, however, that much smaller micro- and nanoscopic SHG devices are more desirable for cutting-edge applications like few-molecule sensing^9^, bioimaging^10^, and phase-resolved light emission^11^ due to the unique degree of control over the spectrum and spatial distribution of the scattered fields that miniaturized optical systems provide^12^.

Unfortunately, due to the complicated field profiles of nanostructured particles, interference and phase-matching techniques are rendered ineffective at the nanoscale. As a result, the absolute efficiency of an ultrafast NLO process within a nanostructure tends to depend on the microscopic volume of NLO material used^13^. Researchers continue to pursue fundamentally new NLO enhancement techniques and novel nanofabrication solutions to optimize or supersede this limit, exploring the NLO properties of light confined within resonant dielectric^14^, metallic^15^, and hybrid^16^ micro- and nanostructures, as well as within nanopatterned waveguides^17^.

Our investigation addresses both the scientific and engineering challenges posed in NLO nanophotonics by revealing a new and practical strategy for enhancing the SHG response. Specifically, we use the surface-localized near-fields of disordered arrays of gold (Au) nanoparticles (NPs) to create hitherto unexplored energy-transfer pathways for nanolocalized upconverted light. These disordered nanostructures, comprised of ~1 µm diameter mesoporous lithium niobate (LiNbO_3_) microspheres coated in a dispersed layer of 10 nm diameter Au NPs, allow for a straightforward and high-throughput synthetic process devoid of the difficulties of precision nanofabrication while simultaneously producing an NLO system with a large number of regions of enhanced field intensity (so-called “hot spots”) between the LiNbO_3_ and Au surfaces.

In stark contrast, many past investigations^18–23^ have achieved enhancement factors between 5 and 20 by employing carefully tailored hybrid nanostructures. The manufacture of such structures is technically challenging, causing difficulties with the reproduction of large enhancement ratios between samples^22^. Other studies have demonstrated enhancement factors of ~100 to 1000 more reliably using simple core-shell SHG systems^16,24^. However, the field profiles and resonance structures of shells, which are often assembled as collections of large NPs, can be complex and difficult to separate from the influence of imperfections^25^, and can be susceptible to degradation under exposure to high-intensity lasers^26^. As such, we have chosen to use a more rarefied ensemble of smaller and more easily modeled NPs to isolate the NLO enhancing abilities and governing physical principles of an optically robust and reproducible proof-of-concept nanostructure design.

As will be shown below, our multiple-hot-spot geometry enhances SHG by a factor of 32. Further, this enhancement is clearly attributed through a complete make-measure-model synthesis and characterization approach to the near-field localization and resonant behaviors of metallic nanoantennas that are generally considered unsuitable for the task of enhancing far-field (i.e., radiative) emission. Although a few very recent studies^6,27^ have measured moderate (e.g., ~7×) SHG enhancements using similarly disordered NP ensembles, each was either unable to isolate single nanostructures^27^, or to thoroughly explore the governing mechanism behind the observed SHG enhancements^6^. Our results, therefore, represent both the first demonstration of NLO enhancement values of >10 from single disordered nanostructures, as well as the discovery of as-yet unknown mechanisms by which strongly-subwavelength NPs can enhance SHG.

## Results

### Preparation of hybrid SHG nanostructures

**Fig. 1:**
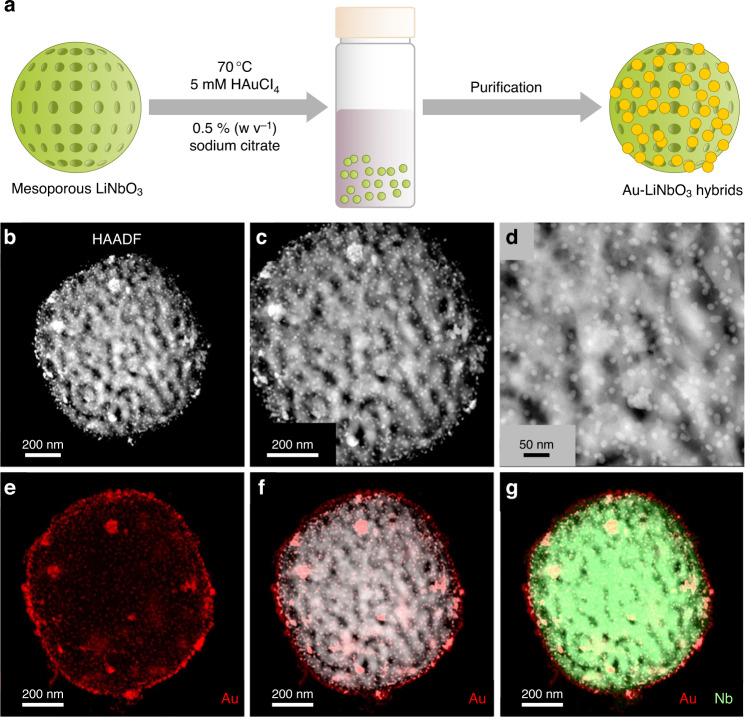
Synthesis of hybrid Au–LiNbO_3_ nanostructures. **a** Representative schematic to prepare the hybrids of Au NPs with LiNbO_3_ as achieved using an in situ synthesis method. Assemblies of Au–LiNbO_3_ hybrid particles as characterized by scanning transmission electron microscopy (STEM) operating in: **b**, **c**, **d** a high-angle annular dark-field (HAADF) mode. Energy dispersive X-ray spectroscopy (EDS) analysis of the Au–LiNbO_3_ hybrids obtained by STEM techniques was used to create EDS maps of: **e** Au NPs; **f** Au NPs overlaid on the HAADF image of the assemblies; and **g** overlaid Au-Nb signals within these assemblies

Selecting an NLO material with a large NLO susceptibility coefficient is critical to improve the efficiency of the SHG conversion process at smaller scales. Relative to many NLO materials, LiNbO_3_ is a unique photonic material, often referred to as the “silicon of photonics” due to its relatively large second-order susceptibilities (e.g., 41.7 pm/V), higher optical damage resistance (e.g., 20 W/cm^2^ for congruent LiNbO_3_ crystals), and a relatively wide window of optical transparency (e.g., 400–5000 nm)^3,28^. In the first step, monodisperse mesoporous LiNbO_3_ particles were prepared by modifying a previously reported method (details in Materials and Methods Section S.1)^4^. Scanning transmission electron microscopy (STEM) images confirm that diameters of the LiNbO_3_ particles were ~1000 nm (Figs. 1b–d and S1) and that each particle has a mesoporous structure. These mesoporous particles contained a randomly disordered network of distinct grains in its bulk and a textured surface that contains randomly distributed ~20 nm diameter pores. In addition, X-ray diffraction (XRD) patterns of the LiNbO_3_ particles in a powder form were acquired and correlate well with a reported LiNbO_3_ reference (Fig. S2), displaying a distinct rhombohedral phase (space group *R3c*) that accompanies strong SHG behavior^3,28^. Further evidence of the rhombohedral phase of the LiNbO_3_ particles was provided by Raman spectroscopy of a powdered sample (Fig. S3), in which the observed bands are characteristic of rhombohedral LiNbO_3_ and agree well with a commercially available LiNbO_3_ standard^28^.

In the second step to prepare the hybrid Au–LiNbO_3_ particles, we utilized an in situ seed-mediated growth to load the surfaces of the mesoporous LiNbO_3_ with Au NPs (Fig. 1a). This approach used a one-pot synthetic technique that eliminated the need for precision nanoengineering techniques to position the plasmonic materials upon their NLO support^29^. Briefly, an aqueous suspension of bare, mesoporous LiNbO_3_ particles in the presence of Au chloride as a precursor was heated at 70 °C for 3 h. The in situ reduction of the Au precursor using sodium citrate as a reducing agent led to the formation of the hybrid structures, wherein the Au NPs were observed both through electron microscopy imaging (Fig. 1b–d) and energy dispersive X-ray spectroscopy (EDS) based mapping techniques (Figs. 1e–g and S4) to be uniformly distributed over the surfaces of the LiNbO_3_ particles. Moreover, the Au particles were limited to diameters of ~10 nm (Figs. 1b–d and S5). These Au nanoparticles were deposited on the outer surfaces, as well as within the pores of the mesoporous LiNbO_3_. Synthesis of hybrid particles prepared from porous LiNbO_3_ supports revealed that the number of Au NPs loaded onto each LiNbO_3_ particle was ~900 to 1000.

### Linear and nonlinear optical spectroscopy

**Fig. 2:**
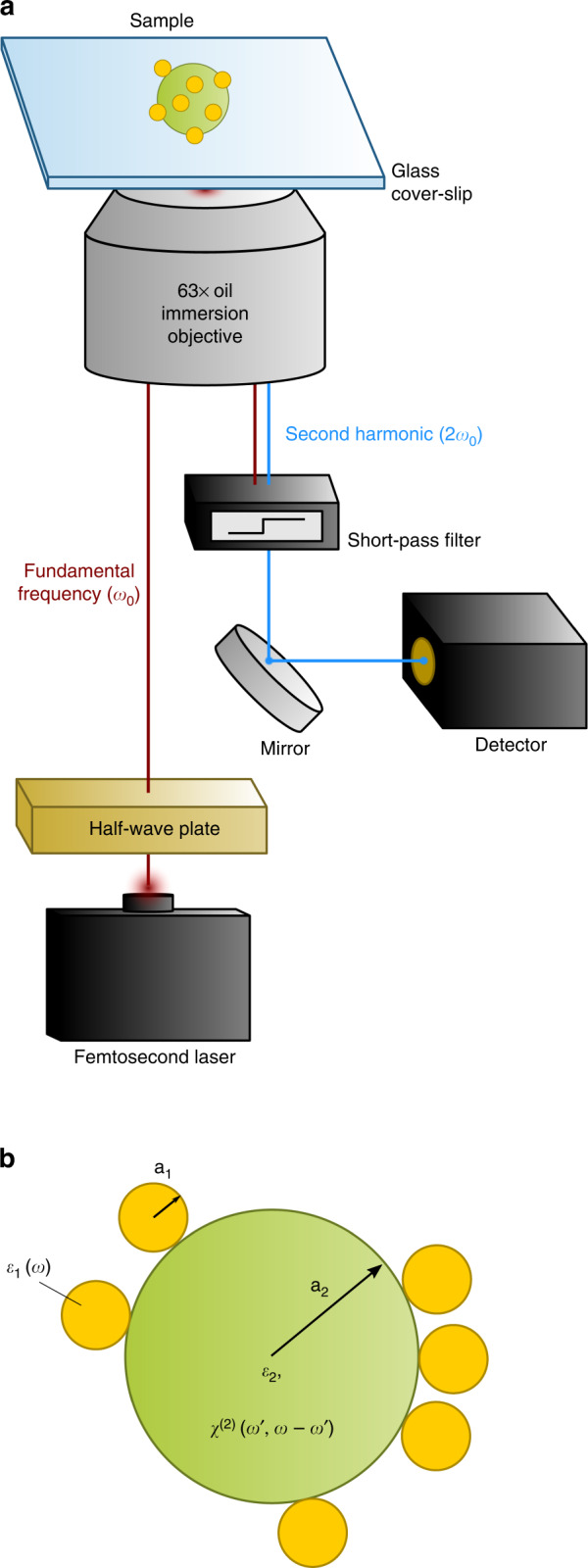
Experimental geometry. **a** Schematic of the microscope configuration used to characterize the second harmonic generation (SHG) response of single particles. This microscope was operated in a reflection mode setup and was equipped with a femtosecond laser, half-wave plate, and objective lenses of different magnifications including a 63× oil immersion lens. **b** Sketch of the sample geometry of the hybrid Au–LiNbO_3_ particles

Analysis of the SHG emission from the hybrid nanostructures was performed using a Leica SP5 commercial two-photon microscope. The incident light was generated using a pulsed, mode-locked Ti:sapphire laser with a pulse width of ~140 fs. The resonances of the NLO particles have periods between 0.1 and 0.3 fs such that their driven oscillatory behavior and associated SHG scattering are independent of the pulse envelope. Additionally, with a repetition rate of 80 MHz, the spacing between pulses (12.5 ns) is much longer than the lifetimes of the target resonances (2–30 fs) such that scattering data from each pulse is considered to be uncorrelated to the data obtained from prior pulses. Moreover, the use of short, well-separated pulses also enabled the laser to deliver the high-intensity field required to generate the observable SHG signal. The scattered light and SHG emission from single particles were each analyzed by collecting the light with a microscope objective lens and analyzing the signal using a spectrometer that enabled separation of the input signal (e.g., fundamental wavelength) from the output signal (e.g., SH wavelength). Finally, the laser frequency was tuned from 680 nm to 1080 nm to analyze the wide spectral response of these NLO materials. Additional details of the experimental setup are shown in Fig. 2.

**Fig. 3:**
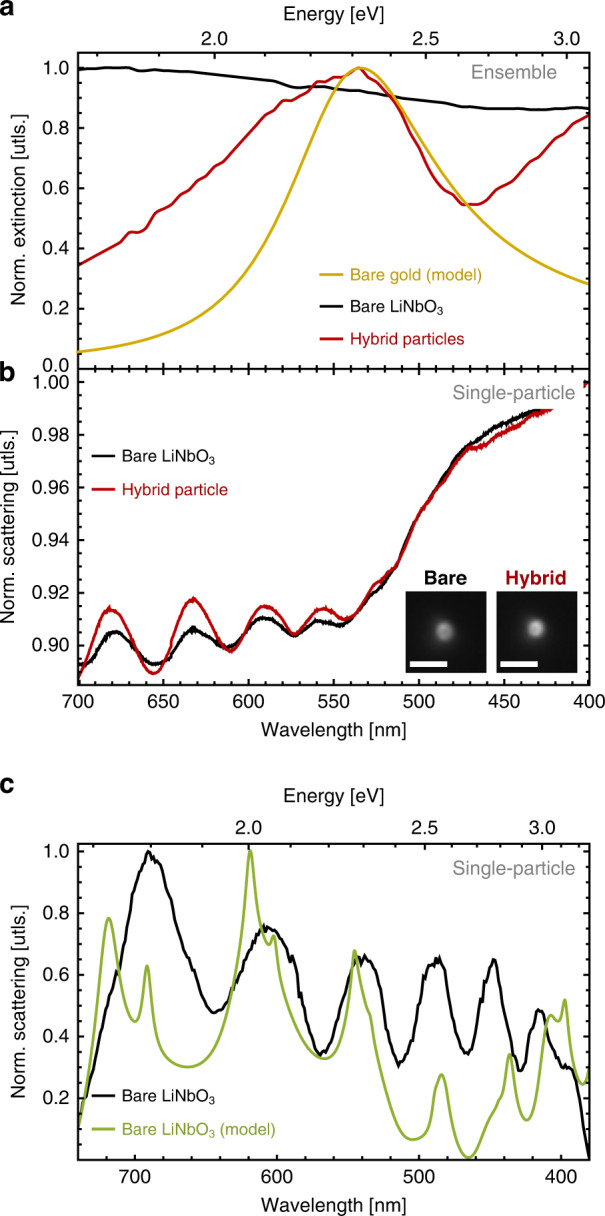
Experimental and theoretical optical spectra. **a** Measured extinction spectra for ensembles of bare LiNbO_3_ particles (black) and of hybrid Au–LiNbO_3_ particles (red) compared to the modeled dipole plasmon extinction (gold). These spectra show a plasmonic peak at ~530 nm for the hybrid Au–LiNbO_3_ particles, while the bare LiNbO_3_ particles were optically transparent. **b** Light scattering spectra for individual bare LiNbO_3_ particles and individual Au–LiNbO_3_ hybrid particles measured in a dark field (DF) configuration with white light illumination. The insets in (**b**) portray gray color images (scale bars = 2 μm) for (black) a single LiNbO_3_ particle and (red) a single Au–LiNbO_3_ hybrid particle supported on glass coverslips. The data were normalized to the maximum value of the scattering intensity. **c** Theoretical scattering cross sections (green) from a model microsphere with dielectric constant 6.3 + 0.05*i* and radius 700 nm in comparison with experimentally measured scattering data from a single, bare LiNbO_3_ particle (black), normalized to range from 0 to 1 within the selected frequency window

First, we acquired and compared the extinction spectra of the hybrid Au–LiNbO_3_ particles, pristine LiNbO_3_ particles, and Au NPs. Aqueous suspensions of each sample were prepared and characterized using a UV-visible spectrometer. No extinction peak was observed for the pristine LiNbO_3_ particles indicating their optical transparency in the region from 400 to 800 nm (Fig. 3a). We prepared ~10 nm diameter Au NPs and acquired and characterized their extinction spectra to obtain a detailed comparison to the hybrid Au–LiNbO_3_ particles. This suspension of Au NPs had an extinction peak at ~520 nm due to their plasmonic response (Fig. S6). The hybrid particles of Au–LiNbO_3_, however, exhibited a broad extinction band from the visible to the near-IR with a maximum centered at 530 nm (Fig. 3a). The presence of this peak in the hybrid Au–LiNbO_3_ particles was due to the contributions of the Au NPs. The broadness and red shift in the resonance peak of the Au–LiNbO_3_ particles could be attributed to the formation of Au NP aggregates on the surfaces of the LiNbO_3_ particles^30^.

For the linear scattering and nonlinear optical analyses of individual particles, we prepared separate, dilute aqueous suspensions (0.1 mg/mL) of both the LiNbO_3_ and Au–LiNbO_3_ particles via sonication. The resultant suspensions were drop cast onto glass coverslips and dried under a vacuum to evaporate the solvent. Microscopy analyses of the resulting substrates indicated the presence of well-dispersed, individual particles. The linear optical spectra of individual Au–LiNbO_3_ and pristine LiNbO_3_ particles were obtained to evaluate their scattering profiles (Fig. 3b). A dark-field optical spectroscopy setup was used for these scattering measurements where individual particles were imaged using a 50× dark-field objective. The individual particles were illuminated by white light generated from a halogen lamp. The scattered light was collected by the same objective lens and detected with an imaging spectrometer through a pinhole that restricted the collection of signal from the area around the measured nanoparticle. We determined the position and the number of the scattering peaks in the linear optical spectra of each type of particle. Four distinct scattering peaks were observed for both the Au–LiNbO_3_ hybrids and pristine LiNbO_3_ particles. As the diameters of the particles were comparable to the wavelength of the incident light, the linear scattering response of these materials does not resemble a Rayleigh scattering profile. Due to a negligible change in the size of Au–LiNbO_3_ hybrids relative to the pristine LiNbO_3_, the electromagnetic field inside each type of particle was consistent, resulting in indistinguishable Mie scattered modes between pristine and hybrid particles^31^. These scattering results are comparable to the Mie scattering spectra previously reported for LiNbO_3_ based materials having comparable sizes^32^. The presence of distinct resonances in the scattering spectra indicate that both the Au–LiNbO_3_ and LiNbO_3_ particles act as optical Mie resonators. By considering these optical measurements of individual Au–LiNbO_3_ and LiNbO_3_ particles, it can be concluded that the Au NPs play no significant role in the Mie resonances in these materials. The features of Mie scattering were not measurable below 400 nm and above 700 nm due to the loss of sensitivity of the measurement setup in these regions. The linear optical spectra of individual Au NPs with diameters of ~10 nm could not be detected due to their smaller size and limitations of the objective lens to locate individual Au particles at the nanoscale. A further improvement of the dark-field spectroscopy setup in the UV range is challenging because of the lack of objective lenses that have both a high transmission in the UV range and no chromatic aberrations across both the UV and visible range.

**Fig. 4:**
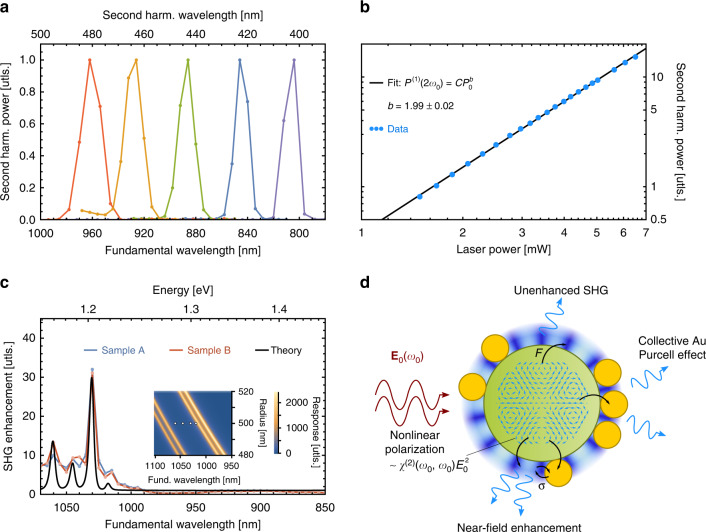
Second harmonic generation enhancement. **a** SHG spectra of the Au–LiNbO_3_ hybrid particles as the FW is tuned from 800 nm (violet) to 960 nm (red). **b** Power dependence of SHG at 800 nm. The power scattered at the SH (blue dots) is proportional to the input laser power at the FW to the power *b* = 1.99 (95% confidence interval of ±0.02, see fit [black line]) times a normalization constant *C*. **c** Enhancement of the scattered SHG power as a function of the fundamental wavelength for an individual hybrid Au–LiNbO_3_ nanostructure recorded by sweeping the excitation laser wavelength from 850 to 1070 nm with a step size of 5 nm. The experimental results (blue, red) are overlaid by the modeled near-field coupling enhancements (black). The plotted curves are shifted phenomenologically by 10–25 nm from the solutions of Eq. (1) to align with the experiment, as is highlighted in the “Discussion”. Inset: response functions (SI, Section S.2.4.2) of the narrow Mie resonances of a bare LiNbO_3_ sphere as a function of the sphere radius and photon energy. The response function maxima (gold/white) lie at the same resonance energies *ω*_***β***_ as the SHG enhancement peaks, implying a red (blue) shift in the narrow SHG enhancement features with increasing (decreasing) *a*_2_. The white dots indicate the shifted peak positions shown in the main panel. Further details are available in the SI. **d** A diagram of the pathways for energy transfer of light within the system. Clockwise from left: incident light (dark red), SHG (blue arrows) through energy transfer from nonlinear polarization (blue vector field) to Mie resonances (halo), superradiant Purcell-enhanced SHG, and near-field enhanced SHG

We acquired input wavelength-dependent (Fig. 4a) and power-dependent (Fig. 4b) NLO spectra for the prepared mesoporous Au–LiNbO_3_ hybrids to ensure the materials are SHG active and to verify the second-order nature of the detected signals. The power-dependent SHG signals were collected by varying the power of the laser at the FW of 800 nm while imaging individual Au–LiNbO_3_ particles on the substrate. The image emission matrix was averaged to yield a mean value in regions of interest selected to exclude the majority of the dark pixels. The selected regions of interest were maintained unchanged throughout the series of acquired images. The logarithms of the mean values for the SHG signals were plotted against the logarithm of the laser power. The slopes of the linear fits reflect the power dependence of the SHG on the pumping intensity. A slope of ~2.0 for the Au–LiNbO_3_ particles confirm that the signal is generated from a second-order NLO process (i.e., SHG). The SHG for individual Au–LiNbO_3_ particles was also assessed for a series of discrete fundamental wavelengths. The resultant SH responses were each normalized by dividing their intensities by the maximum scattering intensity within each SH band when assessing the frequency doubling behavior of the SHG process. A tunable SH response was observed at 400, 420, 440, 460 and 480 nm when the product was excited with FWs of 800, 840, 880, 920, and 960 nm, respectively (Fig. 4a). These results correlated well to the anticipated frequency doubling of the fundamentals.

The SHG spectra were acquired for a cluster of Au NPs using the same experimental setup to verify whether the strong SHG emission originated from the surface of the gold rather than from the NLO LiNbO_3_ core. We used a cluster of solid Au NPs since individual 10 nm diameter gold NPs were difficult to drop cast and locate using the 63× objective lens. We scanned the FWs over the range from 850 nm to 1070 nm and collected the SHG response for the clusters of Au NPs (Fig. S7). No SHG response was generated from 425 to 535 nm for the Au NPs. The SHG emitted by the Au–LiNbO_3_ hybrids is, therefore, exclusively generated by the LiNbO_3_ particles. For NPs made out of centrosymmetric materials such as gold, SHG is mainly emitted from an Angstrom-thin layer near the particle surface since SHG emission is mainly induced by the surface normal component of the second-order nonlinear susceptibility tensor^33,34^. The Au NPs used in this work are, however, spherical and the SHG generated from the bulk of the gold nanoparticle and through the surface-tangential component of the second-order nonlinear susceptibility is expected to be very low and are ignored in this study (Fig. S7)^34^.

We measured the SHG spectra of individual pristine LiNbO_3_ and hybrid Au–LiNbO_3_ particles by sweeping the incident laser over the wavelength range from 850 to 1070 nm in steps of 5 nm to investigate whether Au NPs play a role in the SHG enhancement of the hybrid structures (Figs. 4c and S14a). The corresponding SHG response was recorded from 425 to 535 nm. A comparison was made between the SHG intensity from individual Au–LiNbO_3_ particles with individual pristine LiNbO_3_ particles examined under the same experimental parameters to quantitatively determine the NLO enhancement. This method to calculate the SHG enhancement has been used in the reported literature, such as where the enhancement of the NLO signals from metasurfaces is calculated by comparing the patterned metasurface to the unpatterned original surface^35,36^. After normalization of the SHG output of the Au–LiNbO_3_ hybrids to the SHG output of the bare LiNbO_3_, we were able to calculate the enhancement factors. The obtained enhancement factor was plotted against the FWs of the measurement. The highest enhancement value was obtained at a FW of 1030 nm, reaching an enhancement of 32 times (Figs. 4c and S14a). This evaluation demonstrates a corresponding enhancement in the SHG response at 515 nm by a factor of 32 for the mesoporous LiNbO_3_ particles loaded with Au NPs. We prepared Au–LiNbO_3_ hybrids with a lower loading of Au NPs to understand what role the Au NPs play in enhancing the SHG response (Figs. S8 and S9). The average number of Au NPs on each LiNbO_3_ particle was ~6. We acquired the SHG response of these Au–LiNbO_3_ hybrids with their lower loading of Au NPs and compared this response to the NLO response of the bare LiNbO_3_ particles. Interestingly, no enhancement in the SHG response was observed for these hybrids of Au–LiNbO_3_ with only a few Au NPs (Fig. S10). These observations indicated the need to have a higher loading of Au NPs on the surfaces of the LiNbO_3_ to enhance the SHG response.

### Interpretation of scattering signals

With plasmon-induced enhancements greater than 30, the hybrid particles in this investigation show commensurate performance with other NLO nanostructures of a similar size without the requirements of being prepared using precision nanoengineering techniques. For example, SHG emission enhancement factors of ≤30 and ~22 have been previously reported^37,38^ in nanostructures that employ carefully tailored plasmonic nanoantennas. In contrast, the positions of the plasmonic Au NPs used in this study are not precisely tuned to efficiently direct the optical signals of interest, obscuring the phenomena underpinning their ability to magnify SHG.

More precisely, hybrid SHG nanostructures generally employ metal NPs to perform one of two functions relevant to upconversion. The first function, in which plasmons enhance the in-coupling of light to the SHG material, employs the near-fields of highly polarizable plasmons that are tuned to the FW to augment the pump field of an NLO process^16,37^. In the second function, radiative plasmons tuned to the up- or down-converted frequency enhance the out-coupling of light by extracting energy from the NLO material through near-field interactions and then quickly scattering it to the far-field, i.e., via the Purcell effect^19,38^. Carefully designed nanostructures can make use of both strategies^22^, and antenna effects can be significant whether or not the NLO material itself is optically resonant^21^.

Our nanoantennas are far too small to act as radiative out-coupling nanoantennas yet have a plasmon resonance that is aligned with the SH output maximum. The most straightforward explanations for the surprising efficacy of these “bad antenna” NPs are that the NPs’ enhanced SHG emission is a result of either of the following hypotheses: (i) coupling effects, in which optical energy is routed through the system via more complicated interactions than can be captured by a simple antenna picture; or (ii) superradiant plasmon scattering generated by their collective interaction with the radiation field.

No existing model can fully address either of these questions. Recent theoretical studies have investigated several related phenomena, including radiation from dipole-driven Mie scatterers^39^, NLO scattering from bare dielectric spheres^40^, NLO emission from quantum emitters in idealized Fabry-Pèrot cavities^41,42^, and light emission from ensembles of oscillating dipoles^43,44^. However, a novel synthesis of the ideas from each study is required to uncover the operative SHG enhancement mechanism(s) in our nanostructures.

Beginning with hypothesis (i), we first simplify the particle geometries highlighted in Fig. 1 in a manner consistent with prior investigations of complicated multicrystalline scatterers^13^. In detail, we model each microsphere as a smooth, isotropic spherical particle of radius *a*_2_ = 500 nm. We also simplify the form of the incoming light, assuming the laser field ${{{\mathbf{E}}}}_0\left( {{{{\mathbf{r}}}},\omega } \right)$ to be a monochromatic plane wave of characteristic field strength *E*_0_ and frequency ω_0_ that travels in the *z*-direction and is linearly polarized along *x*. That is to say, ${{{\mathbf{E}}}}_0\left( {{{{\mathbf{r}}}},\omega } \right) = E_0\left[ {\pi \delta \left( {\omega - \omega _0} \right)\exp \left( {i\omega _0z/c} \right) + \pi \delta \left( {\omega + \omega _0} \right)\exp \left( { - i\omega _0z/c} \right)} \right]{{{\hat{\mathbf x}}}}$.

The combination of LiNbO_3_’s nearly constant^45^ dielectric function $\epsilon _2 = 5.5 + 0.035i$ (see Supplementary Information [SI], Section S.2.1) and relatively weak second-order nonlinear susceptibility $\left| {\boldsymbol{\chi}}^{\left( 2 \right)}\left( {\omega _0,\omega - \omega _0} \right) \right| \approx 2 \times 10^{ - 6}{{{\mathrm{cm}}}}/{{{\mathrm{statV}}}} \approx 60\,{{{\mathrm{pm}}}}/{{{\mathrm{V}}}}$ (SI, Section S.2.1 and Fig. S11) for observation frequencies ω and laser frequencies in the optical region of interest allows for simplification of the LiNbO_3_’s NLO behaviors. Specifically, the nonlinear wave motion of an excited microsphere can be perturbatively expanded as the superposition of Mie resonances at the laser FW, SH, third harmonic, and so on (SI, Section S.2.4).

The FW scattered electric field **E**_sca_(**r**, ω) is simply the component of the total electric field for which the output frequency matches the laser frequency. The FW resonances in general have both appreciable magnetic and electric fields, have nondipolar symmetry, and produce a field ${{{\mathbf{E}}}}_{{{{\mathrm{sca}}}}}\left( {{{{\mathbf{r}}}},\omega } \right) = \mathop {\sum}\nolimits_{\boldsymbol{\alpha}} {a_2^{ - \ell - 2}} \left[ {\rho _{\boldsymbol{\alpha}} \left( \omega \right){{{\mathbf{X}}}}_{\boldsymbol{\alpha}} \left( {{{{\mathbf{r}}}},k} \right) + \rho_{\boldsymbol{\alpha}}^{\ast}\left( { - \omega } \right){{{\mathbf{X}}}}_{\boldsymbol{\alpha}}^ \ast \left( {{{{\mathbf{r}}}},k} \right)} \right]$ outside the microsphere’s surface. Here, *k* = *ω*/*c* is the wavenumber and ***α*** = $\left\{ {T,p,\ell ,m} \right\}$ is the collective index of each Mie mode near the FW, with *T* = *E*, *M* signifying the type (electric or magnetic) of the mode, *p* (even or odd) its *x*-axis reflection symmetry, $\ell = 1,2,\, \ldots$ its spherical harmonic order, and $m = 0, \ldots ,\ell$ its spherical harmonic degree. The vector spherical harmonic mode functions **X**_***α***_(**r**, *k*) and multipole moments *ρ*_***α***_(ω) encode the spatial lobe structure and spectrum, respectively, of each resonance.

From here, in accordance with previous work^40^, we assume that upconversion in the LiNbO_3_ spheres during the experiment is coherent and occurs when a small portion of the energy bound in the laser and FW scattered fields is converted into a second material polarization oscillating at the SH (2*ω*_0_). This so-called nonlinear polarization field is always proportional to $| {\boldsymbol{\chi}}_2^{( 2 )}( {\omega _0,\omega _0} ) |E_0^2$ (see Eq. [S38]) and generates two SH electric fields of its own.

The first SH field, ${\boldsymbol{\cal{E}}}_0\left( {{{{\mathbf{r}}}},\omega } \right)$, describes direct radiation by the nonlinear polarization. It does not interact with the LiNbO_3_ sphere directly. The second, ${\boldsymbol{\cal{E}}}_{{{{\mathrm{sca}}}}}( {{{{\mathbf{r}}}},\omega } ) = \mathop{\sum}\nolimits_{\boldsymbol{\beta}} {a_2^{ - \ell - 2}} [ {\rho _{\boldsymbol{\beta}} ( \omega ){{{\mathbf{X}}}}_{\boldsymbol{\beta}} ( {{{{\mathbf{r}}}},k} ) + \rho_{\boldsymbol{\beta}}^{\ast}( { - \omega } ){{{\mathbf{X}}}}_{\boldsymbol{\beta}}^{\ast} ( {{{{\mathbf{r}}}},k} )} ]$, is a scattered field that interacts with the LiNbO_3_ sphere similarly to the scattered FW electric field ${{{\mathbf{E}}}}_{{{{\mathrm{sca}}}}}\left( {{{{\mathbf{r}}}},\omega } \right)$ but is driven by the nonlinear polarization rather than directly by the laser light. In fact, ${\boldsymbol{\cal{E}}}_{{{{\mathrm{sca}}}}}\left( {{{{\mathbf{r}}}},\omega } \right)$ can be defined identically to its FW counterpart but with the sum over ***α***, i.e. the sum over the set of Mie resonances near the FW, replaced by a sum over the set of Mie resonances ***β*** = $\left\{ {T^\prime ,p^\prime ,\ell ^\prime ,m^\prime } \right\}$ near the SH. The SH scattered fields can also interact with the NP ensemble which, in this case, acts as a set of symmetry-breaking resonant cavities that allow mixing between the otherwise orthogonal SH Mie resonances of the LiNbO_3_ sphere.

Whether excited by ${\boldsymbol{\cal{E}}}_0$ or ${\boldsymbol{\cal{E}}}_{sca}$, the excited NPs can transfer energy back to the LiNbO_3_ sphere through their near-fields. Thus, the two SH field-NP interactions provide additional pathways for energy to be transferred from the abundant incident power^31^ $P_0 = ca_2^3E_0^2/8$ of the laser to the LiNbO_3_ microsphere, which then scatters more SH light as a result. Figure 4d provides a sketch of these processes.

Quantification of the resulting SHG enhancements is dramatically simplified by the fact that each Mie resonance ***β*** is well-approximated as a discrete oscillator with resonance frequency *ω*_***β***_ and damping rate *γ*_***β***_ (see SI, Eq. [S16] and Section S.2.2). The plasmons in each NP, here assumed to have radii *a*_1_ = 5 nm, have similarly simple oscillator properties (SI, Section S.2.2), with resonance frequencies *ω*_1_ and damping rates *γ*_1_.

However, even with these conveniences, explicitly describing the numerous energy-transfer pathways between the *N* NPs of the ensemble and the LiNbO_3_ is difficult. Therefore, we first model the near-field energy exchange between the LiNbO_3_ and Au by considering a reduced system with only a single Au NP coupled to the LiNbO_3_ modes. Letting the NP dipole be oriented along the *ν* Cartesian axis with a moment *d*_*ν*_(*ω*), its motion along with the motion of each SH Mie moment *ρ*_***β***_(*ω*) can be quantified from coupled equations of motion (SI, Section S.2.4), here given in the Fourier domain:

1 $$\begin{array}{l}d_{\nu} (\omega)\left({\Omega}_{1}^{2} - \omega^{2} - i\omega\gamma_1 \right)\\ = \frac{f_{\nu} a_{1}^{3}}{2\omega_{1}e}\eta_{1}(\omega)F_{1\nu}\left({\mathbf{r}}_{0},\omega \right)\\ +\, \frac{f_{\nu} a_1^{3}}{2{\omega_1}e^{2}}{\eta_1}(\omega)\sum\limits_{{\boldsymbol{\beta}}} \frac{1}{a_{2}^{\ell - 1}} \left[ \sigma _{{\boldsymbol{\beta}}\nu}\left({\mathbf{r}}_{0} \right)\rho_{{\boldsymbol{\beta}}} (\omega) + \sigma_{{\boldsymbol{\beta}} \nu}^{\ast} \left({\mathbf{r}}_{0} \right)\rho _{{\boldsymbol{\beta}}}^{\ast} \left(-\omega \right) \right]\\ \rho _{{\boldsymbol{\beta}}} (\omega)\left({\Omega}_{{\boldsymbol{\beta}}}^{2} - \omega^{2} - i\omega \gamma_{{\boldsymbol{\beta}}} \right) \\= \frac{f_{{\boldsymbol{\beta}}}a_2^{\ell + 2}}{2\omega_{{\boldsymbol{\beta}}}e}\eta_{{\boldsymbol{\beta}}} (\omega)F_{2\boldsymbol{\beta}}(\omega)\\ +\, \frac{f_{{\boldsymbol{\beta}}}a_2^{\ell + 2}}{2\omega_{{\boldsymbol{\beta}}}e^{2}}{\eta_{{\boldsymbol{\beta}}}} (\omega)\sum \limits_{\nu} \sigma _{{\boldsymbol{\beta}} \nu} \left( {\mathbf{r}}_{0} \right)d_{\nu} (\omega)\end{array}$$

In addition to the previously defined constants, each of the moments has a natural frequency ${\Omega}_{1,{\boldsymbol{\beta}}} = \sqrt {\omega _{1,{\boldsymbol{\beta}} }^2 + \gamma _{1,{\boldsymbol{\beta}} }^2/4}$, an oscillator strength *f*_*ν*,***β***_, and a charge *e*. On the right-hand side of Eq. (1), the forces $F_{1\nu }({{{\mathbf{r}}}}_0,\omega )$ and $F_{2{\boldsymbol{\beta}}}(\omega )$ describe the driving of the Au NP dipoles and Mie modes, respectively, by the ${\boldsymbol{\chi}}_2^{\left( 2 \right)}$-upconverted incident light with the NP centered at **r**_0_. The analytical form of these forces is complicated and is left for the SI (see Section S.2.4). The phases of the moments’ responses to these forces are encoded by their characteristic phase delays $\eta _1\left( \omega \right) = 2\omega _1\cos \psi _1 + \gamma _1\sin \psi _1 + 2i\omega \sin \psi _1$ and $\eta_{\boldsymbol{\beta}} ( \omega ) = ( {{\Omega}_{\boldsymbol{\beta}}^ \ast + \omega } )\exp(- i\psi_{\boldsymbol{\beta}} )$, respectively, wherein *ψ*_1,***β***_ are the characteristic phase lag parameters of the NP plasmons and Mie resonances, respectively. Numerical values for these oscillator parameters are given in Tables S1 and S2 and the particles’ individual resonance profiles are plotted in Figs. S12 and S13. The moments of the system are coupled with strengths $\sigma _{{\boldsymbol{\beta}} \nu }\left( {{{{\mathbf{r}}}}_0} \right) = \left( {e^2/a_2^3} \right){{{\hat{\mathbf{e}}}}}_\nu \cdot {{{\mathbf{X}}}}_{\boldsymbol{\beta}} \left( {{{{\mathbf{r}}}}_0,k_{\boldsymbol{\beta}}} \right)$, wherein ${{{\hat{\mathbf e}}}}_\nu$ is the *ν*-oriented unit vector, ${{{\mathbf{X}}}}_{\boldsymbol{\beta}} \left( {{{{\mathbf{r}}}},k_{\boldsymbol{\beta}}} \right)$ is the vector spherical harmonic that describes the spatial variations of the fields of the ***β***^th^ mode, and $k_{\boldsymbol{\beta}} = \omega _{\boldsymbol{\beta}} /c$. In the case where the couplings strengths are set to zero, the LiNbO_3_ and Au NPs radiate independently and no energy passes between them. As the magnitude of $\sigma _{{\boldsymbol{\beta}} \nu }\left( {{{{\mathbf{r}}}}_0} \right)$ increases, the likelihood that a photon will be exchanged between the LiNbO_3_ and Au multiple times before being radiated away also increases.

One can see from the scattering spectra of Fig. 3b that no mode splitting is generated by the addition of the Au NPs to the LiNbO_3_ sphere in our system. Splitting is readily observed in strongly-coupled NLO polariton systems^46^, such that weak interactions between the Au NPs and microsphere are expected. The theory is in good agreement with this observation, as the weak coupling condition^47,48^ $2a_2^3| {\sigma _{{\boldsymbol{\beta}} \nu }( {{{{\mathbf{r}}}}_0} )} |\sqrt {f_{\boldsymbol{\beta}} f_\nu } /e^2( {\gamma _1 + \gamma _{\boldsymbol{\beta}}} )\sqrt {\omega_{\boldsymbol{\beta}}\omega _1} \,< \, 0.1$ is satisfied for all **r**_0_. The equations of motion of the LiNbO_3_ Mie modes can, thus, be solved perturbatively to capture the effects of these exchanges on the power radiated from the microsphere.

Explicitly, letting $\rho _{\boldsymbol{\beta}} (\omega ) = \mathop {\sum}\nolimits_{n = 1}^{\infty} {\rho_{\boldsymbol{\beta}}^{\left( n \right)}(\omega )}$ where each order in the expansion includes the effects of *n* exchanges of energy, the power scattered from the ***β***^th^ mode is $P_{\boldsymbol{\beta}} (2\omega _0) = ( {c^3/2\pi ^2\omega _0^2\omega_{\boldsymbol{\beta}} a_2^{2\ell + 4}} ){\int}_{ - \pi /2\omega _0}^{\pi /2\omega _0} {{\mathrm{Re}} \{ {\dot \rho_{\boldsymbol{\beta}} ( t )} \}^2{{{\mathrm{d}}}}t}$ (SI, Section S.2.3). The power scattered by ***β*** in the absence of the NP dipoles, $P_{\boldsymbol{\beta}}^{( 1 )}(2\omega _0)$, can be calculated simply by replacing $\rho_{\boldsymbol{\beta}} (t)$ with $\rho_{\boldsymbol{\beta}}^{( 1 )}(t)$.

Due to a convenient orthogonality condition (Eq. [S18]), the total power scattered by the microsphere at the SH frequency, $P(2\omega _0)$, is then simply a sum over the power scattered from each mode, such that $P(2\omega _0) = \mathop {\sum}\nolimits_{\boldsymbol{\beta}} {P_{\boldsymbol{\beta}} (2\omega _0)}$, and the scattering enhancement can be simply defined as $P(2\omega _0)/P^{\left( 1 \right)}(2\omega _0)$, wherein $P^{( 1 )}(2\omega _0) = \mathop {\sum}\nolimits_{\boldsymbol{\beta}} {P_{\boldsymbol{\beta}}^{( 1 )}(2\omega _0)}$ is the total SH power scattered from a bare LiNbO_3_ sphere. In good agreement with the experiment (see Fig. S10), this ratio is very nearly 1 for a model with a single NP due to the weak coupling between the microsphere and the dipole of the plasmons. However, when generalizing to a model with *N* = 1000 NPs in the ensemble (SI, Section S.1), the enhancement ratio reaches a peak value of ~30 in the analyzed SH frequency range and reflects the underlying resonant structure of the Mie modes.

Figure 4c shows these results in detail, demonstrating the excellent quantitative agreement between the SHG enhancement theory and the experimental data. Importantly, the model provides not only a numerical reproduction of the enhancement peaks but straightforwardly explains their origin. Beginning with the lowest-order nontrivial terms in the perturbation series of the Mie resonance ***β***, one can see that the second-order term $\rho_{\boldsymbol{\beta}} ^{\left( 2 \right)}$ describes the transfer of energy from the ${\boldsymbol{\cal{E}}}_0$-induced dipoles of the Au NP to a Mie resonance of the LiNbO_3_ sphere, a process that is impossible without the presence of the NP ensemble and, as described above, represents a newly discovered pathway through which energy can be transferred from the energy bath of the laser to the LiNbO_3_ particle and enhance the overall SHG signal. Accordingly, the third-order term describes the process in which energy is transferred from one Mie mode to another through two separate energy exchanges with the NP ensemble, a process which tends to shuttle energy from broad, more strongly-pumped Mie modes to narrower resonances more weakly excited by the upconversion process.

Higher-order processes can be derived from the analytical solution to $\rho_{\boldsymbol{\beta}}^{\left( n \right)}(\omega )$ but contribute increasingly small corrections to the total LiNbO_3_ polarization, as is shown in the SI, Section S.1. Further, after comparing the magnitudes of the forces of the system, one can conclude that the Au NP ensemble contributes to the enhancement of SHG primarily by mediating energy transfer from the broader, lower-order Mie resonances to their narrower counterparts. The second-order terms do add a small boost to SHG through the augmentations $\sigma _{{\boldsymbol{\beta}} \nu }\left( {{{{\mathbf{r}}}}_0} \right)F_{1\nu }({{{\mathbf{r}}}}_0,\omega )$ to the driving forces of each mode (SI, Section S.1), but while their total contribution is important for relatively broad modes ***β***, it is minor for narrow Mie resonances. Figure S14 shows this effect explicitly, with the total enhancement signal well-approximated by a second-order expansion in regions of small enhancement but requiring third-order corrections near the high-order mode resonance positions.

Our second (ii) hypothesis, in contrast to the coupling model developed above, is concerned with the radiation from the Au NPs themselves. Generally, Au NPs less than ~30 nm in diameter can be assumed to be weak scatterers as their characteristic length scale lies below one tenth of their surface plasmon resonance wavelength, implying that our choice to this point to neglect light emission from the NP ensemble is appropriate. However, small dipole oscillators such as our NPs are known to exhibit superradiance, an effect which is observed when coupling between resonant emitters causes each member of a large ensemble to emit more light than it would on its own. This phenomenon has been observed recently in collections of coupled dipole emitters in many prior investigations^43,44^ and thus is of interest here.

To evaluate whether the radiation from each NP in the experimental ensemble is boosted sufficiently by inter-NP interactions to generate overall SHG enhancement from the collections of NPs in our experiments, we combine per-particle optical cross-sections collected from multiple-Mie scattering simulations^49^ with analytical models to calculate the time-averaged power radiated at the SH from the “typical” NP in square ensembles of sizes *N* = 1 through 225. We label this power as $P_{{{{\mathrm{pl}}}}}(2\omega _0)$, such that the power radiated from the ensemble is $NP_{{{{\mathrm{pl}}}}}(2\omega _0)$. With the details left for the SI, Section S.2.3, the ratio of the powers scattered from the ensemble and the bare LiNbO_3_ particle is then given by:

2 $$\frac{{NP_{{{{\mathrm{pl}}}}}(2\omega _0)}}{{P^{\left( 1 \right)}(2\omega _0)}} = \frac{{2\pi \omega _0NA\left( N \right)\gamma _1^{{{{\mathrm{rad}}}}}f_1f_2a_1^3}}{{c^3\gamma _1^2\gamma _2}}\kappa \left( {2\omega _0} \right)$$

Here, $\kappa (2\omega _0)$ is a spectral profile (Eq. [S34]) that reflects the resonance structure of the LiNbO_3_ modes and has a small maximum value of ~10^−3^. Further, *A*(*N*) is a phenomenological scattering enhancement factor of the Au NPs extracted from the data in Fig. S15a that builds in superradiance effects and corresponds to a value ~300 for *N*~1000 that is not greatly influenced by ensemble disorder (see Fig. S15b).

Combined with the radiative contribution to the damping rate of the Au NPs $\gamma _1^{{{{\mathrm{rad}}}}}\sim 10^{ - 4}\gamma _1$, the mean Mie oscillator strength *f*_2_ and damping rate *γ*_2_, and the rest of the previously defined constants, the superradiant enhancement factor does not overcome the small value of $a_1^3\kappa (2\omega _0)$ to provide an overall scattering boost from the NP ensemble. Explicitly, $NP_{{{{\mathrm{pl}}}}}(2\omega _0)/P^{\left( 1 \right)}\left( {2\omega _0} \right) \ll 1$ for all relevant wavelengths, varying between ~2 × 10^−2^ and 3 × 10^−2^ for 2*ω*_0_ in the visible spectrum. Superradiance, therefore, cannot account for the observed enhancements to SHG.

## Discussion

The invention of efficient miniaturized NLO devices remains an open and important challenge in the field of nanophotonics. Moreover, with current strategies for the maximization of NLO signal strength at the nanoscale are limited by the difficulties and expense of precisely nanoengineering single NLO nano- and microstructures, multiply-resonant nanoantenna systems, and/or individual electric field hotspots, new hybrid nanomaterials that can provide meaningful NLO enhancements without unduly complicated fabrication processes are highly desirable. To this end, we have designed a new class of disordered Au–LiNbO_3_ NLO nanostructures that are straightforward to synthesize and demonstrate state-of-the-art enhancements. These hybrid structures were prepared through a high-throughput solution-phase self-assembly process through which the mesoporous structures of micrometer-scale LiNbO_3_ spheres were coated with a disperse layer of ~10 nm diameter Au NPs with a highly tunable density (~6 to 1000 Au NPs/LiNbO_3_ sphere) and an even distribution.

Single nanostructures were then isolated, characterized, and modeled in order to interrogate the mechanisms governing the enhancement of their SHG. Broadband linear and nonlinear optical scattering and extinction measurements of these hybrid structures demonstrate Mie-like resonant behaviors within the LiNbO_3_ and minimal rearrangement of the LiNbO_3_ resonance structure by the Au NPs. The Mie-like behaviors are consistent with prior investigations of multicrystalline NLO microspheres^13^. Further, analytical and numerical models predict that the coupling of each deeply subwavelength, nonradiative NP with its neighboring NPs and the microsphere is weak, such that modifications of the microsphere’s spectrum should, in agreement with observations, be minimal. These models also reveal that SHG enhancement occurs within the hybrid structures through a previously unexplored near-field coupling mechanism, available only at the nanoscale, that enhances the conversion of energy from incoming light into NLO material resonances and then confines it within the nanostructures’ narrow resonances.

As a result, the hybrid materials described herein broaden the optical nanomaterial design space by highlighting the NLO enhancement capabilities of subwavelength or otherwise nonradiative nanoantenna systems. For example, the observable SH resonances of the LiNbO_3_ are long-lived with $\gamma_{\boldsymbol{\beta}}/\omega_{\boldsymbol{\beta}} \sim 10^{ - 3}$ to 10^−2^ such that they are candidates for use in sensing and light emission applications where measurable impurity-induced peak shifts and/or tunable monochromatic emission at the nanoscale are desired. We have shown that the SH excitation of such narrow Mie resonances is preferentially and significantly enhanced by weak coupling to a relatively low and disordered volume of a second resonant material (Au) that is resonant at the output SH frequency of the LiNbO_3_ sphere. Therefore, new design strategies for sensors and emitters can specifically incorporate imprecise ensembles of very small nanoantennas as needed to optimize the NLO signal intensity.

In addition, the sensitivity of the SH Mie resonances’ output frequencies to intrinsic and environmental defects is not degraded by the presence of the Au NPs. As evidence, the strong dependence of the microsphere’s Mie resonance spectral positions, widths, and heights on its geometry and dielectric function is preserved in hybrid structures, as is shown in Fig. 3b. This preservation is supported by the excellent agreement with the model, which specifies that the weak coupling between the Mie resonances and plasmon dipoles precludes rearrangement or obfuscation of the LiNbO_3_ Mie spectrum.

Determination of the exact value of the dielectric function *ϵ*_2_ of the structures analyzed in the experiment is difficult, as the mesoporous material is novel, single-particle ellipsometry through e.g. electron beam spectroscopy^50^ is in its infancy, and substrate effects can cause nontrivial reorganization of the sphere’s Mie spectrum (SI, Section S.2.2). However, as is detailed in the SI, Section S.2.1, the estimate *ϵ*_2_ = 5.5 + 0.035*i* used alongside the chosen value *a*_2_ = 500 nm as well as the HAADF images and linear and nonlinear scattering spectra of Figs. 1, 3, and 4 produces uniquely precise agreement between theory, experiment, and prior art^45^ in the SH spectral region. Variation of either *a*_2_ or *ϵ*_2_ by ~5% can generate noticeable ~15 to 35 nm shifts in the Mie resonance positions of both bare LiNbO_3_ and hybrid structures (see Figs. 4d and S14c), such that we take ±5% to be a rough estimate of the uncertainty in the microsphere parameters. The small (~25 nm) phenomenological shifts made to align the theory with the experimental data of Fig. 4c fall well within these bounds.

By contrast, the Mie resonances are *not* particularly sensitive to the parameters of the NP plasmon resonances. The NPs’ extinction profiles simply provide a spectral envelope inside which SHG enhancements are possible. Finally, the microsphere’s surface coverage by the NP ensemble is limited, allowing for ready interaction between external NPs, cavities, waveguides, molecules, etc. and the LiNbO_3_.

Future advancements in the fabrication and characterization processes of our (or similar) nanostructures that provide greater control over simple parameters like the microsphere’s shape and internal nanocrystal density will allow researchers to more precisely tune these systems. Further, the simplicity of the coupling phenomena governing the SHG output intensity enhancements suggests that nanostructures displaying NLO behaviors beyond SHG should be able to take advantage of the same effects. In combination with the flexible perturbative model developed here, our results suggest that nanoscale NLO enhancements can be straightforwardly modeled, designed, and realized in a wide array of near-field coupled nanoscopic systems displaying all manner of frequency conversion behaviors without the need for expensive nanoengineering.

## Materials and methods

Further details regarding the synthesis, characterization, and modeling of the nanostructures used in this work can be found in the SI.

## Supplementary information

Supplemental Information File

## Data Availability

All data needed to evaluate the conclusions in the paper are present in the paper and/or the Supplementary Information.
