# Supplementary material for: Near-field enhancement of optical second harmonic generation in hybrid gold–lithium niobate nanostructures
Source: Light Sci Appl. 2023 Apr 25;12:99. doi: 10.1038/s41377-023-01092-8 (PMC10130160; doi:10.1038/s41377-023-01092-8)
Supplement: Supplementary file 1 — Supplemental Information File [file 41377_2023_1092_MOESM1_ESM.pdf]

Supplementary Information for

Near-Field Enhancement of Optical Second Harmonic Generation in Hybrid Gold-Lithium Niobate Nanostructures

Rana Faryad Ali, Jacob A. Busche, Saeid Kamal, David J. Masiello, and Byron D. Gates*

*Corresponding author. Email: bgates@sfu.ca

This document includes:

- Section S.1: Materials and Methods, which provides brief details of the experimental and theoretical procedures used to generate the observables in the main text. These details are separated into subsections
  - Subsection S.1.1, which discusses in greater depth the synthesis and characterization of the hybrid Au-$LiNbO_3$ particles of this investigation
  - Subsection S.1.2, which outlines the derivation of the primary mathematical conclusions of the main text
- Section S.2: Additional Theoretical Details, which contains a long-form discussion of the construction of the near-field coupling and superradiance models. This section contains subsections
  - Subsection S.2.1, which details the material models used in this investigation
  - Subsection S.2.2, which provides derivations of the oscillator models of the bare Mie and plasmon resonances
  - Subsection S.2.3, which provides all scattering calculations for the main text
  - Subsection S.2.4, which shows the derivation of the coupled-oscillator model of the hybrid nanostructures from first principles
- Section S.3: Additional Figures and Tables, which contains supplemental figures and tables for reference, specifically
  - Figures S1 to S16
  - Tables S1 to S4

S.1 Materials and Methods

S.1.1 Experimental Details

S.1.1.1 Synthesis of Mesoporous Monodisperse Lithium Niobtate Particles

All chemicals were used without further purification. Monodisperse lithium niobate particles were synthesized using our previously reported method. In brief, 0.8 mM of niobium n-butoxide [$Nb(OBu)_5$, 99%, Alfa Aesar] was dissolved in 4 mL of ethanol. This solution was aged for 36 h in a desiccator in the presence of an open glass vial containing 25 mL of water to generate a humid atmosphere. The resulting gel-like precursor was mixed with 20 mL of an aqueous solution of 0.1 M lithium hydroxide monohydrate ($LiOH{\cdot}H_2O$, 99%, Alfa Aesar) and sonicated for 20 min. A 10 mL aliquot of the resulting suspension was transferred to a 23 mL Teflon-lined autoclave (Model No. 4749, Parr Instruments Co., Moline, IL USA) and heated at 200 °C for 48 h. After cooling to room temperature, white precipitates were isolated from the solution via a process of centrifugation (Model No. AccuSpin 400, Fisher Scientific) at 8,000 rpm for 15 min and decanting of the solution. These solids were washed by re-suspending them in 10 mL deionized water (18 MΩ·cm, produced using a Barnstead NANOpure DIamond water filtration system). This purification process was repeated for a total of three times. The purified product was dried at 70 °C for 10 h to remove residual water prior to further analyses. The dried precipitates were calcined by heating from room temperature to 600 °C at a rate of 5 °C min^{-1} and held at 600 °C for 45 min to induce complete crystallization.

S.1.1.2 Synthesis of Gold-Lithium Niobate Hybrid Nanostructures

An aqueous suspension of porous, monodisperse $LiNbO_3$ particles (0.5 mg mL^{-1}) was added and dispersed in 5 mL of water via sonication. Into this suspension, either 1 mL (for a higher loading of gold nanoparticles) or 0.1 mL (for a lower laoding of gold nanoparticles) of a 5 mM aqueous solution of gold(III) chloride trihydrate ($HAuCl_4 \cdot 3H_2O$, 99.9%, Sigma Aldrich) was added, and the mixture stirred at 70 °C for 3 h. This step was followed by the addition of 1 mL or 0.01 mL of 0.5% (w v^{-1}) trisodium citrate dihydrate ($C_6H_5Na_3O_7 \cdot 2H_2O$, Sigma Aldrich, ≥99%) aqueous solution into the reaction mixture. The reaction mixture was stirred further at 70 °C for 1 h. The precipitates were washed two times with water. The as-synthesized hybrid nanostructures of $LiNbO_3$ with gold (Au) nanoparticles (NPs) were isolated from the unreacted excess gold salts and unbound Au NPs through centrifugation (Thermo Electron Corporation, IEC microlite microcentrifuge) at 2,000 rpm for 3 min, decanting of the supernatants, and re-dispersion of the isolated solids in DI water with the assistance of a vortexer for 3 min.

The morphology and dimensions of the $LiNbO_3$ particles were characterized using an FEI Osiris X-FEG 8 scanning/transmission electron microscope (TEM/STEM) operated at an accelerating voltage of 200 kV. Samples for TEM/STEM analyses were prepared by dispersing the purified products in ethanol followed by drop-casting 5 μL of each suspension onto separate TEM grids (300 mesh copper grids coated with Formvar/carbon) purchased from Cedarlane Labs. Each TEM grid was dried at ∼230 Torr for at least 20 min prior to analysis. Energy dispersive X-ray spectroscopy (EDS) analyses were performed using the FEI

Osiris scanning/TEM, which was equipped with a Super-X EDS system with ChemiSTEM Technology integrating the signal from four spectrometers.

Purity, crystallinity, and phase of the $LiNbO_3$ particles were characterized using Raman spectroscopy and powder X-ray diffraction (XRD) techniques. Raman spectra were collected using a Renishaw inVia Raman microscope with a 50× SWD objective lens (Leica, 0.5 NA), and a 514 nm laser (argon-ion laser, Model No. Stellar-Pro 514/50) set to 100% laser power with an exposure time of 30 s. The Raman spectrometer was calibrated by collecting the Raman spectrum of a polished silicon (Si) standard with a distinct peak centred at 520 cm^{-1}. The Raman spectra for the samples were acquired from 100 to 1,000 cm^{-1} using a grating with 1,800 lines mm^{-1}. The XRD patterns of the samples were acquired with a Rigaku R-Axis Rapid diffractometer equipped with a 3 kW sealed tube copper source (Kα radiation, $\lambda = 0.15418$ nm) collimated to 0.5 mm. The samples were packed into a cylindrical recess drilled into glass microscope slides (Leica 1 mm Surgipath Snowcoat X-tra Micro Slides) for acquiring XRD patterns for the products.

The optical absorption spectra of the products were measured using an Agilent Technologies ultraviolet-visible spectrophotometer (Agilent 8453, Model No. G1103). For these measurements, the samples were suspended in water and held in 1 cm path length poly(methyl methacrylate) cuvettes (VWRTM, Catalog No. 634-8537). The linear spectra of individual pristine $LiNbO_3$ and hybrid Au-$LiNbO_3$ particles were measured using a Zeiss M1m optical microscope operating in a dark-field spectroscopy setup. The light from a halogen lamp was focused onto the sample through a dark field objective (50×, Zeiss Epiplan Neofluar). The scattered light was collected by the same objective and detected by an imaging spectrometer (Princeton Instrument Acton spectrometer equipped with a PIXIS 400 CCD detector cooled to –72°C). The linear scattering cross section spectra were obtained by normalizing the signal from a single particle to the signal coming directly from the lamp, after subtraction of the background in proximity to the particle.

The second harmonic generation (SHG) activity of the individual pristine $LiNbO_3$ and hybrid Au-$LiNbO_3$ particles was assessed using a Leica SP5 laser scanning confocal two photon microscope equipped with a Coherent Chameleon Vision II laser and a Zeiss LSM 510 MP confocal microscope. A dilute dispersion of nanostructures was drop-cast onto glass coverslips and brought into the focal point of the microscope. The SHG response was characterized by locating individual particles using a 63× oil immersion objective aperture and scanning the laser excitation from 850 nm to 1,070 nm as the fundamental wavelength.

S.1.2 Theoretical Calculations

S.1.2.1 Bare Lithium Niobate Sphere Characterization

Modeling the microsphere as a smooth, isotropic spherical particle, the nearly constant dielectric function of $LiNbO_3$ in the optical region of interest[1] allows the microsphere's linear response near the SH to be described by the excitation of a set of 21 Mie resonances (SI, Section S.2.1.2). We have established $\boldsymbol{\beta} = \{T', p', \ell', m'\}$ as a collective index to describe these modes such that the scattered electric field of each mode can be expanded as $\boldsymbol{\mathcal{E}}_{\text{sca}}(\mathbf{r}, \omega) = \sum_{\boldsymbol{\beta}} a_2^{-\ell-2} \left[\rho_{\boldsymbol{\beta}}(\omega)\mathbf{X}_{\boldsymbol{\beta}}(\mathbf{r}, k_{\boldsymbol{\beta}}) + \rho^*_{\boldsymbol{\beta}}(-\omega)\mathbf{X}^*_{\boldsymbol{\beta}}(\mathbf{r}, k_{\boldsymbol{\beta}})\right]$.[2]

Using $\boldsymbol{\mathcal{E}}_{\text{sca}}$ along with the magnetic fields of each mode, $\boldsymbol{\mathcal{B}}_{\text{sca}}(\mathbf{r}, \omega) = (c/i\omega)\nabla \times \boldsymbol{\mathcal{E}}_{\text{sca}}(\mathbf{r}, \omega)$,

the scattered power averaged over a period of the SH frequency can be calculated from Poynting's theorem as (Section S.2.3.1):

$$P^{(1)}(2\omega_0) = \frac{c^3 E_0^2}{4\pi\omega_0} \sum_{\boldsymbol{\beta}} \frac{1 - \delta_{p1}\delta_{m0}}{\omega_{\boldsymbol{\beta}} a_2^{2\ell+4}} |C_{\boldsymbol{\beta}}(\omega_0)|^2 |\alpha_{\boldsymbol{\beta}}(2\omega_0)|^2 \tag{S1}$$

Here, $\alpha_{\boldsymbol{\beta}}(\omega) = (a_2^{\ell+2} f_{\boldsymbol{\beta}}/2\omega_{\boldsymbol{\beta}}) \exp(i\psi_{\boldsymbol{\beta}})/(\omega_{\boldsymbol{\beta}} - i\gamma_{\boldsymbol{\beta}}/2 - \omega)$ is the linear polarizability of the mode $\boldsymbol{\beta}$, with $C_{\boldsymbol{\beta}}(\omega_0) \sim E_0 \chi_2^{(2)}(\omega_0, \omega_0)$ a unitless overlap coefficient. In general, $C_{\boldsymbol{\beta}}(\omega_0)$ quantifies the efficiency of photon upconversion between the fundamental resonances of the system at ω_0 and a resonance $\boldsymbol{\beta}$ at the SH, and thus is large only if the fundamental resonances are driven strongly by the incident light. Also, $C_{\boldsymbol{\beta}}(\omega_0)$ is zero unless the fundamental and SH resonances satisfy specific symmetry requirements, which are detailed below. Finally, the explicit form of this overlap coefficient is complicated, but described in full in Section S.2.4.2, Eq. (S56).

In general, the resonance frequencies of the Mie modes are independent of p and m, although these indices do determine the strength of the response of a mode to a driving source of a given spatial symmetry. In detail, if we allow $\boldsymbol{\alpha} = \{T, p, \ell, m\}$ to be the collective index of one fundamental resonance of the $LiNbO_3$ particle and $\boldsymbol{\alpha}' = \{T'', p'', \ell'', m''\}$ to be the index of another, in the case where the sphere has a weak, isotropic, and frequency-dependent nonlinear susceptibility $\boldsymbol{\chi}_2^{(2)}(\omega', \omega - \omega') = \mathbf{1}_3 \chi_2^{(2)}(\omega', \omega - \omega')$ only modes $\boldsymbol{\beta}$ for which $p + p' + p''$ is even and $m' \pm m \pm m'' = 0$ are driven by SHG. For example, with the microsphere driven by an x-polarized plane wave at the fundamental frequency such that $\boldsymbol{\alpha} = \{E, 0, \ell, 1\}$ or $\{M, 1, \ell, 1\}$, each of the 21 SH modes in the model has an index pair $(p', m') = (0, 0)$, $(0, 2)$, or $(1, 2)$. In contrast, the dependence of a mode's response on T' and ℓ' is determined by its spectral overlap with the source. In the energy window of the observed SHG enhancement, seven sets of modes can be significantly driven by the upconversion process: four sets of electric modes with $\ell' = 6$, 7, 9, and 10, and three sets of magnetic modes with $\ell' = 7$, 10, and 11. See Figure S13 for details.

S.1.2.2 Characterization of the Lithium Niobate Radius and Dielectric Function

Section S.2.2 details the characterization of each of the $LiNbO_3$ Mie modes, each of which is assigned a resonance frequency $\omega_{\boldsymbol{\beta}}$, a damping rate $\gamma_{\boldsymbol{\beta}}$, and an oscillator strength $f_{\boldsymbol{\beta}}$ (equivalently, an effective mass $\mu_{\boldsymbol{\beta}} = e^2/a_2^3 f_{\boldsymbol{\beta}}$) that determine its spectral position and response magnitude to external stimuli. This characterization is performed using a dielectric function $\epsilon_2 = 5.5 + 0.035i$ and a radius $a_2 = 500$ nm estimated from the experiment. The real part of ϵ_2 is taken from estimates extracted from a set of nine single-particle scattering experiments conducted on bare $LiNbO_3$ spheres of radii 350 to 1000 nm. Figure 3c shows results from one representative scattering experiment, with the remainder of the results given Figure S17 and in Table S3. Further, in conjunction with the choice of radius, the choice $\mathrm{Re}\{\epsilon_2\} = 5.5$ provides the best overlap between the spectral locations of the Mie resonances and the SHG enhancement peaks seen in Figure 4c, as well as the best agreement between the relative peak heights. We note that small ($\sim 5\%$) increases (decreases) to $\mathrm{Re}\{\epsilon_2\}$ and a_2 lead to red (blue) shifts in the Mie resonance positions that can nullify each other, such appropriate choices with the ranges $a_2 \pm 20$ nm and $\mathrm{Re}\{\epsilon_2\} \pm 0.1$ are likely to lead to similar

results. The inset of Figure 4c shows the range of Mie resonance energies available with $\pm 4\%$ changes to a_2, and analogous results when varying $\mathrm{Re}\{\epsilon_2\}$ are shown in Figure S14c.

The imaginary part of ϵ_2, which is expected to be small,[1] is chosen to agree well with the linewidths of the modes observed in the second harmonic data of Figure 4c. The exact value has yet to be characterized via e.g. ellipsometry[3] and is difficult to estimate from the scattering data due to substrate effects (see Figure S13a). The linewidths of the Mie resonances depend sensitively on the rate of internal material losses (see Figure S14d) and the heights of the narrow Mie modes in both scattering and SHG enhancement spectra can vary noticeably with $\sim 5\%$ changes to $\mathrm{Im}\{\epsilon_2\}$. However, small changes to $\mathrm{Im}\{\epsilon_2\}$ do not modify the peak positions within the SH enhancement spectrum such that we take $\mathrm{Im}\{\epsilon_2\}$ to be a fitting parameter that affects the overall magnitude of the theoretical SHG enhancements, but does not qualitatively affect the results. Further details are given in Section S.2.1.

S.1.2.3 Superradiant Nanoparticle Scattering

Each NP is assumed to be spherical and isotropic with a Drude-Lorentz dielectric function $\epsilon_1(\omega)$ fit to the Au dielectric data of Ref. 4 (Section S.2.1, Figure S16, and Table S4) and an associated set of surface plasmon resonances within the spectral window of the observed SH emission. Figure 3a demonstrates the excellent agreement between the resulting theoretical absorption cross section of the NP dipole plasmons using this model and extinction measurements performed on single hybrid nanostructures, highlighting the validity of the model and the uniformity of the NP responses in the experiment. We thus restrict our analysis to include only the dipole resonances of the NPs, which we label $\mathbf{d}_i(\omega)$, and allow each particle to lie at a position $\mathbf{r}_i$ on the surface of the $LiNbO_3$ microsphere.

The dynamics of the plasmon dipoles are inferred from numerical scattering calculations using both multiple-Mie scattering and boundary element method Maxwell solvers.[5,6] These simulations were performed on arrays of 10-nm diameter Au NPs in square grids with a 20 nm center-to-center spacing, as complete layers on spherical templates containing the $N \approx 1000$ Au NPs present in the experiment are too large to compute directly. Moreover, grids reliably approximate the effects of interparticle coupling in the limit that the radii of the Au NPs and their separations are much smaller than the radius of the $LiNbO_3$ microsphere. Using these scaled down simulations, we extrapolated from smaller ensembles ($N \leq 225$) the behaviors of larger collections of Au NPs. For comparative reasons, we used both regular grids and randomized grids of Au NPs to evaluate the effects of disorder on the amount of light scattered by each particle.

Our results (Figure S15a) show that the maximum scattering cross section of each dipole grows roughly two orders of magnitude from 7.75×10^{-3} nm^2 as N varies from 1 to 1000, and that the exact magnitude of the growth depends on whether the dipole is oriented perpendicular (maximum of 0.355 nm^2) or parallel (1.18 nm^2) to the plane of the ensemble. Conversely, the absorption cross section maxima of each dipole converge quickly with ensemble size, as shown in Figure S15a, moving from a single particle value of 13.5 nm^2 to perpendicular and the parallel values of 10.3 nm^2 and 15.7 nm^2, respectively, for ensembles of $N > 50$ NPs. In addition, while randomness in the positions of the particles can modulate the per-particle scattering cross section maxima by $\pm 20\%$ in comparison to a regular grid (Figure S14b), an extreme value distribution fit to the recorded scattering cross section

maxima of 100 simulations with randomized grids of $N = 16$ NPs shows that modifications beyond this range occur in ∼0.4% of NPs.

The regular-grid simulations are, thus, taken to be representative of the ensemble as a whole. Further, Figure S15b shows that while the peak maxima of the scattering observables depend strongly on N, their lineshapes do not. Therefore, with the NPs' scattering strongly modified and their absorption changed relatively little with increasing N, we conclude that the dipole oscillator strengths are best estimated from the absorption data. Explicitly, each dipole is then treated quasistatically with a position- and orientation-dependent polarizability (see Section S.2.2.2), while the ensemble-induced scattering enhancements are included phenomenologically through modification of the Rayleigh scattering rate of each dipole by a positive factor $A(N)$. When driven by the scattered fields of a bare $LiNbO_3$ sphere, the ensemble then radiates a time-averaged scattered power

$$P_{\text{pl}}(2\omega_0) \approx A(N)\frac{\gamma_1^{\text{rad}} f_1 a_1^3 E_0^2}{2\gamma_1^2}\sum_{\boldsymbol{\beta}} \frac{K_{\boldsymbol{\beta}}}{a_2^{2\ell+4}} \left|C_{\boldsymbol{\beta}}(\omega_0)\right|^2 \left|\alpha_{\boldsymbol{\beta}}(2\omega_0)\right|^2 \tag{S2}$$

Here, f_1 is the orientation-averaged oscillator strength of the NP dipoles (Eq. [S13]), and $K_{\boldsymbol{\beta}}$ (Eq. [S29]) is an overlap coefficient that determines the strength of the ensemble's response to the $LiNbO_3$ fields.

See Section S.2.3 for further details.

S.1.2.4 SHG Enhancement via Near-Field Coupling

The first and second-order terms in the solution of the Mie resonance equations of motion in Eq. (1) are

$$\begin{aligned}
\rho_{\boldsymbol{\beta}}^{(1)}(\omega) &= \frac{f_{\boldsymbol{\beta}} a_2^{\ell+2}(\Omega_{\boldsymbol{\beta}}^* + \omega)\mathrm{e}^{-i\psi_{\boldsymbol{\beta}}}}{2\omega_{\boldsymbol{\beta}} e\left(|\Omega_{\boldsymbol{\beta}}|^2 - \omega^2 - i\omega\gamma_{\boldsymbol{\beta}}\right)} F_{2\boldsymbol{\beta}}(\omega) \\
\rho_{\boldsymbol{\beta}}^{(2)}(\omega) &= \frac{f_{\boldsymbol{\beta}} a_2^{\ell+2}(\Omega_{\boldsymbol{\beta}}^* + \omega)\mathrm{e}^{-i\psi_{\boldsymbol{\beta}}}}{2\omega_{\boldsymbol{\beta}} e^2\left(|\Omega_{\boldsymbol{\beta}}|^2 - \omega^2 - i\omega\gamma_{\boldsymbol{\beta}}\right)} \\
&\quad \times \frac{e f_\nu a_1^3 \eta_1(\omega)}{2\omega_1 e^2\left(|\Omega_1|^2 - \omega^2 - i\omega\gamma_1\right)} \sum_\nu \sigma_{\boldsymbol{\beta}\nu}(\mathbf{r}_0) F_{1\nu}(\mathbf{r}_0, \omega)
\end{aligned} \tag{S3}$$

which detail the driving of each mode $\boldsymbol{\beta}$ through direct upconversion and through excitation of the NP by the upconversion process, respectively. Higher-order terms build in the consequences of multiple transfers of energy between the microsphere and the NP and become increasingly tedious to write explicitly, but can be analyzed compactly with

$$\begin{aligned}
\rho_{\boldsymbol{\beta}}^{(n>2)}(\omega) = \sum_\nu \sum_{\boldsymbol{\beta}' \neq \boldsymbol{\beta}} & \frac{f_{\boldsymbol{\beta}} f_\nu a_2^{\ell+2} a_1^3}{4e^4 \omega_{\boldsymbol{\beta}} \omega_1} \frac{\eta_1(\omega)\eta_{\boldsymbol{\beta}}(\omega)}{\left(|\Omega_{\boldsymbol{\beta}}|^2 - \omega^2 - i\omega\gamma_{\boldsymbol{\beta}}\right)\left(|\Omega_1|^2 - \omega^2 - i\omega\gamma_1\right)} \\
\times & \frac{\sigma_{\boldsymbol{\beta}\nu}(\mathbf{r}_0)}{a_2^{\ell'-1}} \left[\sigma_{\boldsymbol{\beta}'\nu}(\mathbf{r}_0)\rho_{\boldsymbol{\beta}'}^{(n-2)}(\omega) + \sigma_{\boldsymbol{\beta}'\nu}^*(\mathbf{r}_0)\rho_{\boldsymbol{\beta}'}^{(n-2)*}(-\omega)\right]
\end{aligned} \tag{S4}$$

from which it is clear that, with $|\rho_{\boldsymbol{\beta}}^{(2)}(\omega)| \ll |\rho_{\boldsymbol{\beta}}^{(1)}(\omega)|$, terms beyond the third order provide only small corrections.

To account for all N NPs, we label each NP with the index $i \in [1, N]$ such that $\mathbf{r}_0 \rightarrow \mathbf{r}_i$ and $d_\nu(\omega) \rightarrow d_{i\nu}(\omega)$, but allow the NPs to have identical radii and their plasmons to have identical resonance frequencies, linewidths, phase offsets, and oscillator strengths. Therefore, the coupling term in Eq. (1) is replaced by the sum $\sum_{i\nu} \sigma_{\beta\nu}(\mathbf{r}_i) d_{i\nu}(\omega)$ and similar sums in each successive term of the perturbation expansion must be adjusted accordingly. In this work, these sums are carried out by placing three dipoles $d_{i\nu}(\omega)$ at $N = 1{,}000$ points arranged on a sphere of radius $a_1 + a_2$ in a Fibonacci lattice.[7] Calculations with randomized points display only small variations from the results of the regular Fibonacci array in good agreement with the random-ensemble scattering results of Figure 3b and are thus not shown.

S.2 Additional Theoretical Details

S.2.1 Construction of the Material Models from Data

In order to maximize the agreement between our theoretical models and the SH enhancement data, we construct a model dielectric function for Au and a dielectric function and second-order susceptibility and $LiNbO_3$ using our own single particle scattering data, as well as from ellipsometry data and atomistic numerical simulations from Refs. 4 and 8, respectively. The Au details are given in Section S.1.2.2 and the $LiNbO_3$ details are given in Section S.2.1.2.

S.2.1.1 Fitting of the Au Lorentz-Drude Dielectric Model

The oscillator parameters of the dipole plasmons within the Au NPs modeled in this investigation are calculated using a simple Drude-Lorentz model of the dielectric function of gold. This dielectric function is given by

$$\epsilon_1(\omega) = 1 - \frac{\omega_{p1}^2}{\omega^2 + \mathrm{i}\omega\Gamma_1} + \sum_{i=2}^{3} \frac{\omega_{pi}^2}{\Lambda_i^2 - \omega^2 - \mathrm{i}\omega\Gamma_i} \tag{S5}$$

and its form and parameters are inferred from ellipsometry data published in Ref. 4. In particular, as shown in Figure S16a, a Drude-model (ω_{p2}, $\omega_{p3} \rightarrow 0$) fit via nonlinear least squares methods to the gold dielectric function provides a good approximation to the dielectric data for photon energies $\hbar\omega < 2.0$ eV, but this model cannot capture the effects of interband transitions that become increasingly important at higher energies. Indeed, a Drude model is provided by Ref. 4, but it is not used in this analysis as it produces inaccurate predictions of the dipole plasmon energies of Au NPs, which exist well above the 2.0 eV threshold.

To produce a more accurate Au NP model in the region between 2.0 and 2.8 eV, the addition of two Lorentz oscillators to the Drude model dielectric function was found to be sufficient. The NP dipole plasmons have resonance energies near 2.5 eV, well below the upper bound of the single-oscillator Drude-Lorentz model's accuracy near 2.9 eV (see Figure S16a). The parameters for the Drude-Lorentz dielectric model were inferred by first fitting a simplified Drude model

$$\epsilon_1(\omega) \approx -\frac{\omega_{p1}^2}{\omega^2 + \Gamma_1^2} + \mathrm{i}\frac{\omega_{p1}^2 \Gamma_1}{\omega^3 + \omega\Gamma_1} \tag{S6}$$

to the real and imaginary parts of single-crystal dielectric data of Ref. 4 at low energies $\omega \ll \Lambda_2$. These fits produced estimates $\hbar\omega_{p1} = 7.20 \pm 0.9$ eV and $\hbar\Gamma_1 = 71.1 \pm 13$ meV, respectively, which were then used as initial guesses for fits using the more robust Drude-Lorentz model. Focusing on the energy window between 0.8 and 2.8 eV, the Drude-Lorentz fits were made using the regularized function $\mathrm{Re}\{\epsilon_1(\omega)\} + \eta(\omega)\mathrm{Im}\{\epsilon_1(\omega)\}$ to simultaneously fit the model to both components of the complex dielectric data while avoiding bias toward the data's much larger real part in the energy window between 0.8 and 2.4 eV.

Fits using simplex, differential evolution, simulated annealing, and random search fitting methods[9] produced stable results using weighting functions $\eta(\omega) = \lambda_1 \exp(-\lambda_2\omega)$ with $\lambda_1 = 40 \pm 5$ and $\lambda_2 = (6.5 \pm 1) \times 10^{-16}$ s/rad. The central values for either factor are used in this work. Taking the average of the results of the four fitting routines, the model parameters used in the following discussion are given in Table S4.

S.2.1.2 Fitting of the Lithium Niobate Dielectric Model and Nonlinear Susceptibility

The radii of the samples examined in Figure 4c have radii of $a_2 \approx 500$ nm, and we use this value in the following analysis. To estimate ϵ_2, nine scattering spectra were collected using bare $LiNbO_3$ particles with radii between 350 nm and 1000 nm, with the radii estimated from microscopy analyses with a precision of ± 50 nm. Mie theory scattering spectra were compared to the experimental data, but peak splitting and broadening generated by the substrate in the experimental data restricts the utility of the ideal substrate-free model.

More precisely, in agreement with Ref. 10, the presence of a substrate in the scattering spectra generates peak broadening that can in most cases be captured to acceptable approximation by a Mie model with phenomenologically added internal damping. However, as is also demonstrated in Ref. 10, the substrate can also induce splitting, shifting, and even peak amplification that are not uniform and are not reproducible by simple Mie theory. These effects can be seen in Figure S17, where single peaks in a Mie theory model are spectrally aligned with multiply-peaked features in the data, and more obviously in Figure S13. The latter compares Mie theory to simulations that include a substrate, showing that a phenomenologically damped dielectric model reproduces some substrate effects (peak suppression, broadening) but not others (enhancements, shifts of narrow peaks).

Thus, estimates of the real part of ϵ_2 were performed by aligning the visible broad peaks of the theory and experiment in the spectral window between 720 nm and 380 nm, and the absolute peak widths and relative heights were ignored. Narrow features in the data are also ignored as they are most likely to be shifted by the substrate in a manner not captured by Mie theory, as evidenced by the narrow feature near 2.5 eV in Figure S13a. Results using these comparison guidelines are shown in Table S2.

Within the range of possible values of $\mathrm{Re}\{\epsilon_2\}$ shown therein, the value 5.5 provides the best agreement between the spectral positions and relative magnitudes of the SH peaks in the theory and experiment, assuming $a_2 = 500$ nm. As is mentioned in the main text and demonstrated in Figures 4c (inset) and S14c, small tweaks to ϵ_2 and a_2 will provide similar Mie spectra such that the particular values chosen within a narrow range ($\pm \sim 5\%$) are irrelevant to the conclusions of this investigation.

The imaginary part of ϵ_2 is taken to be 0.035. This value is likely larger than the true value

extracted from earlier ellipsometry experiments,[1] as the linewidths of the Mie resonances of the $LiNbO_3$ sphere are increased by the presence of a substrate. Because the magnitude of this increase is difficult to infer from first-principles models, we instead choose $\mathrm{Im}\{\epsilon_2\}$ to agree well with the linewidths of the modes observed in the second harmonic data of Figure 4c,b. Within a range of $\pm \sim 10\%$ of the chosen value, $\mathrm{Im}\{\epsilon_2\}$ simply scales the magnitude of the SH enhancements, such that, like the choices of $\mathrm{Re}\{\epsilon_2\}$ and a_2, the precise value of $\mathrm{Im}\{\epsilon_2\}$ does not affect our conclusions.

The second-order susceptibility of the $LiNbO_3$, $\boldsymbol{\chi}_2^{(2)}(\omega', \omega - \omega')$ was fit to the simulated data from Ref. 8 using an anharmonic oscillator model[11] in the approximation that the $LiNbO_3$ has an isotropic, homogeneous response within the microsphere boundaries. Explicitly, the susceptibility is given by:

$$\begin{aligned} &\boldsymbol{\chi}_2^{(2)}(\omega', \omega - \omega') = \\ &\quad \mathbf{1}\chi_\infty^{(2)} - \mathbf{1}\frac{s}{(\Lambda^2 - \omega^2 - \mathrm{i}\omega\Gamma)(\Lambda^2 - \omega'^2 - \mathrm{i}\omega'\Gamma)(\Lambda^2 - [\omega - \omega']^2 - \mathrm{i}[\omega - \omega']\Gamma)} \end{aligned} \tag{S7}$$

wherein Λ and Γ are the natural frequency and linewidth of the Lorentz oscillator, respectively. These features are used to characterize the response of the $LiNbO_3$ carriers. $\chi_\infty^{(2)}$ is a constant offset that approximates the shift of the real part of $\boldsymbol{\chi}_2^{(2)}$ at low energies by higher-energy transitions. Further, s is the characteristic anharmonic oscillator strength with units cm s^6 statV^{-1} and is taken to be very small such that $sE_0 \ll \Lambda^6$ with E_0 the characteristic strength of the laser field. Finally, $\mathbf{1}_3 = \hat{\mathbf{x}}\hat{\mathbf{x}}\hat{\mathbf{x}} + \hat{\mathbf{y}}\hat{\mathbf{y}}\hat{\mathbf{y}} + \hat{\mathbf{z}}\hat{\mathbf{z}}\hat{\mathbf{z}}$ is the rank-3 identity tensor.

To fit the data, we evaluated the second-order susceptibility in the limit that $\omega = 2\omega'$, i.e. with the assumption that the driving laser at $\omega' = \omega_0$ is very narrow and the observation frequency is always at $2\omega_0$. With this restriction, the expression for the susceptibility simplifies to $\boldsymbol{\chi}_2^{(2)}(\omega_0, \omega_0) = \mathbf{1}\chi_\infty - \mathbf{1}s/(\Lambda^2 - 4\omega_0^2 - 2\mathrm{i}\omega_0\Gamma)(\Lambda^2 - \omega_0^2 - \mathrm{i}\omega_0\Gamma)^2$ and can be easily visualized as is shown in Figure S11.

As was done with the dielectric function of Au in Section S.1.2.2, the model was fit to the real part, imaginary part, and absolute value of the data using simplex, differential evolution, simulated annealing, and random search nonlinear least squares methods. An average of the results of each of the four methods was collected for the fit to each function of the data, and the parameter average that produced the best fit to the data in the experimentally relevant energy range was selected. In this case, the fits to the absolute value of the data were superior in the region between ∼1.0 eV – 1.5 eV analyzed in Figure 4, returning parameter values of $\hbar\Lambda = 4.00$ eV, $\hbar\Gamma = 748$ meV, $s = 4.08 \times 10^{-3}$ cm s^6 statV^{-1}, and $\chi_\infty^{(2)} = -9.97 \times 10^{-7}$ cm statV^{-1}.

The estimation of the dielectric function of the $LiNbO_3$ is described in the main text. We note here that the corresponding linear susceptibility model of $LiNbO_3$ to the second-order function described above is a Lorentz-model dielectric $\chi^{(1)}(\omega) = \chi_\infty^{(1)} + f/(\Lambda^2 - \omega^2 - \mathrm{i}\omega\Gamma)$. However, in the case where Λ is sufficiently detuned from the fundamental and second harmonic frequencies, $\chi^{(1)}(\omega)$ is well-approximated as a dielectric. In our case, $\hbar\Lambda - \hbar\Gamma > 2\omega_0$ such that the constant-dielectric approximation is appropriate and in agreement with Ref. 1.

S.2.2 Estimation of the Mode Oscillator Parameters

In this section, we use the dielectric models we have constructed to infer the parameters for oscillators models of the plasmon and Mie resonances of the coupled Au-$LiNbO_3$ nanostructures. Bare Au and $LiNbO_3$ particles are discussed in Sections S.2.2.1 and S.2.2.3, while the effects of weak coupling between members of the NP ensemble are discussed in Section S.2.2.2.

S.2.2.1 Bare Au NP Dipoles

The relation of the oscillator parameters of the Mie and plasmon resonances to the dielectric parameters of their respective particles is done via the particles' response functions. These are shown explicitly in Eqs. (S45)–(S49) (see Section S.2.4.2), and provide a direct link between the Au and $LiNbO_3$ dielectric functions $\epsilon_1(\omega)$ and ϵ_2, respectively, the resonance freqeuncies ω, damping rates γ, masses μ, and phase offsets ψ of each mode, and the physical observables of the particles.

For example, with $\epsilon_1(\omega)$ characterized in Section S.1.2.2, the response function of Eq. (S48) can be used used to construct the absorption cross section,

$$\sigma_1^{\mathrm{abs}}(\omega) = \frac{4\pi\omega}{c}\mathrm{Im}\left\{\frac{e^2}{2\omega_1\mu_1}\left(\frac{\mathrm{e}^{\mathrm{i}\psi_1}}{\Omega_1-\omega}+\frac{\mathrm{e}^{-\mathrm{i}\psi_1}}{\Omega_1^*+\omega}\right)+\sum_{i=1}^{2}\frac{e^2}{2\omega_{\mathrm{L}_i}\mu_{\mathrm{L}_i}}\left(\frac{\mathrm{e}^{\mathrm{i}\psi_{\mathrm{L}_i}}}{\Omega_{\mathrm{L}_i}-\omega}+\frac{\mathrm{e}^{-\mathrm{i}\psi_{\mathrm{L}_i}}}{\Omega_{\mathrm{L}_i}^*+\omega}\right)\right\} \tag{S8}$$

of each of the Au NP dipole modes, in which the plasmon oscillator parameters (subscript 1) and interband resonance parameters (subscript L_i) can be exactly expressed as functions of the dielectric parameters of $\epsilon_1(\omega)$. These functions are impossible to write explicitly, as the eigenfrequencies $\Omega_{1,\mathrm{L}_i} = \omega_{1,\mathrm{L}_i} - \mathrm{i}\gamma_{1,\mathrm{L}_i}/2$ are solutions to a sextic polynomial. Nevertheless, the oscillator parameters are simple to infer numerically by fitting Eq. (S8) to the standard form $\sigma_1^{\mathrm{abs}}(\omega) = (4\pi\omega/c)a_1^3\mathrm{Im}\{[\epsilon_1(\omega)-1]/[\epsilon_1(\omega)+2]\}$ as shown in Figure S12b with the NP radius a_1 set to 5 nm.

It is well-known that plasmon resonance energies are redshifted when the metal nanoparticle supporting them comes in close contact with a dielectric substrate with a large refractive index.[12] As the $LiNbO_3$ spheres in this investigation are much larger than the Au NPs and have a constant dielectric function larger than 5 (see Section S.2.2.3), some plasmon redshifting is expected. We incorporate the redshifts by lowering the Au NP dipole by 0.23 eV to align the NP absorption maximum with the observed maximum of hybrid-particle extinction measurements in Figure 3a. The oscillator parameters used in this work are given in Table S1.

However, to model the effects of radiation on the plasmon motion, one must modify the results extracted from Eq. (S8). More concretely, in the formal quasistatic approximation under which the absorption cross section is derived, only the rate of Au carrier losses to heat will appear in the cross section expression and any back-action of radiation on the dipole plasmon will be ignored. This back-action generally results in broadening of the plasmon lineshape, such that one can phenomenologically expand $\gamma_1 \to \gamma_1^{\mathrm{rad}} + \gamma_1^{\mathrm{NR}}$ to provide the plasmon damping rate with both a radiative (rad) and nonradiative (NR) component.

While approximate, this simple formulation of scattering loss works well in the limit $\gamma_1^{\mathrm{rad}} \ll \gamma_1^{\mathrm{NR}}$. The validity of the approximation can be demonstrated by comparing the Rayleigh scattering cross section

$$\begin{aligned}\sigma_1^{\mathrm{sca}}(\omega) = \frac{8\pi a_1^6 \omega^4}{3c^4} \Bigg| \frac{e^2}{2\omega_1 \mu_1} \left(\frac{\mathrm{e}^{\mathrm{i}\psi_1}}{\omega_1 - \mathrm{i}(\gamma_1^{\mathrm{NR}} + \gamma_1^{\mathrm{rad}})/2 - \omega} + \frac{\mathrm{e}^{-\mathrm{i}\psi_1}}{\omega_1 + \mathrm{i}(\gamma_1^{\mathrm{NR}} + \gamma_1^{\mathrm{rad}})/2 + \omega} \right) \\ + \sum_{i=1}^{2} \frac{e^2}{2\omega_{\mathrm{L}_i} \mu_{\mathrm{L}_i}} \left(\frac{\mathrm{e}^{\mathrm{i}\psi_{\mathrm{L}_i}}}{\omega_{\mathrm{L}_i} - \mathrm{i}(\gamma_{\mathrm{L}_i}^{\mathrm{NR}} + \gamma_{\mathrm{L}_i}^{\mathrm{rad}})/2 - \omega} + \frac{\mathrm{e}^{-\mathrm{i}\psi_{\mathrm{L}_i}}}{\omega_{\mathrm{L}_i} + \mathrm{i}(\gamma_{\mathrm{L}_i}^{\mathrm{NR}} + \gamma_{\mathrm{L}_i}^{\mathrm{rad}})/2 + \omega} \right) \Bigg|^2\end{aligned} \quad \text{(S9)}$$

in which the interband damping rates have been similarly transformed as $\gamma_{\mathrm{L}_i} \to \gamma_{\mathrm{L}_i}^{\mathrm{NR}} + \gamma_{\mathrm{L}_i}^{\mathrm{rad}}$ to account for radiation broadening and red shifting, to the exact Mie theory scattering cross section of a spherical Au NP with a 5-nm radius. Numerical fits of the modified oscillator model to the Mie scattering lineshape are generally unstable and suggest radiation-induced changes to both γ_1 and γ_{L_i} are roughly 1% or smaller, so we instead approximate the radiative damping rates of the dipole plasmons and interband resonances using their Larmor formulae. Assuming $\gamma_1^{\mathrm{NR}} \approx \gamma_1$, we have $\gamma_1^{\mathrm{rad}} = 2e^2\omega_1^2/3c^3\mu_1 = 1.01 \times 10^{-4}\gamma_1^{\mathrm{NR}}$. The radiation from the Lorentz oscillator resonances is similarly minimal, with $\gamma_{\mathrm{L}_1}^{\mathrm{rad}} = 7.15 \times 10^{-5}\gamma_{\mathrm{L}_1}^{\mathrm{NR}}$, $\gamma_{\mathrm{L}_1}^{\mathrm{rad}} = 3.67 \times 10^{-2}\gamma_{\mathrm{L}_2}^{\mathrm{NR}}$, and $\gamma_{\mathrm{L}_i}^{\mathrm{NR}} \approx \gamma_{\mathrm{L}_i}$. Figure S12a shows the excellent agreement between the Larmor-modified oscillator model and Mie scattering calculations.

S.2.2.2 Ensemble-Modified Au NP Dipoles

To describe in a concise way the scattering observables of the dipole plasmons in the NPs surrounding the $\mathrm{LiNbO_3}$ sphere, it is first necessary to quantify the alterations to the scattering behaviors of each NP caused by its neighbors. In other words, due to inter-NP interactions, we *cannot* take the masses μ_i (or, equivalently, the oscillator strengths $f_i = e^2/a_1^3\mu_i$) of the N NPs of the ensemble to be their bare values μ_1 (or $e^2/a_1^3\mu_1$).

We can begin this process by letting the polarizability of each NP be a tensor dependent on its position, such that differences between the ensemble perpendicular and parallel dipole oscillations of each NP can be captured. Explicitly, we let $\alpha_1(\omega) \to \boldsymbol{\alpha}_i(\omega) = \alpha_\perp(\omega)\hat{\mathbf{r}}_i\hat{\mathbf{r}}_i + \alpha_\parallel(\omega)(\hat{\boldsymbol{\theta}}_i\hat{\boldsymbol{\theta}}_i + \hat{\boldsymbol{\phi}}_i\hat{\boldsymbol{\phi}}_i)$, wherein the unit vectors $\hat{\mathbf{r}}_i = \hat{\mathbf{r}}(\theta_i, \phi_i)$, $\hat{\boldsymbol{\theta}}_i = \hat{\boldsymbol{\theta}}(\theta_i, \phi_i)$, and $\hat{\boldsymbol{\phi}}_i = \hat{\boldsymbol{\phi}}(\theta_i, \phi_i)$ are simply the spherical unit vectors evaluated at the angular position of the ith dipole. The tensor elements $\alpha_{\perp,\parallel}(\omega) = f_{\perp,\parallel}(a_1^3/2\omega_1)(\exp\{\mathrm{i}\psi_{\perp,\parallel}\}/[\Omega_1 - \omega] + \exp\{-\mathrm{i}\psi_{\perp,\parallel}\}/[\Omega_1^* + \omega])$ are the polarizabilities of the dipole components at $\mathbf{r}_i$ that are oriented perpendicular and parallel to the shell of Au NPs, respectively, and have magnitudes characterized by the oscillator strengths $f_{\perp,\parallel}$ and phases determined by the angles $\psi_{\perp,\parallel}$.

We can define the terms of $\boldsymbol{\alpha}_i(\omega)$ with poles at $\pm\Omega_1$ as

$$\boldsymbol{\alpha}_i^{(+)}(\omega) = \left(\frac{f_\perp a_1^3}{2\omega_1} \frac{\mathrm{e}^{\mathrm{i}\psi_\perp}}{\Omega_1 - \omega} \right) \hat{\mathbf{r}}_i\hat{\mathbf{r}}_i + \left(\frac{f_\parallel a_1^3}{2\omega_1} \frac{\mathrm{e}^{\mathrm{i}\psi_\parallel}}{\Omega_1 - \omega} \right) (\hat{\boldsymbol{\theta}}_i\hat{\boldsymbol{\theta}}_i + \hat{\boldsymbol{\phi}}_i\hat{\boldsymbol{\phi}}_i) \quad \text{(S10)}$$

and $\boldsymbol{\alpha}_i^{(-)}(\omega) = \boldsymbol{\alpha}_i^{(+)*}(-\omega)$ and further define the components of either as $\alpha_{\perp,\parallel}^{(\pm)}(\omega) = a_1^3 f_{\perp,\parallel}\mathrm{e}^{\mathrm{i}\psi_{\perp,\parallel}}/2\omega_1(\Omega_1 \mp \omega)$, respectively. Therefore, upon excitation by an impinging field $\mathbf{E}(\mathbf{r}_i, \omega)$, the dipole set up in the ith NP is $\mathbf{d}_i(\omega, \hat{\boldsymbol{\epsilon}}_i) = \boldsymbol{\alpha}_i(\omega) \cdot \mathbf{E}(\mathbf{r}_i, \omega) \approx E(\mathbf{r}_i, \omega)\Theta(\omega)\boldsymbol{\alpha}_i^{(+)}(\omega) \cdot$

$\hat{\boldsymbol{\epsilon}}_i + E(\mathbf{r}_i,\omega)\Theta(-\omega)\boldsymbol{\alpha}_i^{(-)}(\omega)\cdot\hat{\boldsymbol{\epsilon}}_i^*$. Here, $E(\mathbf{r}_i,\omega)$ is the (real) magnitude of the electric field at $\mathbf{r}_i$, $\hat{\boldsymbol{\epsilon}}_i$ is the complex polarization unit vector that describes the phases and orientations of the field components, and $\Theta(\omega)$ is the Heaviside function. Letting $\mathbf{d}^{(\pm)}(\omega,\hat{\boldsymbol{\epsilon}}_i)$ be the dipole terms valid at positive and negative frequencies, one finds the magnitude of $\mathbf{d}^{(+)}$ is

$$\left\|\mathbf{d}_i^{(+)}(\omega,\hat{\boldsymbol{\epsilon}}_i)\right\| \approx \frac{f_i(\hat{\boldsymbol{\epsilon}}_i)a_1^3}{2\omega_1}\frac{1}{|\Omega_1-\omega|}\Theta(\omega)E(\mathbf{r}_i,\omega) \tag{S11}$$

We can, therefore, define $f_i(\hat{\boldsymbol{\epsilon}}_i) = \sqrt{f_\perp^2|\hat{\mathbf{r}}_i\cdot\hat{\boldsymbol{\epsilon}}_i|^2 + f_\parallel^2(|\hat{\boldsymbol{\theta}}_i\cdot\hat{\boldsymbol{\epsilon}}_i|^2 + |\hat{\boldsymbol{\phi}}_i\cdot\hat{\boldsymbol{\epsilon}}_i|^2)}$ as the orientation-dependent dipole plasmon oscillator strength.

With $\hat{\boldsymbol{\epsilon}}_i$ oriented strictly perpendicular to the ensemble, one can see that $f_i(\hat{\mathbf{r}}_i) = f_\perp$. Similarly, $f_i(\hat{\boldsymbol{\theta}}_i) = f_\parallel$. Figure S15c shows the simulated absorption cross section maxima of NPs in ensembles excited with these two choices of $\hat{\boldsymbol{\epsilon}}_i$, where it can be seen that for $N \gtrsim 50$ the absorption cross section of each particle is independent of N. Analytically, the absorption cross sections of ensemble perpendicular and parallel dipoles are simply $\sigma_{\perp,\parallel}^{\rm abs}(\omega) = (4\pi\omega/c)R_{\rm abs}{\rm Im}\{\alpha_{\perp,\parallel}(\omega)\}$, where $R_{\rm abs} = 1.16$ phenomenologically accounts for the absorption contribution of the Lorentz oscillators in the region $\omega < 2.8$ eV. With $\cos\psi_{\perp,\parallel} \approx \cos\psi_1 \approx 1$, the cross section maxima are then $\sigma_{\perp,\parallel}^{\rm abs}(\omega_1) \approx 4\pi R_{\rm abs} f_{\perp,\parallel} a_1^3/c\gamma_1$ such that the oscillator strengths implied by Figure S14c are $f_\parallel = 2.37\times10^{30}$ s^{-2} and $f_\perp/f_\parallel = 0.668$.

Finally, to define the orientation-averaged plasmon oscillator strength f_1 used in the main text, we let an electric field $\mathbf{E}_0(\mathbf{r},\omega) = E_0\pi[\exp(2{\rm i}k_0x)\delta(\omega-2\omega_0) + \exp(-2{\rm i}k_0x)\delta(\omega+2\omega_0)]\hat{\mathbf{z}}$ excite the NP ensemble, producing $f_i(\hat{\mathbf{z}}) = \sqrt{f_\perp^2\cos^2\theta_i + f_\parallel^2\sin^2\theta_i}$. We then take a formal average

$$\langle f_i(\hat{\mathbf{z}})\rangle_i = \frac{\int_0^{2\pi}\int_0^{\pi} f_i(\hat{\mathbf{z}})(t)\sin\theta_i\,{\rm d}\theta_i{\rm d}\phi_i}{\int_0^{2\pi}\int_0^{\pi}\sin\theta_i\,{\rm d}\theta_i{\rm d}\phi_i} \tag{S12}$$

over all of the possible $\hat{\mathbf{r}}_i$ and define $f_1 = \langle f_i(\hat{\mathbf{z}})\rangle_i$, giving

$$f_1 = \frac{1}{2}\left(f_\perp + \frac{f_\parallel^2\cos^{-1}\left\{\frac{f_\perp}{f_\parallel}\right\}}{\sqrt{f_\parallel^2 - f_\perp^2}}\right) = 2.13\times10^{30}\ \text{s}^{-2} \tag{S13}$$

S.2.2.3 Bare Lithium Niobate Mie Resonances

Inference of the oscillator parameters of the Mie resonances $\boldsymbol{\beta}$ of the $LiNbO_3$ microsphere is more straightforward, as their damping rates $\gamma_{\boldsymbol{\beta}}$ contain only losses to radiation. Thus, only a single observable is needed to extract the full set of parameters from each mode. We use the response functions shown in Eq. (S49) to fit $\gamma_{\boldsymbol{\beta}}$ as well as the resonance frequencies $\omega_{\boldsymbol{\beta}}$, effective masses $\mu_{\boldsymbol{\beta}}$ (equivalently, the oscillator strenghts $f_{\boldsymbol{\beta}} = e^2/a_2^3\mu_{\boldsymbol{\beta}}$), and phase offsets $\psi_{\boldsymbol{\beta}}$. Explicitly, we let

$$\begin{aligned}
(\sqrt{\epsilon_2})^\ell A_{p\ell m}^<(\omega) - 1 &\approx -\frac{e^2}{a_2^3}\frac{1}{2\omega_{M\ell}\mu_{M\ell}}\left(\frac{{\rm e}^{{\rm i}\psi_{M\ell}}}{\Omega_{M\ell}-\omega} + \frac{{\rm e}^{-{\rm i}\psi_{M\ell}}}{\Omega_{M\ell}^*+\omega}\right)\\
(\sqrt{\epsilon_2})^\ell B_{p\ell m}^<(\omega) - 1 &\approx -\frac{e^2}{a_2^3}\frac{1}{2\omega_{E\ell}\mu_{E\ell}}\left(\frac{{\rm e}^{{\rm i}\psi_{E\ell}}}{\Omega_{E\ell}-\omega} + \frac{{\rm e}^{-{\rm i}\psi_{E\ell}}}{\Omega_{E\ell}^*+\omega}\right)
\end{aligned} \tag{S14}$$

Note that, in contrast with Eq. (S49), the above expressions only consider a single resonance for a given $\boldsymbol{\beta}$. See Section S.2.4 for details. The results of the fits are shown graphically in Figure S15b,c and are tabulated in Table S2.

S.2.3 Derivation of the Scattering Observables

We provide here a more thorough derivation of the superradiant scattering enhancement ratio. First, in Section S.2.3.1, we describe the scattered fields and power of the modes of a dielectric sphere with a diameter on the order of a wavelength of the scattered light. Second, in sections Section S.2.3.2 we develop the scattering observables of the ensemble of NPs surrounding a dielectric sphere. In Section S.2.3.3, we complete the calculation of the ratio of scattered powers of a NP-dressed and bare $LiNbO_3$ dielectric sphere.

S.2.3.1 Mie Modes of the Lithium Niobate Sphere Driven by a Plane Wave

The time averaged radiated power from each of the $LiNbO_3$ SH modes can be calculated from Poynting's theorem. Beginning with

$$\boldsymbol{\mathcal{E}}_{\mathrm{sca}}(\mathbf{r},\omega) = \sum_{\boldsymbol{\beta}} \frac{1}{a_2^{\ell+2}} \left(\rho_{\boldsymbol{\beta}}(\omega)\mathbf{X}_{\boldsymbol{\beta}}(\mathbf{r},k_{\boldsymbol{\beta}}) + \rho^*_{\boldsymbol{\beta}}(-\omega)\mathbf{X}^*_{\boldsymbol{\beta}}(\mathbf{r},k_{\boldsymbol{\beta}})\right) \tag{S15}$$

wherein (2) signifies that the fields arise from a second-order scattering process, $\rho_{\boldsymbol{\beta}}(\omega)$ are the magnitudes of the moments of the modes $\boldsymbol{\beta}$, and $\mathbf{X}_{\boldsymbol{\beta}}(\mathbf{r},\omega)$ are the regularized vector spherical harmonics

$$\begin{aligned}
\mathbf{M}_{p\ell m}(\mathbf{r},k) = &\sqrt{(2-\delta_{m0})\frac{2\ell+1}{\ell(\ell+1)}\frac{(\ell-m)!}{(\ell+m)!}} \left[\frac{(-1)^{p+1}m}{\sin\theta} h_\ell^{(1)}(kr)P_{\ell m}(\cos\theta)S_{p+1}(m\phi)\hat{\boldsymbol{\theta}}\right.\\
&\left. -h_\ell^{(1)}(kr)\frac{\partial P_{\ell m}(\cos\theta)}{\partial\theta}S_p(m\phi)\hat{\boldsymbol{\phi}}\right]\\
\mathbf{N}_{p\ell m}(\mathbf{r},k) = &\sqrt{(2-\delta_{m0})\frac{2\ell+1}{\ell(\ell+1)}\frac{(\ell-m)!}{(\ell+m)!}} \left[\frac{\ell(\ell+1)}{kr} h_\ell^{(1)}(kr)P_{\ell m}(\cos\theta)S_p(m\phi)\hat{\mathbf{r}}\right.\\
&\left. +\frac{1}{kr}\frac{\partial\{rh_\ell^{(1)}(kr)\}}{\partial r}\left(\frac{\partial P_{\ell m}(\cos\theta)}{\partial\theta}S_p(m\phi)\hat{\boldsymbol{\theta}} + \frac{(-1)^{p+1}m}{\sin\theta}P_{\ell m}(\cos\theta)S_{p+1}(m\phi)\hat{\boldsymbol{\phi}}\right)\right]
\end{aligned} \tag{S16}$$

for $T = M$ and E, respectively, the calculation of the Poynting vector is straightforward. Here, $h_\ell^{(1)}(x)$ are the spherical Hankel functions of the first kind, $P_{\ell m}(x)$ are the associated Legendre polynomials, $S_p(x) = \cos(x)\delta_{p\,\mathrm{even}} + \sin(x)\delta_{p\,\mathrm{odd}}$, and $k = \omega/c$. Further, with

$$\boldsymbol{\mathcal{B}}_{\mathrm{sca}}(\mathbf{r},\omega) = \sum_{\boldsymbol{\beta}} \frac{c}{\mathrm{i}\omega a_2^{\ell+2}} \left[\rho_{\boldsymbol{\beta}}(\omega)\nabla\times\mathbf{X}_{\boldsymbol{\beta}}(\mathbf{r},k_{\boldsymbol{\beta}}) + \rho^*_{\boldsymbol{\beta}}(-\omega)\nabla\times\mathbf{X}^*_{\boldsymbol{\beta}}(\mathbf{r},k_{\boldsymbol{\beta}})\right] \tag{S17}$$

from Faraday's law,

$$\lim_{r\to\infty}\int_0^{2\pi}\int_0^{\pi}\mathbf{X}_{\boldsymbol{\beta}}(\mathbf{r},k_{\boldsymbol{\beta}})\times\nabla\times\mathbf{X}^*_{\boldsymbol{\beta}'}(\mathbf{r},k_{\boldsymbol{\beta}'})\cdot\hat{\mathbf{r}}r^2\sin\theta\,\mathrm{d}\theta\,\mathrm{d}\phi = 4\pi(1-\delta_{p1}\delta_{m0})\frac{(-1)^{\ell+1}\mathrm{i}^{2\ell+1}}{k_{\boldsymbol{\beta}}}\delta_{\boldsymbol{\beta}\boldsymbol{\beta}'} \tag{S18}$$

and

$$\frac{\omega_0}{\pi}\int_{-\pi/2\omega_0}^{\pi/2\omega_0}\mathrm{e}^{\mathrm{i}(\omega-\omega')t}\,\mathrm{d}t = \frac{2\omega_0}{\pi}\frac{\sin\left(\frac{\pi}{2\omega_0}[\omega-\omega']\right)}{\omega-\omega'} \tag{S19}$$

the time-averaged scattered power from the dielectric sphere can be rapidly simplified from

$$\begin{aligned}\bar{P}_2(2\omega_0) &\equiv \langle P_{\boldsymbol{\beta}}(t)\rangle_{2\pi/2\omega_0} \\ &= \frac{2\omega_0}{2\pi}\frac{c}{4\pi}\int_{-\pi/2\omega_0}^{\pi/2\omega_0}\oint\boldsymbol{\mathcal{E}}_{\mathrm{sca}}(\mathbf{r},t)\times\boldsymbol{\mathcal{B}}_{\mathrm{sca}}(\mathbf{r},t)\cdot\mathrm{d}\mathbf{a}\,\mathrm{d}t \\ &= \lim_{r\to\infty}\frac{\omega_0 c}{2\pi^2}\int_0^{2\pi}\int_0^{\pi}\iint\frac{\sin\left(\frac{\pi}{2\omega_0}[\omega-\omega']\right)}{\omega-\omega'}\boldsymbol{\mathcal{E}}_{\mathrm{sca}}(\mathbf{r},\omega)\times\boldsymbol{\mathcal{B}}^*_{\mathrm{sca}}(\mathbf{r},\omega')r^2\sin\theta\cdot\hat{\mathbf{r}}\;\frac{\mathrm{d}\omega\,\mathrm{d}\omega'}{4\pi^2}\,\mathrm{d}\theta\,\mathrm{d}\phi\end{aligned} \tag{S20}$$

wherein the surface integral is taken to be across a sphere of radius $r\to\infty$, $\langle\cdot\rangle_\tau$ is the time-average operator over a period τ, and the Fourier transforms of the fields $\boldsymbol{\mathcal{F}}_{\mathrm{sca}}(\mathbf{r},t) = \int\boldsymbol{\mathcal{F}}_{\mathrm{sca}}(\mathbf{r},\omega)\exp(-\mathrm{i}\omega t)\,\mathrm{d}\omega/2\pi$ have been used. Letting the moments of the sphere be driven by the second-harmonic upconversion of an incoming plane wave of frequency ω and electric field strength E_0, we can say $\rho_{\boldsymbol{\beta}}(\omega) = C_{\boldsymbol{\beta}}(\omega)\alpha_{\boldsymbol{\beta}}(\omega)E_0\pi[\delta(\omega-2\omega_0)+\delta(\omega+2\omega_0)]$ with $\alpha_{\boldsymbol{\beta}}(\omega) = (a_2^{\ell+2}f_{\boldsymbol{\beta}}/2\omega_{\boldsymbol{\beta}})\exp(i\psi_{\boldsymbol{\beta}})/(\omega_{\boldsymbol{\beta}}-i\gamma_{\boldsymbol{\beta}}/2-\omega)$ the linear polarizability of the $\boldsymbol{\beta}^{\mathrm{th}}$ mode and $C_{\boldsymbol{\beta}}(\omega)$ an overlap coefficient defined in Eq. (S56). The final result is

$$\bar{P}_2(2\omega_0) = \sum_{\boldsymbol{\beta}}(1-\delta_{p1}\delta_{m0})\frac{c^2E_0^2}{4\pi\omega_0 k_{\boldsymbol{\beta}}a_2^{2\ell+4}}|C_{\boldsymbol{\beta}}(2\omega_0)|^2|\alpha_{\boldsymbol{\beta}}(2\omega_0)|^2 \tag{S21}$$

Importantly, the radiated power from the Mie modes contains no cross-terms due to the orthogonality condition imposed by Eq. (S18). Further, due to the good agreement between an oscillator model of each mode and its electromagnetic response (see Section S.2.2), the scattered power can also be modeled mechanically. To do so, it is important to first define a generalized coordinate $q_{\boldsymbol{\beta}}(\omega)$ to represent the displacement magnitude of the moments $\rho_{\boldsymbol{\beta}}(\omega)$ that obeys the reality condition $q_{\boldsymbol{\beta}}(-\omega) = q^*_{\boldsymbol{\beta}}(\omega)$. We will choose the definition $q_{\boldsymbol{\beta}}(\omega) = [\rho_{\boldsymbol{\beta}}(\omega)+\rho^*_{\boldsymbol{\beta}}(-\omega)]/2ea_2^{\ell-1}$ such that $q_{\boldsymbol{\beta}}(t) = \mathrm{Re}\{\rho_{\boldsymbol{\beta}}(t)\}/ea_2^{\ell-1}$ is a real quantity.

With $q_{\boldsymbol{\beta}}(t)$ defined, we model losses to radiation as a weak, frequency-independent linear

damping process. In this case,

$$\begin{aligned} P_2(t) &= -\sum_{\boldsymbol{\beta}} \dot{q}_{\boldsymbol{\beta}}(t) F_{\boldsymbol{\beta}}^{\mathrm{rad}}(t) \\ &= \sum_{\boldsymbol{\beta}} A_{\boldsymbol{\beta}} \mu_{\boldsymbol{\beta}} \gamma_{\boldsymbol{\beta}} \dot{q}_{\boldsymbol{\beta}}^2(t) \end{aligned} \tag{S22}$$

where $A_{\boldsymbol{\beta}}$ is a unitless proportionality constant that relates the mechanical quantities to the electromagnetics. This leads directly to

$$\bar{P}_2(2\omega_0) = \sum_{\boldsymbol{\beta}} A_{\boldsymbol{\beta}} \gamma_{\boldsymbol{\beta}} \mu_{\boldsymbol{\beta}} \frac{\omega_0^2 E_0^2}{2e^2 a_2^{2\ell-2}} |C_{\boldsymbol{\beta}}(2\omega_0)|^2 |\alpha_{\boldsymbol{\beta}}(2\omega_0)|^2 \tag{S23}$$

via Eq. (S19) and the identity $q_{\boldsymbol{\beta}}(t) = \int(-\mathrm{i}\omega)\exp(-\mathrm{i}\omega t) q_{\boldsymbol{\beta}}(\omega)\ \mathrm{d}\omega/2\pi$ such that $A_{\boldsymbol{\beta}} = (1-\delta_{p1}\delta_{m0})(e^2c^3/2\pi\omega_0^3 a_2^6 \mu_{\boldsymbol{\beta}}\omega_{\boldsymbol{\beta}}\gamma_{\boldsymbol{\beta}}) = (1-\delta_{p1}\delta_{m0})(f_{\boldsymbol{\beta}}c^3/2\pi\omega_0^3 a_2^3 \omega_{\boldsymbol{\beta}}\gamma_{\boldsymbol{\beta}})$. Finally, to arrive at Eq. (S1) and the notation of the main text we substitute the explicit form of $A_{\boldsymbol{\beta}}$ and make the simplification $\bar{P}_2(2\omega_0) \to P^{(1)}(2\omega_0)$ to the notation.

S.2.3.2 Plasmon Dipoles Driven by the Scattered Lithium Niobate Electric Field

The radiated power by the ensemble of Au NPs can be straightforwardly derived using a mechanical model parameterized using the values outlined in Sections S.2.2.1 and S.2.2.2. More explicitly, the time-averaged scattered power by the ith dipole plasmon of the Au NP ensemble when the collection is driven by an external field can be calculated in a straightforward manner using a well-known mechanical model of dipole radiation

$$P_i(t) = -\dot{\mathbf{x}}_i(t) \cdot A_i(N, \hat{\boldsymbol{\epsilon}}_i) \mathbf{F}_i^{\mathrm{rad}}(t) \tag{S24}$$

wherein $\mathbf{x}_i(t) = \mathbf{d}_i(t)/e$ is the coordinate characterizing the magnitude of the dipole plasmon located at $\mathbf{r}_i$ and oriented along $\hat{\mathbf{x}}_i$ and $A_i(N, \hat{\boldsymbol{\epsilon}}_i)$ is a phenomenological enhancement factor that builds in the ensemble-induced scattering enhancements seen in Figure 4c. Similarly to the orientation-dependent oscillator strengths, we take $A_i(N, \hat{\boldsymbol{\epsilon}}_i) = \sqrt{A_\perp^2(N)|\hat{\mathbf{r}}_i \cdot \hat{\boldsymbol{\epsilon}}_i|^2 + A_\parallel^2(N)(|\hat{\boldsymbol{\theta}}_i \cdot \hat{\boldsymbol{\epsilon}}_i|^2 + |\hat{\boldsymbol{\phi}}_i \cdot \hat{\boldsymbol{\epsilon}}_i|^2)}$ where $A_{\perp,\parallel}(N)$ are the enhancements experience by ensemble-perpendicular and parallel dipoles, respectively, in an ensemble of N NPs.

With the radiation back-force $\mathbf{F}_i^{\mathrm{rad}}(t)$ treated as a damping force, one can let $\mathbf{F}_i^{\mathrm{rad}}(t) = -\mu_i(\hat{\boldsymbol{\epsilon}}_i)\gamma_1^{\mathrm{rad}}\dot{\mathbf{x}}_i(t)$, with $\mu_i(\hat{\boldsymbol{\epsilon}}_i) = e^2/a_1^3 f_i(\hat{\boldsymbol{\epsilon}}_i)$ the mass of the ith plasmon, such that an average over a period $\tau = 2\pi/2\omega_0$ of the second harmonic frequency gives:

$$\begin{aligned} \bar{P}_i(2\omega_0) &\equiv \langle P_i(t) \rangle_{2\pi/2\omega_0} \\ &= A_i(N, \hat{\boldsymbol{\epsilon}}_i) \frac{\omega_0}{\pi} \int_{-\pi/2\omega_0}^{\pi/2\omega_0} \mu_i(\hat{\boldsymbol{\epsilon}}_i) \gamma_1^{\mathrm{rad}} \dot{x}_i^2(t)\ \mathrm{d}t \end{aligned} \tag{S25}$$

Inserting the identity $\dot{x}_i(t) = \int(-\mathrm{i}\omega)x_i(\omega)\exp(\mathrm{i}\omega t)\,\mathrm{d}\omega/2\pi$ twice and letting $\mathbf{x}_i(\omega) = \boldsymbol{\alpha}_i(\omega)\cdot\mathbf{E}^{(2)}_{\mathrm{sca}}(\mathbf{r},\omega)/e$, one finds

$$\bar{P}_i(2\omega_0) = A_i(N,\hat{\boldsymbol{\epsilon}}_i)\frac{\omega_0\gamma_1^{\mathrm{rad}}}{4\pi^3 a_1^3 f_i(\hat{\boldsymbol{\epsilon}}_i)}\iint\int_{-\pi/2\omega_0}^{\pi/2\omega_0}\omega\omega'\left[\boldsymbol{\alpha_i}(\omega)\cdot\mathbf{E}^{(2)}_{\mathrm{sca}}(\mathbf{r}_i,\omega)\right]\cdot\left[\boldsymbol{\alpha_i^*}(\omega')\cdot\mathbf{E}^{(2)*}_{\mathrm{sca}}(\mathbf{r}_i,\omega')\right]$$
$$\times\,\mathrm{e}^{-\mathrm{i}(\omega-\omega')t}\,\mathrm{d}t\,\mathrm{d}\omega\,\mathrm{d}\omega' \tag{S26}$$

Application of the identity of Eq. (S19) and neglect of terms proportional to $\rho_{\boldsymbol{\beta}}(-2\omega_0) \ll \rho_{\boldsymbol{\beta}}(2\omega_0)$ provides

$$\begin{aligned}\bar{P}_i(2\omega_0) \approx\ & A_i(N,\hat{\boldsymbol{\epsilon}}_i)\frac{2\gamma_1^{\mathrm{rad}}}{a_1^3 f_i(\hat{\boldsymbol{\epsilon}}_i)}\sum_{\boldsymbol{\beta}\boldsymbol{\beta}'}E_0^2\omega_0^2 a_2^{-\ell-\ell'-4}C_{\boldsymbol{\beta}}(2\omega_0)C^*_{\boldsymbol{\beta}'}(2\omega_0)\alpha_{\boldsymbol{\beta}}(2\omega_0)\alpha^*_{\boldsymbol{\beta}'}(2\omega_0)\\ &\times\left[\boldsymbol{\alpha}_i(2\omega_0)\cdot\mathbf{X}_{\boldsymbol{\beta}}(\mathbf{r}_i,k_{\boldsymbol{\beta}})\right]\cdot\left[\boldsymbol{\alpha}_i(2\omega_0)\cdot\mathbf{X}_{\boldsymbol{\beta}'}(\mathbf{r}_i,k_{\boldsymbol{\beta}'})\right]^*\end{aligned} \tag{S27}$$

Finally, we average over the power scattered from each dipole i to define the power scattered from the typical dipole. Numerically, one can show that terms proportional to $\langle A_i(N,\hat{\boldsymbol{\epsilon}}_i)\left[\boldsymbol{\alpha}_i(2\omega_0)\cdot\mathbf{X}_{\boldsymbol{\beta}}(\mathbf{r}_i,k_{\boldsymbol{\beta}})\right]\cdot\left[\boldsymbol{\alpha}_i(2\omega_0)\cdot\mathbf{X}_{\boldsymbol{\beta}'}(\mathbf{r}_i,k_{\boldsymbol{\beta}'})\right]^*/f_i(\hat{\boldsymbol{\epsilon}}_i)\rangle_i$ are at least two orders of magnitude smaller when $\boldsymbol{\beta}\neq\boldsymbol{\beta}'$ than when $\boldsymbol{\beta}=\boldsymbol{\beta}'$. Thus, we can safely neglect the cross terms of the sum and define $\bar{P}_1(\omega) = \langle\bar{P}_i(\omega)\rangle_i$ such that

$$\bar{P}_1(2\omega_0) \approx \frac{2\gamma_1^{\mathrm{rad}}}{a_1^3}E_0^2\omega_0^2\sum_{\boldsymbol{\beta}}\frac{|C_{\boldsymbol{\beta}}(2\omega_0)|^2}{a_2^{2\ell+4}}|\alpha_{\boldsymbol{\beta}}(2\omega_0)|^2\left\langle A_i(N,\hat{\boldsymbol{\epsilon}}_i)\frac{\|\boldsymbol{\alpha}_i(2\omega_0)\cdot\mathbf{X}_{\boldsymbol{\beta}}(\mathbf{r}_i,k_{\boldsymbol{\beta}})\|^2}{f_i(\hat{\boldsymbol{\epsilon}}_i)}\right\rangle_i \tag{S28}$$

Rectification of Eq. (S28) with Eq. (S2) and the notation of the main text can be achieved by substituting appropriately the dimensionless constant

$$\begin{aligned}K_{\boldsymbol{\beta}} &= \frac{\omega_1^2\gamma_1^2}{A(N)f_1 a_1^6}\left\langle\frac{A_i(N,\hat{\boldsymbol{\epsilon}}_i)\|\boldsymbol{\alpha}_i(2\omega_0)\cdot\mathbf{X}_{\boldsymbol{\beta}}(\mathbf{r}_i,k_{\boldsymbol{\beta}})\|^2}{f_i(\hat{\boldsymbol{\epsilon}}_i)}\right\rangle_i\\ &\approx\left\langle\frac{A_i(N,\hat{\boldsymbol{\epsilon}}_i)}{A(N)}\frac{f_\perp^2|\mathbf{X}_{\boldsymbol{\beta}}(\mathbf{r}_i,k_{\boldsymbol{\beta}})\cdot\hat{\mathbf{r}}_i|^2 + f_\parallel^2\left(|\mathbf{X}_{\boldsymbol{\beta}}(\mathbf{r}_i,k_{\boldsymbol{\beta}})\cdot\hat{\boldsymbol{\theta}}_i|^2 + |\mathbf{X}_{\boldsymbol{\beta}}(\mathbf{r}_i,k_{\boldsymbol{\beta}})\cdot\hat{\boldsymbol{\phi}}_i|^2\right)}{f_1 f_i(\hat{\boldsymbol{\epsilon}}_i)}\right\rangle_i\end{aligned} \tag{S29}$$

and simplifying the notation such that $\bar{P}_1(2\omega_0)\to P_{\mathrm{pl}}(2\omega_0)$. Here, we have taken

$$A(N) = \frac{1}{2}\left(A_\perp(N) + \frac{A_\parallel^2(N)\cos^{-1}\left\{\frac{A_\perp(N)}{A_\parallel(N)}\right\}}{\sqrt{A_\parallel^2(N)-A_\perp^2(N)}}\right) \tag{S30}$$

similar to the definition of the angle-averaged oscillator strength f_1.

Thus, all that is left to characterize in $\bar{P}_1(2\omega_0)$ are the phenomenological constants $A_{\perp,\parallel}(N)$. This can be achieved by replacing $\boldsymbol{\mathcal{E}}_{\mathrm{sca}}(\mathbf{r}_i,\omega)$ with a field $\mathbf{E}_0(\mathbf{r}_i,\omega) = E_0\pi[\delta(\omega-2\omega_0)+\delta(\omega+2\omega_0)]\hat{\mathbf{e}}_{\perp,\parallel}$ with $\hat{\mathbf{e}}_{\perp,\parallel} = \hat{\mathbf{r}}_i$ and $\hat{\boldsymbol{\theta}}_i$, respectively, in Eq. (S26). The results can then

be compared to the simulations detailed in Figure 4c, which provide the simulated scattering cross sections of a grid of spheres upon which a plane wave polarized parallel or perpendicular to the ensemble plane is impinged, such that only ensemble-parallel or perpendicular dipoles are excited. The use of $\mathbf{E}_0(\mathbf{r}_i, \omega)$ in the theory faithfully approximates the simulated arrangement in the limit where the spacing between NPs is much smaller than the impinging light wavelength and the $LiNbO_3$ sphere radius a_2.

Explicitly, the power scattered by the ith dipole is

$$\bar{P}_i^{\text{PW}}(2\omega_0, \hat{\mathbf{e}}_{\perp,\parallel}) = A_{\perp,\parallel}(N)\frac{2\gamma_1^{\text{rad}}}{a_1^3 f_{\perp,\parallel}} E_0^2\omega_0^2 \left\|\boldsymbol{\alpha}_i(2\omega_0)\cdot\hat{\mathbf{e}}_{\perp,\parallel}\right\|^2 \tag{S31}$$

and, after an angular average,

$$\bar{P}_1^{\text{PW}}(2\omega_0, \hat{\mathbf{e}}_{\perp,\parallel}) \approx A(N) f_{\perp,\parallel}\frac{a_1^3\gamma_1^{\text{rad}}}{2\omega_1^2}\frac{E_0^2\omega_0^2}{|\Omega_1 - 2\omega_0|^2} \tag{S32}$$

where the superscript PW signifies that the driving source in a plane wave and $\bar{P}_1^{\text{PW}}(2\omega_0) = \langle \bar{P}_i^{\text{PW}}(2\omega_0)\rangle_i$. The associated scattering cross section to $\bar{P}_1^{\text{PW}}(2\omega_0)$ that can be directly compared to the simulation is given by

$$\sigma_{\perp,\parallel}^{\text{sca}}(N, 2\omega_0) = R_{\text{sca}} A_{\perp,\parallel}(N) f_{\perp,\parallel}\frac{4\pi a_1^3\gamma_1^{\text{rad}}}{c\omega_1^2}\frac{\omega_0^2}{|\Omega_1 - 2\omega_0|^2} \tag{S33}$$

wherein $R_{\text{sca}} = 6.00$ is a scaling constant that builds in the contribution of the Lorentz resonances of Au at low energies. With all other constants already well-characterized, we find the best fits to the simulated scattering data are $A_\perp(N) = 2.71N^{0.489}$ and $A_\parallel(N) = 2.43N^{0.730}$ such that $A(1000) = 301$. Finally, we find that due to the similar average magnitudes over $\mathbf{r}_i$ of the harmonics $\mathbf{X}_{\boldsymbol{\beta}}(\mathbf{r}_i, k_{\boldsymbol{\beta}})$, $K_{\boldsymbol{\beta}}$ varies little between modes, with $\max_{\boldsymbol{\beta}}\{K_{\boldsymbol{\beta}}\} = K_{M,0,10,2} = 10.2 \times 10^{-3}$ and $\min_{\boldsymbol{\beta}}\{K_{\boldsymbol{\beta}}\} = K_{E,0,10,0} = 5.04 \times 10^{-3}$.

S.2.3.3 Ratio of Time-Averaged Scattered Powers

One can see from Eqs. (S1) and (S2) that the superradiant scattering enhancement ratio $N\bar{P}_1(2\omega_0)/\bar{P}_2(2\omega_0)$ is greatly simplified by defining the spectral profile the associated spectral profile

$$\kappa(\omega) = \frac{\sum_{\boldsymbol{\beta}} K_{\boldsymbol{\beta}}|C_{\boldsymbol{\beta}}(\omega)|^2|\alpha_{\boldsymbol{\beta}}(\omega)|^2/a_2^{2\ell}}{\sum_{\boldsymbol{\beta}} f_2|C_{\boldsymbol{\beta}}(\omega)|^2|\alpha_{\boldsymbol{\beta}}(\omega)|^2/\gamma_2\omega_{\boldsymbol{\beta}}a_2^{2\ell}} \tag{S34}$$

wherein $f_2 = \langle f_{\boldsymbol{\beta}}\rangle_{\boldsymbol{\beta}}$ and $\gamma_2 = \langle \gamma_{\boldsymbol{\beta}}\rangle_{\boldsymbol{\beta}}$ are the average Mie oscillator strength and damping rate, respectively. As $K_{\boldsymbol{\beta}}$ is small for all of the relevant modes of the system, so too is $\kappa(\omega)$ for ω in the optical region, with a value between 1.67×10^{-3} and 1.78×10^{-3}.

S.2.4 Derivation of the Near-Field Enhanced Scattering

In this section, the derivation of the coupled-oscillator model of the hybrid Au-$LiNbO_3$ nanostructures is developed from first principles. Section S.2.4.1 details the calculations of the relevant field quantities and Section S.2.4.2 translates these field quantities into an osicllator model.

S.2.4.1 Nonlinear Optics First Principles

The oscillator model of the modes of the $LiNbO_3$ microsphere is generated from the solutions to the coupled nonlinear wave equation for the total electric field

$$\nabla \times \nabla \times \mathbf{E}(\mathbf{r},\omega) - \frac{\omega^2}{c^2}\mathbf{E}(\mathbf{r},\omega) - \frac{\omega^2}{c^2}\left[\mathbf{P}^{(1)}(\mathbf{r},\omega) + \mathbf{P}^{(2)}(\mathbf{r},\omega)\right] = \frac{4\pi \mathrm{i}\omega}{c^2}\mathbf{J}_0(\mathbf{r},\omega) \tag{S35}$$

wherein $\mathbf{J}_0(\mathbf{r},\omega)$ is the current density of the laser. The polarization fields $\mathbf{P}^{(1)}(\mathbf{r},\omega) = \chi_2^{(1)}(\mathbf{r})\mathbf{E}(\mathbf{r},\omega)$ and

$$\mathbf{P}^{(2)}(\mathbf{r},\omega) = \int_{-\infty}^{\infty} \mathbf{E}(\mathbf{r},\omega-\omega')\cdot\boldsymbol{\chi}_2^{(2)}(\mathbf{r};\omega',\omega-\omega')\cdot\mathbf{E}(\mathbf{r},\omega')\,\frac{\mathrm{d}\omega'}{2\pi} \tag{S36}$$

build in the first- and second-order material response of the $LiNbO_3$ sphere, respectively. The sphere's electric susceptibilities are allowed to vary in space such that $\chi_2^{(1)}(\mathbf{r}) = \chi_2^{(1)}\Theta(r \le a_2)$ and $\boldsymbol{\chi}_2^{(2)}(\mathbf{r};\omega',\omega-\omega') = \mathbf{1}_3\chi_2^{(2)}(\omega',\omega-\omega')\Theta(r \le a_2)$ with $\mathbf{1}_3$ the rank-3 identity tensor. The sphere's dielectric function is then given by $\epsilon_2(\mathbf{r}) = 1 + 4\pi\chi_2^{(1)}(\mathbf{r})$.

From here, the electric field can be perturbatively expanded with the assumption that $|\boldsymbol{\chi}_2^{(2)}(\mathbf{r};\omega',\omega-\omega')|$ is a small quantity. Explicitly, we let $\mathbf{E}(\mathbf{r},\omega) = \mathbf{E}_0(\mathbf{r},\omega) + \sum_{n=2}^{\infty}\mathbf{E}_0^{(n)}(\mathbf{r},\omega) + \sum_{n=1}^{\infty}\mathbf{E}_{\mathrm{sca}}^{(n)}(\mathbf{r},\omega)$, wherein the first term is the laser's electric field and the terms in the sums contribute n^{th}-order corrections to the vacuum-like and scattered fields $\sum_{n=2}^{\infty}\mathbf{E}_0^{(n)}(\mathbf{r},\omega)$ and $\mathbf{E}_{\mathrm{sca}}(\mathbf{r},\omega) = \sum_{n=1}^{\infty}\mathbf{E}_{\mathrm{sca}}^{(n)}(\mathbf{r},\omega)$, respectively, that are set up by the polarized sphere. Subtracting the laser field equation $\nabla \times \nabla \times \mathbf{E}_0(\mathbf{r},\omega) - (\omega^2/c^2)\mathbf{E}_0(\mathbf{r},\omega) = (4\pi\mathrm{i}\omega/c^2)\mathbf{J}_0(\mathbf{r},\omega)$ from Eq. (S35), one finds

$$\begin{aligned}\left(\{\nabla\times\nabla\times\} - \epsilon_2(\mathbf{r})\frac{\omega^2}{c^2}\right)\mathbf{E}_{\mathrm{sca}}^{(1)}(\mathbf{r},\omega) &= \frac{4\pi\mathrm{i}\omega}{c^2}\mathbf{J}^{(1)}(\mathbf{r},\omega)\\ \left(\{\nabla\times\nabla\times\} - \epsilon_2(\mathbf{r})\frac{\omega^2}{c^2}\right)\left\{\mathbf{E}_{\mathrm{sca}}^{(2)}(\mathbf{r},\omega) + \mathbf{E}_0^{(2)}(\mathbf{r},\omega)\right\} &= \frac{4\pi\mathrm{i}\omega}{c^2}\mathbf{J}^{(2)}(\mathbf{r},\omega)\end{aligned} \tag{S37}$$

The currents in right-hand-sides of Eq. (S37) are bound currents that are only nonzero where the first- and second-order susceptibilities of the $LiNbO_3$ sphere are nonzero, respectively, such that

$$\begin{aligned}\mathbf{J}^{(1)}(\mathbf{r},\omega) &= -\mathrm{i}\omega\chi_2^{(1)}(\mathbf{r})\mathbf{E}_0(\mathbf{r},\omega)\\ \mathbf{J}^{(2)}(\mathbf{r},\omega) &= -\mathrm{i}\omega\int_{-\infty}^{\infty}\left(\mathbf{E}_0(\mathbf{r},\omega') + \mathbf{E}_{\mathrm{sca}}^{(1)}(\mathbf{r},\omega')\right)\\ &\quad\cdot\boldsymbol{\chi}_2^{(2)}(\mathbf{r};\omega',\omega-\omega')\cdot\left(\mathbf{E}_0(\mathbf{r},\omega-\omega') + \mathbf{E}_{\mathrm{sca}}^{(1)}(\mathbf{r},\omega-\omega')\right)\,\frac{\mathrm{d}\omega'}{2\pi}\end{aligned} \tag{S38}$$

The former current can straightforwardly be seen to be large where $\omega = \pm\omega_0$ if the laser light is a monochromatic plane wave with an electric field

$$\mathbf{E}_0(\mathbf{r},\omega) = E_0\left[\pi\delta(\omega-\omega_0)\mathrm{e}^{\mathrm{i}\omega_0 z/c} + \pi\delta(\omega+\omega_0)\mathrm{e}^{-\mathrm{i}\omega_0 z/c}\right]\hat{\mathbf{x}} \tag{S39}$$

while for the same incoming field the leading factor of ω in the latter restricts the integral to be zero unless $\omega' = \pm\omega_0$ and $\omega = 2\omega'$. The currents drive the total fields through the Green's function solution to either wave equation,

$$\mathbf{E}^{(n)}_{\text{sca}}(\mathbf{r},\omega) + \mathbf{E}^{(n)}_0(\mathbf{r},\omega) = \frac{4\pi\mathrm{i}\omega}{c}\int \mathbf{G}_{\text{LNO}}(\mathbf{r},\mathbf{r}';\omega)\cdot\frac{\mathbf{J}^{(n)}(\mathbf{r}',\omega)}{c}\,\mathrm{d}^3\mathbf{r}' \tag{S40}$$

where the spherical dyadic Green's function $\mathbf{G}_{\text{LNO}}(\mathbf{r},\mathbf{r}';\omega)$ is given in the literature.[13] The Green's function, as we show below, is separable as $\mathbf{G}_{\text{LNO}}(\mathbf{r},\mathbf{r}';\omega) = \mathbf{G}_{\text{sca}}(\mathbf{r},\mathbf{r}';\omega)+\mathbf{G}_0(\mathbf{r},\mathbf{r};\omega)$ into a "scattering" part $\mathbf{G}_{\text{sca}}(\mathbf{r},\mathbf{r}';\omega)$ and a "vacuum-like" part $\mathbf{G}_0(\mathbf{r},\mathbf{r};\omega)$ such that

$$\begin{aligned}\mathbf{E}^{(n)}_{\text{sca}}(\mathbf{r},\omega) &= \frac{4\pi\mathrm{i}\omega}{c}\int \mathbf{G}_{\text{sca}}(\mathbf{r},\mathbf{r}';\omega)\cdot\frac{\mathbf{J}^{(n)}(\mathbf{r}',\omega)}{c}\,\mathrm{d}^3\mathbf{r}'\\ \mathbf{E}^{(n)}_0(\mathbf{r},\omega) &= \frac{4\pi\mathrm{i}\omega}{c}\int \mathbf{G}_0(\mathbf{r},\mathbf{r}';\omega)\cdot\frac{\mathbf{J}^{(n)}(\mathbf{r}',\omega)}{c}\,\mathrm{d}^3\mathbf{r}'\end{aligned} \tag{S41}$$

A similar solution can be found for the scattered electric field of the Au NPs. The incident field and second-order current $\mathbf{J}^{(2)}(\mathbf{r},\omega)$ can drive the response of each NP in the same manner that they drive the scattered fields of the $\mathrm{LiNbO_3}$ sphere, such that we can build a model that includes only these two sources and temporarily neglects interactions between the NPs and microsphere. Ignoring the effects of radiation, the scattered potential of the NP is

$$-\nabla\cdot\epsilon_1(\mathbf{r},\omega)\nabla\left\{\Phi_{\text{NP}}(\mathbf{r},\omega)+\Phi^{(2)}_0(\mathbf{r},\omega)\right\} = 4\pi\chi_1(\mathbf{r},\omega)\left[\nabla\cdot\nabla\Phi_0(\mathbf{r},\omega)+\frac{4\pi\mathrm{i}}{\omega}\nabla\cdot\mathbf{J}^{(2)}(\mathbf{r},\omega)\right] \tag{S42}$$

for $\chi_1(\mathbf{r},\omega) = \chi_1(\omega)\Theta(\mathbf{r}\in V_1)$ and $\mathbf{r}$ not on the boundary of the NP's volume V_1. Assuming the laser wavelength $\lambda_0 = 2\pi c/\omega_0$ is much longer than the extent of the NP, the incident potential can be written as $\Phi_0(\mathbf{r},\omega) \approx \mathbf{r}\cdot\lim_{k\to 0}\mathbf{E}_0(\mathbf{r},\omega) = xE_0\pi[\delta(\omega-\omega_0)+\delta(\omega+\omega_0)]$, where $k = \omega/c$. Similarly, $-\nabla\Phi^{(2)}_0(\mathbf{r},\omega) = \lim_{k\to 0}\mathbf{E}^{(2)}_0(\mathbf{r},\omega)$.

Assuming that the solutions to Eq. (S42) are near zero at $\pm\omega_0$, i.e. the NP does not respond strongly near the fundamental frequency of the laser, the term proportional to $\Phi_0(\mathbf{r},\omega)$ on the RHS can be dropped. Thus, with the dielectric function $\epsilon_1(\mathbf{r},\omega) = \epsilon_1(\omega)\Theta(r\le a_1)+1\Theta(r\ge a_1)$ defined such that the NP lies at the origin, one finds:

$$\Phi_{\text{NP}}(\mathrm{r},\omega)+\Phi^{(2)}_0(\mathbf{r},\omega) \approx \int G_{\text{NP}}(\mathbf{r},\mathbf{r}';\omega)\chi_1(\mathbf{r},\omega)\frac{4\pi\mathrm{i}}{\omega}\nabla\cdot\mathbf{J}^{(2)}(\mathbf{r},\omega)\,\mathrm{d}^3\mathbf{r}' \tag{S43}$$

wherein the (scalar) Green's function $G_{\text{NP}}(\mathbf{r},\mathbf{r}',\omega)$ is the standard solution to the Poisson equation in spherical coordinates.[3] Much like the dyadic Green's function for the $\mathrm{LiNbO_3}$ fields, $G_{\text{NP}}(\mathbf{r},\mathbf{r}',\omega) = G_{\text{sca}}(\mathbf{r},\mathbf{r}',\omega)+G_0(\mathbf{r},\mathbf{r}',\omega)$ is separable into a scattering part and a vacuum-like or free-space part. The former encodes the resonances of the NP, while the latter serves simply to satisfy the principle of superposition and is otherwise unimportant.

S.2.4.2 Construction of the Oscillator Model

The above definitions are clarified by the forms of both $\mathbf{G}_{\text{LNO}}$ and G_{NP}, which are cumbersome but manageable. The former, in the case where both the source charges and the

observer are outside the sphere's surface, is given by

$$
\begin{aligned}
G_{\mathrm{NP}}(\mathbf{r},\mathbf{r}';\omega)\Theta(r>a_1)\Theta(r'>a_1) &= \sum_{p=0}^{1}\sum_{\ell=1}^{\infty}\sum_{m=0}^{\ell}(2-\delta_{m0})\frac{(\ell-m)!}{(\ell+m)!}\frac{\ell[1-\epsilon_1(\omega)]}{\ell\epsilon_1(\omega)+(\ell+1)} \\
&\times \frac{a_1^{2\ell+1}}{r^{\ell+1}r'^{\ell+1}}P_{\ell m}(\cos\theta)P_{\ell m}(\cos\theta')S_p(m\phi)S_p(m\phi') + \frac{1}{|\mathbf{r}-\mathbf{r}'|}
\end{aligned}
\tag{S44}
$$

for a sphere centered at the origin. Each of the mode functions $(a_1^{\ell+1}/r^{\ell+1})P_{\ell m}(\cos\theta)S_p(m\phi)$ describes the spatial variation of the observables of an electric multipole mode with a characteristic response function[14]

$$
g_{p\ell m}(\omega) = \frac{\ell[1-\epsilon_1(\omega)]}{\ell\epsilon_1(\omega)+(\ell+1)} \tag{S45}
$$

that describes its oscillations in time. Here, ℓ and m give the order and degree of each mode's corresponding spherical harmonic and p the reflection symmetry of each mode across the x-axis.

The Green's function of the $LiNbO_3$ sphere can be similarly expanded using a set of so-called quasinormal geometric resonances of both magnetic and electric multipole symmetry[2] with response functions that depend on the same spherical harmonic symmetry parameters p, ℓ, m. For an observer inside the sphere, this Green's function is:

$$
\begin{aligned}
\mathbf{G}_{\mathrm{LNO}}(\mathbf{r},\mathbf{r}';\omega)\Theta(r<a_2) &= \frac{\mathrm{i}\omega}{4\pi c}\sum_{p\ell m}\left[C^{>}_{p\ell m}(\omega)\boldsymbol{\mathcal{M}}_{p\ell m}(\mathbf{r},\sqrt{\epsilon_2}k)\mathbf{M}_{p\ell m}(\mathbf{r}',k)\right. \\
&\left.+D^{>}_{p\ell m}(\omega)\boldsymbol{\mathcal{N}}_{p\ell m}(\mathbf{r},\sqrt{\epsilon_2}k)\mathbf{N}_{p\ell m}(\mathbf{r}',k)\right]\Theta(r<a_2)\Theta(r'>a_2) \\
&+\sqrt{\epsilon_2}\frac{\mathrm{i}\omega}{4\pi c}\sum_{p\ell m}\left[C^{<}_{p\ell m}(\omega)\boldsymbol{\mathcal{M}}_{p\ell m}(\mathbf{r},\sqrt{\epsilon_2}k)\boldsymbol{\mathcal{M}}_{p\ell m}(\mathbf{r}',\sqrt{\epsilon_2}k)\right. \\
&\left.+D^{<}_{p\ell m}(\omega)\boldsymbol{\mathcal{N}}_{p\ell m}(\mathbf{r},\sqrt{\epsilon_2}k)\boldsymbol{\mathcal{N}}_{p\ell m}(\mathbf{r}',\sqrt{\epsilon_2}k)\right]\Theta(r<a_2)\Theta(r'<a_2) \\
&+\mathbf{G}_0(\mathbf{r},\mathbf{r}';\sqrt{\epsilon_2}\omega)\Theta(r<a_2)\Theta(r'<a_2)
\end{aligned}
\tag{S46}
$$

where again the sphere is centered at the origin. The mode functions $\boldsymbol{\mathcal{M}}_{p\ell m}$ and $\boldsymbol{\mathcal{N}}_{p\ell m}$ are identical to the regularized vector spherical harmonics given in (S16) but with the spherical Hankel functions replaced with spherical Bessel functions, $h_\ell^{(1)}(x) \to j_\ell(x)$. The Green's function for an observer outside the sphere is, similarly:

$$
\begin{aligned}
\mathbf{G}_{\mathrm{LNO}}(\mathbf{r},\mathbf{r}';\omega)\Theta(r>a_2) &= \frac{\mathrm{i}\omega}{4\pi c}\sum_{p\ell m}\left[A^{>}_{p\ell m}(\omega)\mathbf{M}_{p\ell m}(\mathbf{r},k)\mathbf{M}_{p\ell m}(\mathbf{r}',k)\right. \\
&\left.+B^{>}_{p\ell m}(\omega)\mathbf{N}_{p\ell m}(\mathbf{r},k)\mathbf{N}_{p\ell m}(\mathbf{r}',k)\right]\Theta(r>a_2)\Theta(r'>a_2) \\
&+\sqrt{\epsilon_2}\frac{\mathrm{i}\omega}{4\pi c}\sum_{p\ell m}\left[A^{<}_{p\ell m}(\omega)\mathbf{M}_{p\ell m}(\mathbf{r},k)\boldsymbol{\mathcal{M}}_{p\ell m}(\mathbf{r}',\sqrt{\epsilon_2}k)\right. \\
&\left.+B^{<}_{p\ell m}(\omega)\mathbf{N}_{p\ell m}(\mathbf{r},k)\boldsymbol{\mathcal{N}}_{p\ell m}(\mathbf{r}',\sqrt{\epsilon_2}k)\right]\Theta(r>a_2)\Theta(r'<a_2) \\
&+\mathbf{G}_0(\mathbf{r},\mathbf{r}';\omega)\Theta(r>a_2)\Theta(r'>a_2)
\end{aligned}
\tag{S47}
$$

with the forms of the unwieldy response functions $A_{p\ell m}(\omega)$, $B_{p\ell m}(\omega)$, $C_{p\ell m}(\omega)$, and $D_{p\ell m}(\omega)$ described in Ref. 13. Further, $\mathbf{G}_0(\mathbf{r}, \mathbf{r}'; \omega)$ is the Green's function of free space.

To compare the responses of the NP and microsphere, we can see that $B^<_{p\ell m}(\omega)$ obeys the simple relation $\lim_{k\to 0}(\sqrt{\epsilon_2})^\ell B_{p\ell m}(\omega) - 1 = \ell(1-\epsilon_2)/(\ell\epsilon_2 + \ell + 1)$. This gives us a foundation from which to make quantitative comparisons between the oscillator parameters assigned to each response in either particle. In detail, using the Drude-Lorentz dielectric model of Au from Section S.1.2.2, we can see that the dipole response function $g_1(\omega)$ describes the oscillations of three coupled modes, one which has primarily free-electron character (the dipole plasmon) two others which are mostly comprised of the material's interband resonances. Explicitly,

$$g_{p1m}(\omega) = -\frac{e^2}{a_1^3}\left[\frac{1}{2\omega_1\mu_1}\left(\frac{\mathrm{e}^{\mathrm{i}\psi_1}}{\Omega_1 - \omega} + \frac{\mathrm{e}^{-\mathrm{i}\psi_1}}{\Omega_1^* + \omega}\right) + \sum_{i=1}^{2}\frac{1}{2\omega_{\mathrm{L}_i}\mu_{\mathrm{L}_i}}\left(\frac{\mathrm{e}^{\mathrm{i}\psi_{\mathrm{L}_i}}}{\Omega_{\mathrm{L}_i} - \omega} + \frac{\mathrm{e}^{-\mathrm{i}\psi_{\mathrm{L}_i}}}{\Omega_{\mathrm{L}_i}^* + \omega}\right)\right] \tag{S48}$$

wherein the mass μ, resonance frequency ω, complex eigenvalue Ω, and phase offset ψ of the plasmon are labeled with subscript 1, and the oscillator parameters of the fictitious oscillator are labeled with subscripts L_i. Explicit values of these parameters are given in Section S.2.2.

As is also shown in Section S.2.2, there are many modes of the $LiNbO_3$ sphere that have significant response magnitudes in the range of laser energies in which SHG is observed (~2.3–2.4 eV, see Figure 4c). To simplify their description, we use an expansion of the $LiNbO_3$ mode response functions:

$$\begin{aligned}
(\sqrt{\epsilon_2})^\ell A^<_{p\ell m}(\omega) - 1 &\approx -\frac{e^2}{a_2^3}\sum_j \frac{1}{2\omega_{M\ell j}\mu_{M\ell j}}\left(\frac{\mathrm{e}^{\mathrm{i}\psi_{M\ell j}}}{\Omega_{M\ell j} - \omega} + \frac{\mathrm{e}^{-\mathrm{i}\psi_{M\ell j}}}{\Omega_{M\ell j}^* + \omega}\right)\\
(\sqrt{\epsilon_2})^\ell B^<_{p\ell m}(\omega) - 1 &\approx -\frac{e^2}{a_2^3}\sum_j \frac{1}{2\omega_{E\ell j}\mu_{E\ell j}}\left(\frac{\mathrm{e}^{\mathrm{i}\psi_{E\ell j}}}{\Omega_{E\ell j} - \omega} + \frac{\mathrm{e}^{-\mathrm{i}\psi_{E\ell j}}}{\Omega_{E\ell j}^* + \omega}\right)
\end{aligned} \tag{S49}$$

where the labels M, E denote magnetic- and electric-type oscillator parameters and the indices j label the different resonances of common angular symmetry p, ℓ, m but different node structure along the radial coordinate. Because only a single mode of the set of modes j lies in the energetic range of interest, we will drop the sum over j and the corresponding labels in the following discussion.

With explicit value of the relevant $LiNbO_3$ oscillator parameters given in Section S.2.2, the formal definitions of the oscillator moments $d_\nu(\omega)$ of the dipole plasmons oriented along the three cardinal axes $\nu = r, \theta, \phi$ as well the $LiNbO_3$ Mie multipoles $\boldsymbol{\beta}$ can be defined analytically. In particular, with a vector spherical harmonic expansion of the laser field[15]

$$\mathbf{E}_0(\mathbf{r}, \omega) = E_0\left[\pi\delta(\omega - \omega_0) + \pi\delta(\omega + \omega_0)\right]\sum_\ell \mathrm{i}^\ell \frac{2\ell + 1}{\ell(\ell + 1)}\left[\boldsymbol{\mathcal{M}}_{1\ell 1}(\mathbf{r}, k) - \mathrm{i}\boldsymbol{\mathcal{N}}_{0\ell 1}(\mathbf{r}, k)\right] \tag{S50}$$

the second-order fields of a *bare* $LiNbO_3$ microsphere centered at the origin can be written

as

$$
\begin{aligned}
\mathbf{E}^{(2)}_{\mathrm{sca}}(\mathbf{r},\omega) &= \sum_{\boldsymbol{\beta}} \frac{1}{a_2^{\ell+2}} \left[\rho_{\boldsymbol{\beta}}(\omega)\mathbf{X}_{\boldsymbol{\beta}}(\mathbf{r},k_{\boldsymbol{\beta}}) + \rho^*_{\boldsymbol{\beta}}(-\omega)\mathbf{X}^*_{\boldsymbol{\beta}}(\mathbf{r},k_{\boldsymbol{\beta}})\right] \\
\mathbf{E}^{(2)}_0(\mathbf{r},\omega) &= -\mathrm{i}\frac{\omega_0^9}{4c^9}\pi\delta(\omega-2\omega_0)\epsilon_2(\epsilon_2-1)^2E_0^2\chi_2^{(2)}(\omega_0,\omega_0) \\
&\quad \times \sum_{\boldsymbol{\gamma}\boldsymbol{\alpha}\boldsymbol{\alpha}'} w_{\boldsymbol{\alpha}}(\omega_0)w_{\boldsymbol{\alpha}'}(\omega_0)I_{\boldsymbol{\gamma}\boldsymbol{\alpha}\boldsymbol{\alpha}'}(k_{\boldsymbol{\gamma}},\sqrt{\epsilon_2}k_0,\sqrt{\epsilon_2}k_0;0,a_2)\boldsymbol{\mathcal{X}}_{\boldsymbol{\gamma}}(\mathbf{r},\omega) + \text{c.c.r.}
\end{aligned}
\tag{S51}
$$

Here, as defined in the main text, $\rho_{\boldsymbol{\beta}}(\omega)$ are the multipole moment magnitudes of the Mie resonances of the sphere. Their explicit form is complicated and is detailed below. The index $\boldsymbol{\gamma} = \{T''', p''', \ell''', m'''\}$ is another collective Mie index like $\boldsymbol{\alpha}$ and $\boldsymbol{\beta}$. It labels modes that contribute to the field near $2\omega_0$ but, unlike $\boldsymbol{\beta}$, is not restricted to counting only modes with strongly resonant behavior. In the calculations of this work, it is taken to label all Mie modes with $\ell > 1$ up to a cutoff $\ell = 12$ after which the contribution of successive terms in the sum is negligible. Further, the term c.c.r. is simply the frequency reversed ($\omega \to -\omega$) complex conjugate of the first term of the second equality above such that $\mathbf{E}^{(2)}_0(\mathbf{r},\omega)$ satisfies the Fourier reality condition $\mathbf{E}^{(2)*}_0(\mathbf{r},\omega) = \mathbf{E}^{(2)}_0(\mathbf{r},-\omega)$.

The functions $w_{\boldsymbol{\alpha}}(\omega)$ inside the definition of the second-order vacuum-like field are the weights

$$
w_{\boldsymbol{\alpha}}(\omega_0) = \begin{cases}
\mathrm{i}^{\ell}\sqrt{\dfrac{2\ell+1}{2\ell(\ell+1)}\dfrac{(\ell+1)!}{(\ell-1)!}}C^{<}_{p\ell m}(\omega)R_\ell(k_0,\sqrt{\epsilon_2}k_0;0,a_2), & T = M \\[2ex]
\begin{aligned}-\mathrm{i}^{\ell+1}\sqrt{\dfrac{2\ell+1}{2\ell(\ell+1)}\dfrac{(\ell+1)!}{(\ell-1)!}}D^{<}_{p\ell m}(\omega)\bigg[&\dfrac{\ell+1}{2\ell+1}R_{\ell-1}(k_0,\sqrt{\epsilon_2}k_0;0,a_2) \\ &+ \dfrac{\ell}{2\ell+1}R_{\ell+1}(k_0,\sqrt{\epsilon_2}k_0;0,a_2)\bigg]\end{aligned}, & T = E
\end{cases}
\tag{S52}
$$

wherein

$$
\begin{aligned}
R_\ell(k,k';a,b) &= \int_a^b r^2 j_\ell(kr)j_\ell(k'r)\,\mathrm{d}r \\
&= \frac{r^2}{k^2-k'^2}\left[k'^2 j_\ell(kr)j_{\ell-1}(k'r) - k^2 j_{\ell-1}(kr)j_\ell(k'r)\right]_a^b
\end{aligned}
\tag{S53}
$$

is an overlap integral over the radial components $j_\ell(kr)$ of the fundamental mode functions. Further, the functions $I_{\boldsymbol{\alpha}_1\boldsymbol{\alpha}_2\boldsymbol{\alpha}_3}$ are overlap triple integrals

$$
\begin{aligned}
I_{\boldsymbol{\alpha}_1\boldsymbol{\alpha}_2\boldsymbol{\alpha}_3}(k_1,k_2,k_3;a,b) = &\int_0^{2\pi}\int_0^{\pi}\int_a^b \boldsymbol{\mathcal{X}}_{\boldsymbol{\alpha}_1}(\mathbf{r}',k_1)\cdot\left[\boldsymbol{\mathcal{X}}_{\boldsymbol{\alpha}_2}(\mathbf{r}',k_2)\cdot\mathbf{1}_3\cdot\boldsymbol{\mathcal{X}}_{\boldsymbol{\alpha}_3}(\mathbf{r}',k_3)\right] \\
&\times r'^2\sin\theta'\,\mathrm{d}r'\,\mathrm{d}\theta'\,\mathrm{d}\phi'
\end{aligned}
\tag{S54}
$$

wherein $\boldsymbol{\mathcal{X}}_{Tp\ell m}(\mathbf{r},k) = \boldsymbol{\mathcal{M}}_{p\ell m}(\mathbf{r},k)\delta_{T,M} + \boldsymbol{\mathcal{N}}_{p\ell m}(\mathbf{r},k)\delta_{T,E}$.

Using the definition of the dipole polarizabilities $\alpha_\nu(\omega)$ given in Section S.2.2.2, we can define the motion of each component of a single Au dipole and each Mie resonance in the absence of their mutual coupling as

$$\begin{aligned} d_\nu(\omega) &= \frac{e^2}{2\omega_1\mu_\nu}\left(\frac{\mathrm{e}^{\mathrm{i}\psi_1}}{\Omega_1-\omega} + \frac{\mathrm{e}^{-\mathrm{i}\psi_1}}{\Omega_1^*+\omega}\right)\hat{\mathbf{e}}_\nu\cdot\mathbf{E}_0^{(2)}(\mathbf{r}_0,\omega) \\ \rho_{\boldsymbol{\beta}}(\omega) &= \frac{e^2 a_2^{\ell-2}}{2\omega_{\boldsymbol{\beta}}\mu_{\boldsymbol{\beta}}}\frac{\mathrm{e}^{\mathrm{i}\psi_{\boldsymbol{\beta}}}}{\Omega_{\boldsymbol{\beta}}-\omega}E_0\pi\delta(\omega-2\omega_0)C_{\boldsymbol{\beta}}(\omega_0) \end{aligned} \tag{S55}$$

In accordance with the main text, the dipole is assumed to exist at $\mathbf{r}_0 = (a_1+a_2)\hat{\mathbf{r}}(\theta_0,\phi_0)$ and to be driven by the second-order vacuum-like field. We ignore any modifications to $\mathbf{E}_0^{(2)}(\mathbf{r},\omega)$ that are produced by $d_\nu(\omega)$. The Mie resonances of the $LiNbO_3$ are driven through the upconversion process, as detailed by the constants

$$\begin{aligned} C_{\boldsymbol{\beta}}(\omega_0) = {}& \mathrm{i}\frac{\omega_0^9}{4c^9}\frac{\epsilon_2^{\frac{3}{2}}(\epsilon_2-1)^2}{(\sqrt{\epsilon_2})^\ell}E_0\chi_2^{(2)}(\omega_0,\omega_0) \\ & \times\sum_{\boldsymbol{\alpha}\boldsymbol{\alpha}'} w_{\boldsymbol{\alpha}}(\omega_0)w_{\boldsymbol{\alpha}'}(\omega_0)I_{\boldsymbol{\beta}\boldsymbol{\alpha}\boldsymbol{\alpha}'}(\sqrt{\epsilon_2}k_{\boldsymbol{\beta}},\sqrt{\epsilon_2}k_0,\sqrt{\epsilon_2}k_0;0,a_2) \end{aligned} \tag{S56}$$

Finally, to reproduce the equations of motion of Eq. (1), we must define the external forces acting on the particle moments and introduce the coupling forces to Eq. (S55). The former can be quickly written as $F_{1\nu}(\omega) = e\hat{\mathbf{e}}_\nu\cdot\mathbf{E}_0^{(2)}(\mathbf{r}_0,\omega)$ and $F_{2\boldsymbol{\beta}}(\omega) = eE_0\pi\delta(\omega - 2\omega_0)C_{\boldsymbol{\beta}}(\omega)$. The latter arise from the interaction energy $U(t) = -\mathbf{d}(t)\cdot\mathbf{E}_{\mathrm{sca}}^{(2)}(\mathbf{r}_0,t)$, where $\mathbf{d}(t) = \sum_\nu d_\nu(t)\hat{\mathbf{e}}_\nu$. With the identities

$$\begin{aligned} -a_2^{\ell-1}\frac{\partial U(t)}{\partial\rho_{\boldsymbol{\beta}}(t)} &= \sigma_{\boldsymbol{\beta}\nu}d_\nu(t) \\ -a_2^{\ell-1}\frac{\partial U(t)}{\partial d_\nu(t)} &= \sigma_{\boldsymbol{\beta}\nu}\rho_{\boldsymbol{\beta}}(t) \end{aligned} \tag{S57}$$

one is immediately delivered the equations of motion in the main text with $\sigma_{\boldsymbol{\beta}\nu} = (e^2/a_2^3)\hat{\mathbf{e}}_\nu\cdot\mathbf{X}_{\boldsymbol{\beta}}(\mathbf{r}_0,k_{\boldsymbol{\beta}})$. A detailed description of the solutions to the equations of motion are given in Section S.1, and an analysis of the contribution of the various modes of the $LiNbO_3$ sphere to enhancement signal is shown in Figure S14.

In closing, we note that, as the solutions to the equations of motion discussed in the main text and highlighted in Section S.1 involve a second perturbation expansion of the equations of motion of each Mie resonance, two sets of superscripts arise that correspond to two separate expansions. In order to clarify the notation, we can see that no terms in the solution to the wave equation are kept beyond second order, such that we can replace superscripted variable names with script names. Further, we can drop the superscripts of the first-order terms altogether, such that, elsewhere in the SI and main text, we let $\mathbf{E}_{\mathrm{sca}}^{(2)}\to\boldsymbol{\mathcal{E}}_{\mathrm{sca}}$, $\mathbf{E}_0^{(2)}\to\boldsymbol{\mathcal{E}}_0$, and $\mathbf{E}_{\mathrm{sca}}^{(1)}\to\mathbf{E}_{\mathrm{sca}}$.

S.3 Additional Figures and Tables

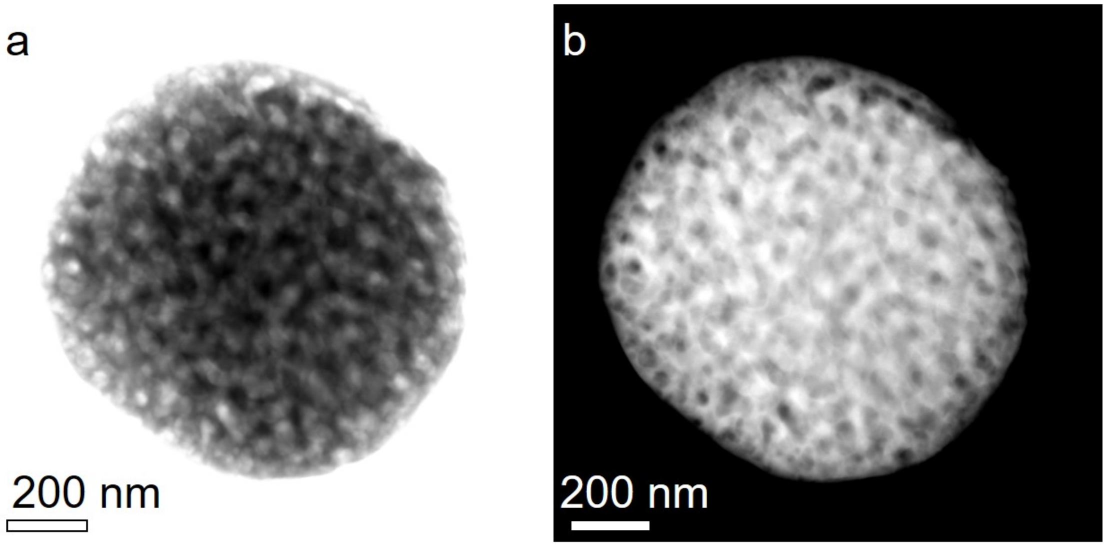

Figure S1: **Scanning transmission electron microscopy (STEM) images.** $LiNbO_3$ particle images in (a) bright-field and (b) dark-field modes.

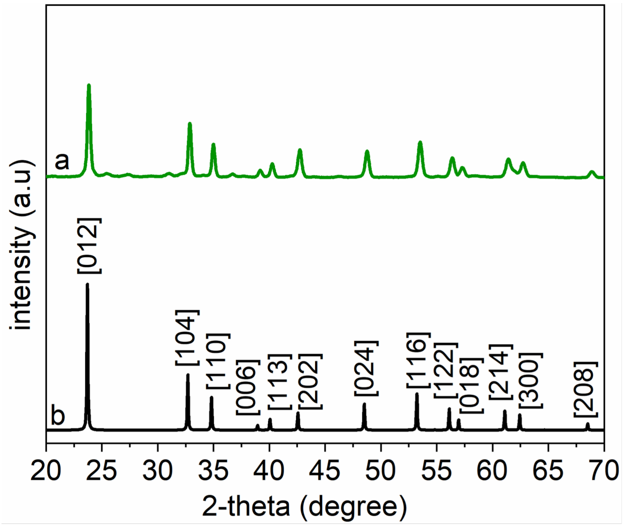

Figure S2: **Powder X-ray diffraction patterns.** (a) $LiNbO_3$ particles; and (b) a reported $LiNbO_3$ reference (JCPDS No. 020-0631).

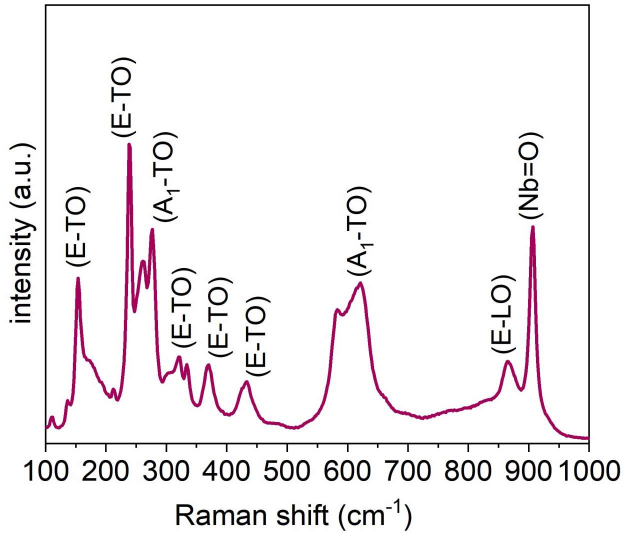

Figure S3: **Typical Raman spectrum for $LiNbO_3$ particles.** The spectra indicated the formation of a pure rhombohedral phase in the products.

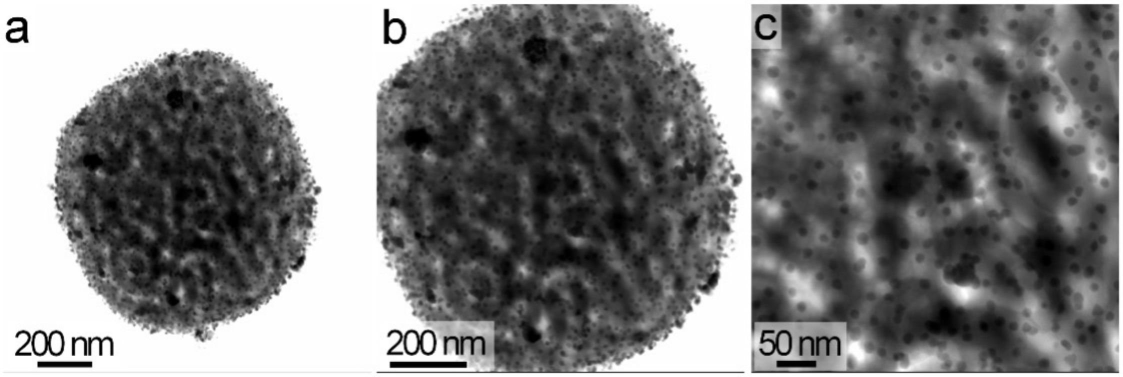

Figure S4: **STEM images of hybrid particles.** Assemblies of Au-$LiNbO_3$ hybrid particles as characterized by STEM operating in a bright-field mode.

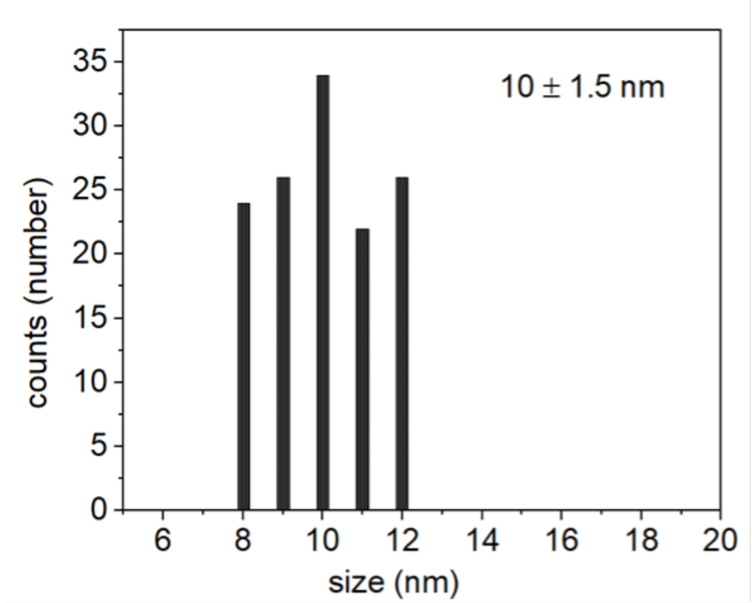

Figure S5: **Analysis of Au NP sizes.** Histogram depicting the size distribution of the diameters of the gold nanoparticles (NPs) present on the surfaces of the hybrid Au-$LiNbO_3$ nanostructures. This analysis included measurements obtained from 125 independent Au NPs. The variance of 1.5 nm is reported as one standard deviation from the calculated mean of 10 nm.

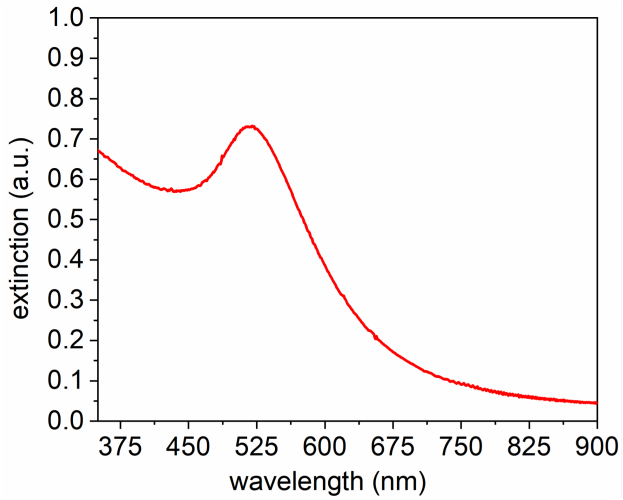

Figure S6: **Au NP absorbance.** Ultraviolet (UV)-visible absorbance spectrum of ∼10-nm diameter Au NPs suspended in an aqueous solution. This spectrum indicates the plasmonic band for the nanoparticles was centered at ∼520 nm, which is close to the 530 nm plasmonic band for hybrid Au-$LiNbO_3$ hybrid particles.

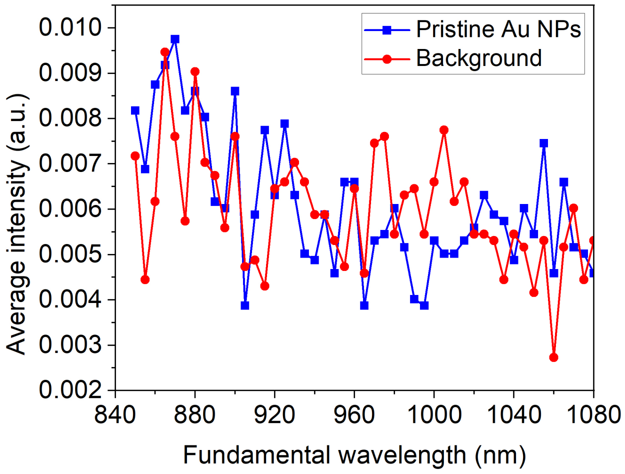

Figure S7: **Minimal SHG from Au NPs.** The SHG analyses for pristine ∼10-nm diameter gold nanoparticles recorded by sweeping the excitation laser wavelength from 850 to 1,080 nm with a step size of 5 nm. These results indicate a lack of SHG response in the centrosymmetric Au NPs.

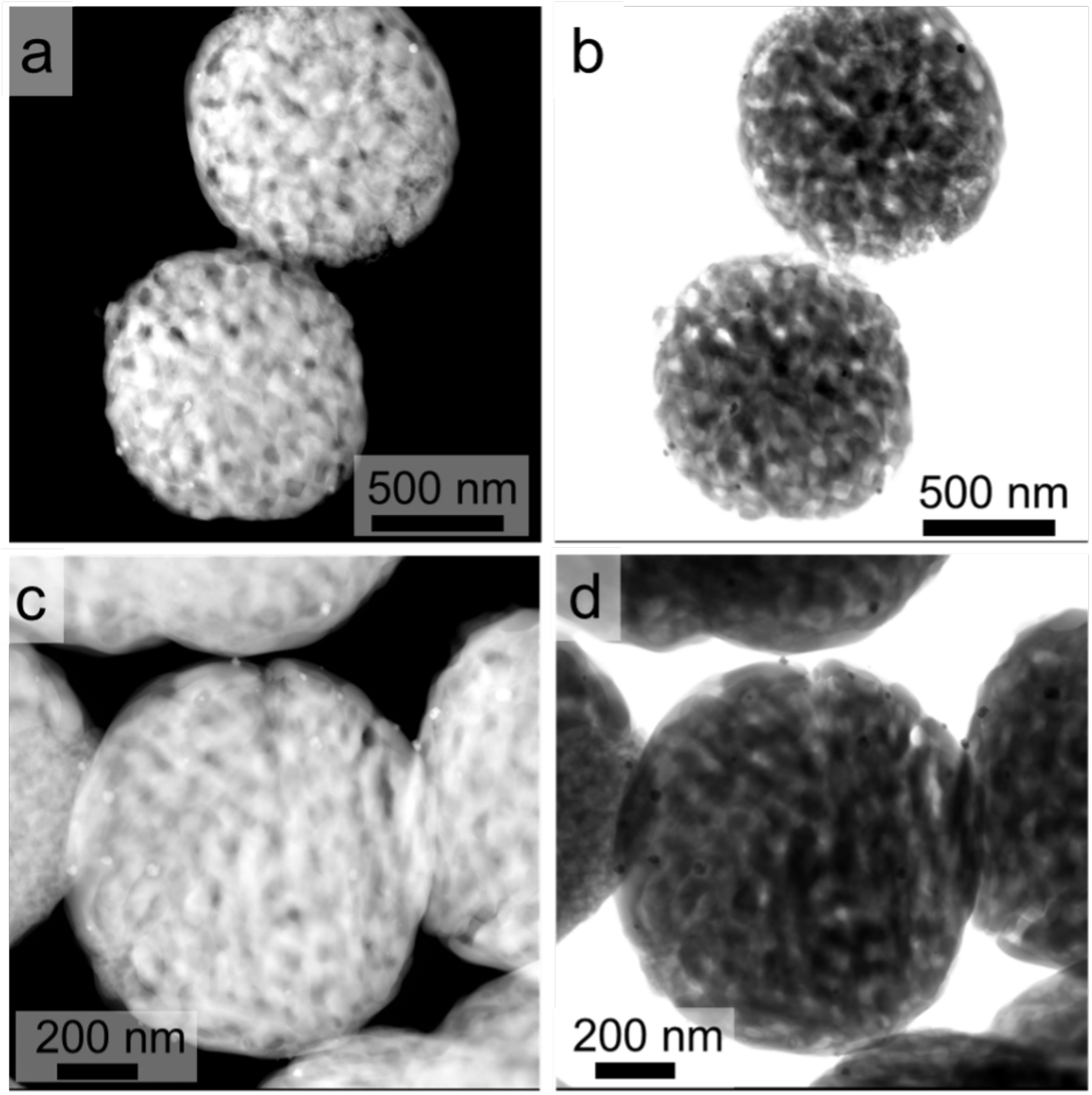

Figure S8: **Images of low NP-loading hybrid nanostructures.** Hybrid Au-$LiNbO_3$ particles prepared using 0.1 mL of a 5 mM $HAuCl_4$ solution to perform the *in situ* synthesis of the Au NPs. The resulting products were characterized by scanning transmission electron microscopy (STEM) operating in: (a), (c) a high-angle annular dark-field (HAADF) mode; and (b), (d) a bright-field mode.

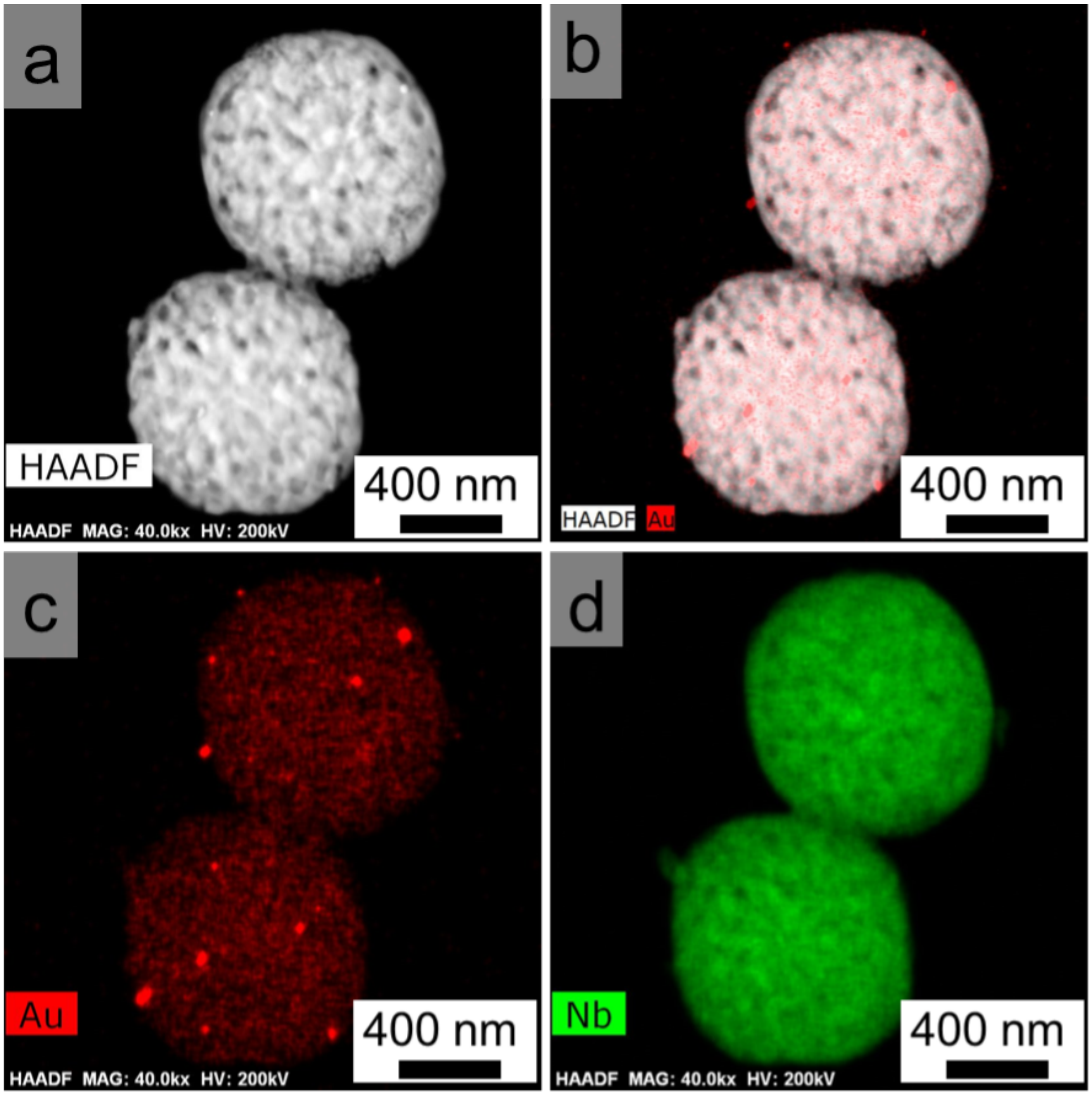

Figure S9: **Elemental analysis of low NP-loading hybrid nanostructures.** Energy dispersive X-ray spectroscopy (EDS) analysis of the hybrid Au-$LiNbO_3$ particles prepared using 0.1 mL of 5 mM $HAuCl_4$. These images show (a) a HAADF image obtained by STEM techniques, (b) an overlay of the Au NPs (as detected by EDS) on the HAADF image of the assemblies, (c) an EDS map of the Au NPs, and (d) an EDS map of Nb within these assemblies.

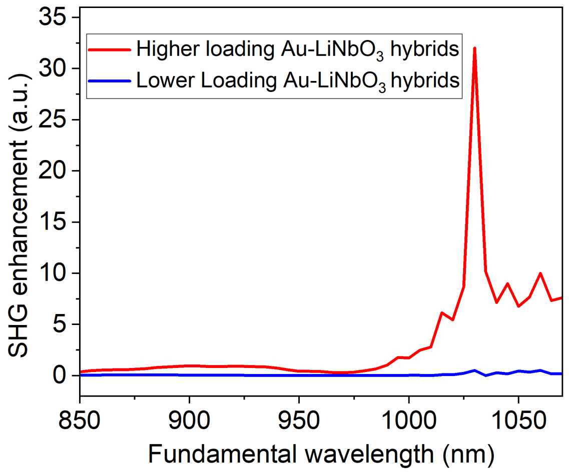

Figure S10: **SHG ehancement spectra comparison.** Evaluation of the enhancement in the SHG signal as a function of the fundamental wavelength (i.e., ranging from 850 to 1,070 nm) of the incident laser for the individual hybrid Au-$LiNbO_3$ particles prepared with a lower loading of Au NPs. After normalization of the SHG output of the individual Au-$LiNbO_3$ hybrids to the SHG output of the bare $LiNbO_3$, enhancement factors were plotted against the FWs of the measurement.

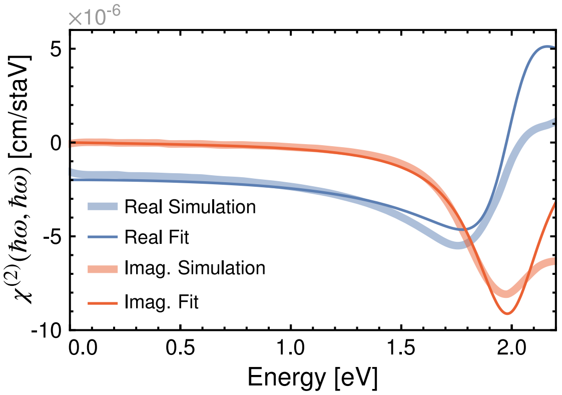

Figure S11: **$LiNbO_3$ second-order susceptibility fit.** Comparison of the real and imaginary parts of the magnitude $\chi^{(2)}(\omega,\omega) \equiv \hat{\mathbf{n}} \cdot \boldsymbol{\chi}_2^{(2)}(\omega,\omega) \cdot \hat{\mathbf{n}}$ of the second-order susceptibility in the limit where the output frequency is twice the input frequency ($\hat{\mathbf{n}}$ is any real unit vector).

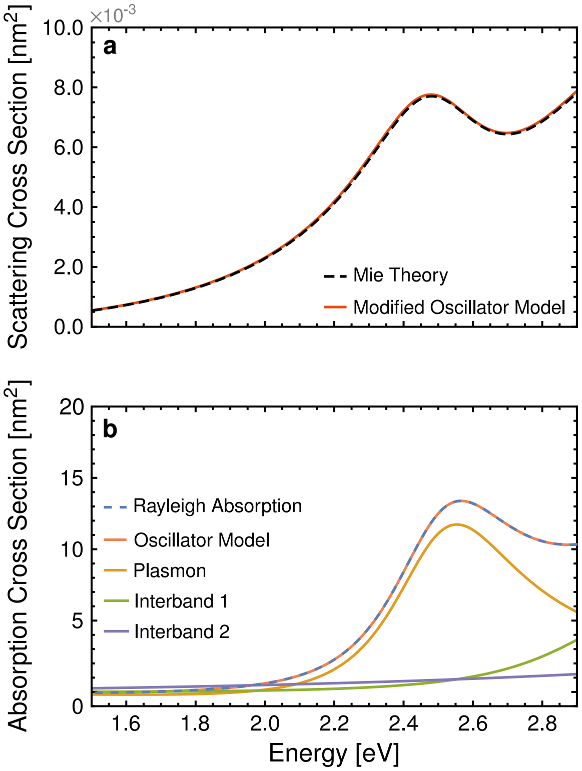

Figure S12: **Model Au NP plasmon spectra.** Comparison of the plasmon oscillator model with the (a) scattering and (b) absorption cross sections of the dipole modes of a 5-nm-radius Au sphere, respectively. The corresponding Mie and Rayleigh observables are calculated directly from the Au dielectric function as shown in Ref. 15. Panel (a) highlights the more dominant role played by high-energy resonances in Au on the blue side of the plasmon spectrum, while panel (b) shows the separate and important contributions to the total absorption cross section from the plasmon and interband oscillators in the energy range of the observed SHG.

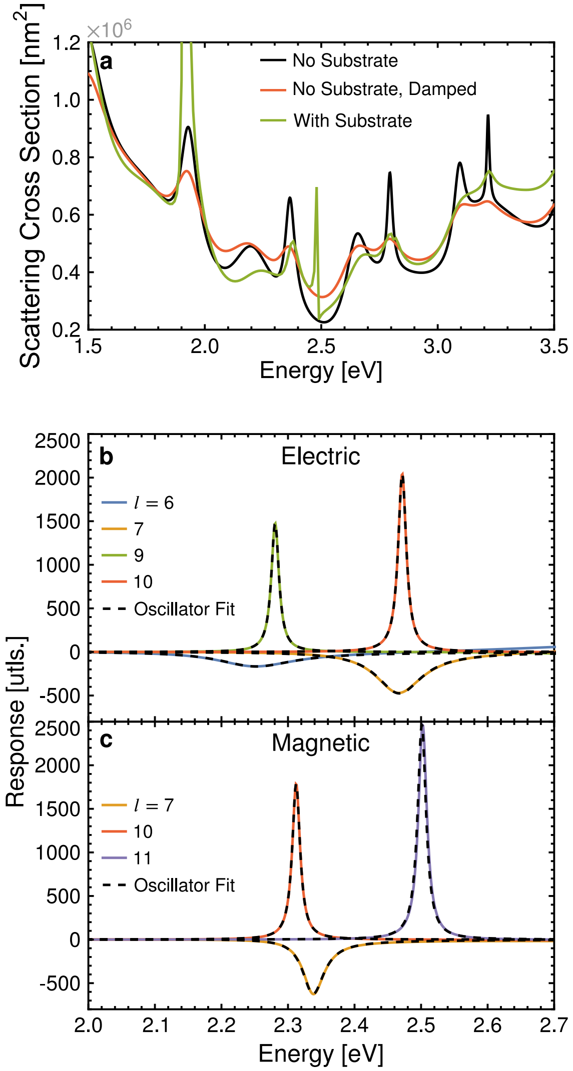

Figure S13: **Analysis of $LiNbO_3$ mode structure.** (a) Comparison of the scattering cross section of a 250 nm radius sphere with dielectric function $\epsilon = 5.0$ with spectra generated by two strategies for accounting for substrate effects. In red, the modification of Mie theory with a complex dielectric function $\epsilon = 5.0 + 0.2i$, and in green, the simulated spectrum of the unmodified sphere on a semi-infinite substrate of dielectric 2.25.[16] (b), (c) Numerical fit (dashed lines) of the imaginary parts of the oscillator model response functions of the (b) electric- and (c) magnetic-type $LiNbO_3$ Mie modes to the exact functions (solid lines) $(\sqrt{\epsilon_2})^\ell A^<_{p\ell m}(\omega) - 1$ and $(\sqrt{\epsilon_2})^\ell B^<_{p\ell m}(\omega) - 1$, respectively (see Eq. [S49]).

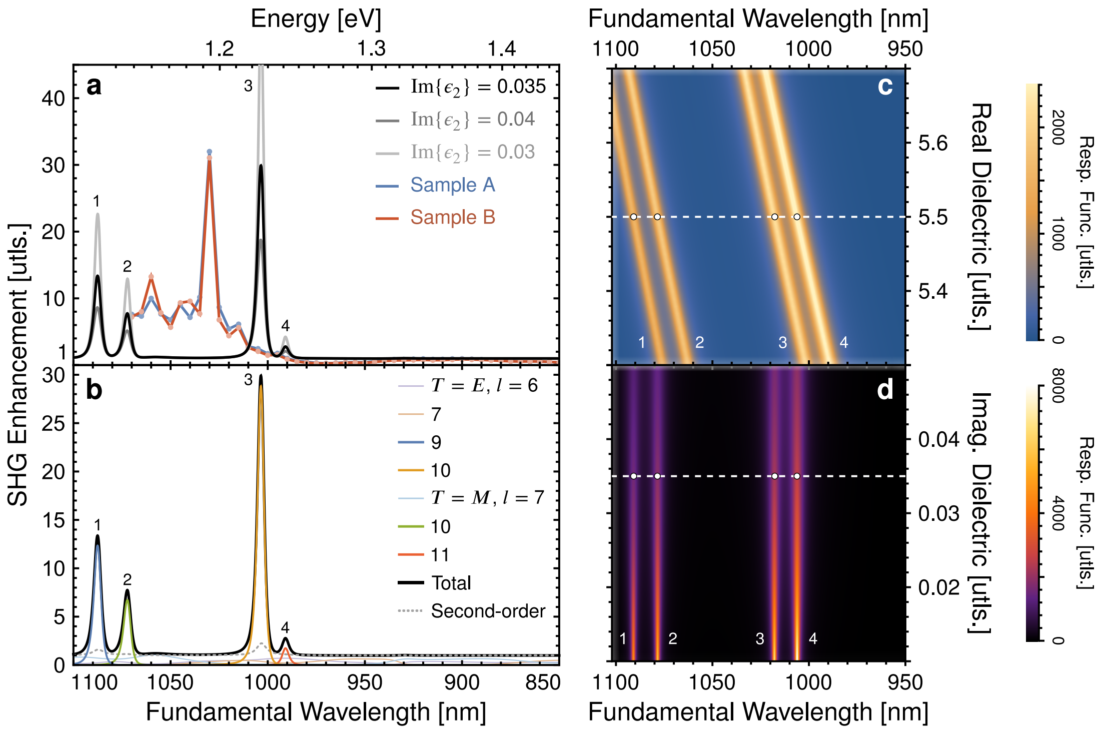

Figure S14: **Additional details of theoretical SHG enhancement spectra.** (a) Reproduction of the theoretical and experimental SHG enhancement spectra from Figure 4c. The theory (black)is shown without phenomenological shifts, along with a demonstration of the strong changes in the enhancement magnitude with small increases (dark gray) or decreases (light gray) to the imaginary part of ϵ_2. The four theoretical peaks are numbered for convenience and are plotted along with experimental SHG data (Sample A [blue] and Sample B [red]) for comparison. (b) Reconstruction of the theoretical SHG enhancement spectrum (black) from its constituent parts, highlighting the contributions of the modes of varying values of T and ℓ (colors). For simplicity, the contributions of modes of common T and ℓ are added together. The total SHG signal calculated to second order (gray, dashed) is shown as well, highlighting the importance of the nanoscopic energy-transfer described by the third-order contributions. (c) Demonstration of the strong dependence of the resonance positions of four main peaks of the theoretical spectrum on the real part of ϵ_2 with $\mathrm{Im}\{\epsilon_2\} = 0.035$. The plotted curve (shown in color) at each value of $\mathrm{Re}\{\epsilon_2\}$ is the imaginary part of the sum of the mode response functions (see Eq. [S49]) for the four peaks. The white dashed line indicates the value $\mathrm{Re}\{\epsilon_2\} = 5.5$ used in the main text, while the white dots lite at the locations of the numbered peaks in (a) and (b). (d) Showcase of the rapid decrease of γ_β with $\mathrm{Im}\{\epsilon_2\}$. The color plot is a collection of curves as in (c) but with the imaginary part of the $LiNbO_3$ dielectric allowed to vary and $\mathrm{Re}\{\epsilon_2\} = 5.5$. The dashed white line indicates the value $\mathrm{Im}\{\epsilon_2\} = 0.035$ used in the main text.

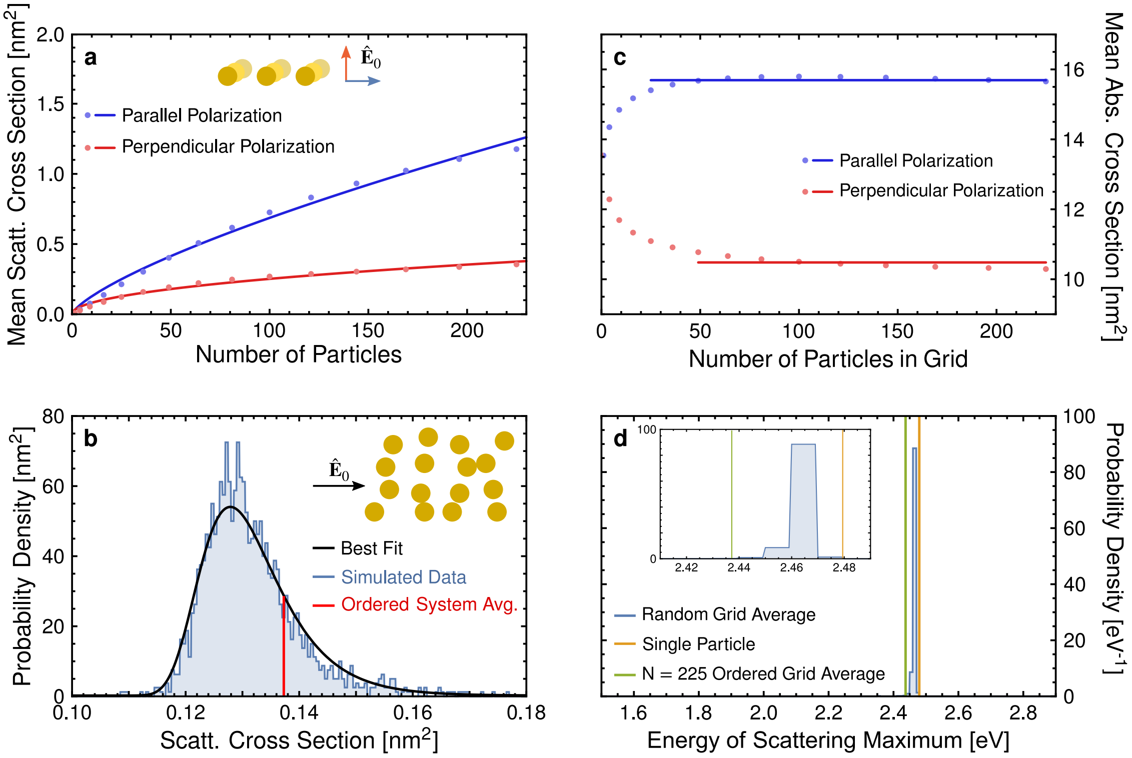

Figure S15: **Model Au NP ensemble behaviors.** (a) Simulated average scattering cross sections of Au nanospheres organized in square grids of $N \leq 225$ particles with light polarized parallel to the grid plane (blue points) and perpendicular (red points). The spheres have radii of $a_1 = 5$ nm and a minimum interparticle spacing of 10 nm. Fits to the data (lines) using a simple exponential function are used to extrapolate approximate upper bounds for the values of these cross sections when $N = 1{,}000$. (b) Distribution of scattering cross sections from 100 simulations of disordered grids of $N = 16$ Au NPs. The grids were arranged as in (a) but with each NP randomly shifted between -a_1 and a_1 in the in-plane directions and up to $\pm 2a_1$ along the out-of-plane axis such that no two particles overlap. The observed probability density (blue) is fit to an extreme value distribution (black) to estimate the proportion of the particles with cross sections lower and higher than the average from the ordered grid of 16 particles (red). (c) Demonstration that the absorption cross sections of the Au NPs approach stable values as the number of particles in the ensemble increases, both for parallel (blue) and perpendicular (red) incident light. Points show ensemble averages of the per-particle cross section maxima for ensembles of size $N = 1$ to 225, while the solid lines show the extracted averages of 15.7 nm^2 for parallel light and 10.5 nm^2 for perpendicular. The averages are drawn over the range of data points from which they are calculated. (d) Probability density of the spectral position of the scattering maxima of the NPs from 100 simulations of $N = 16$ NP ensembles. The energy of the scattering maximum of a single particle (see Figure S12a) and the average of the energies of the scattering maxima from a simulation of an $N = 225$ NP ensemble are shown for reference. Inset: A zoomed-in picture of the probability density, with identical quantities and units plotted on the axes as the main figure.

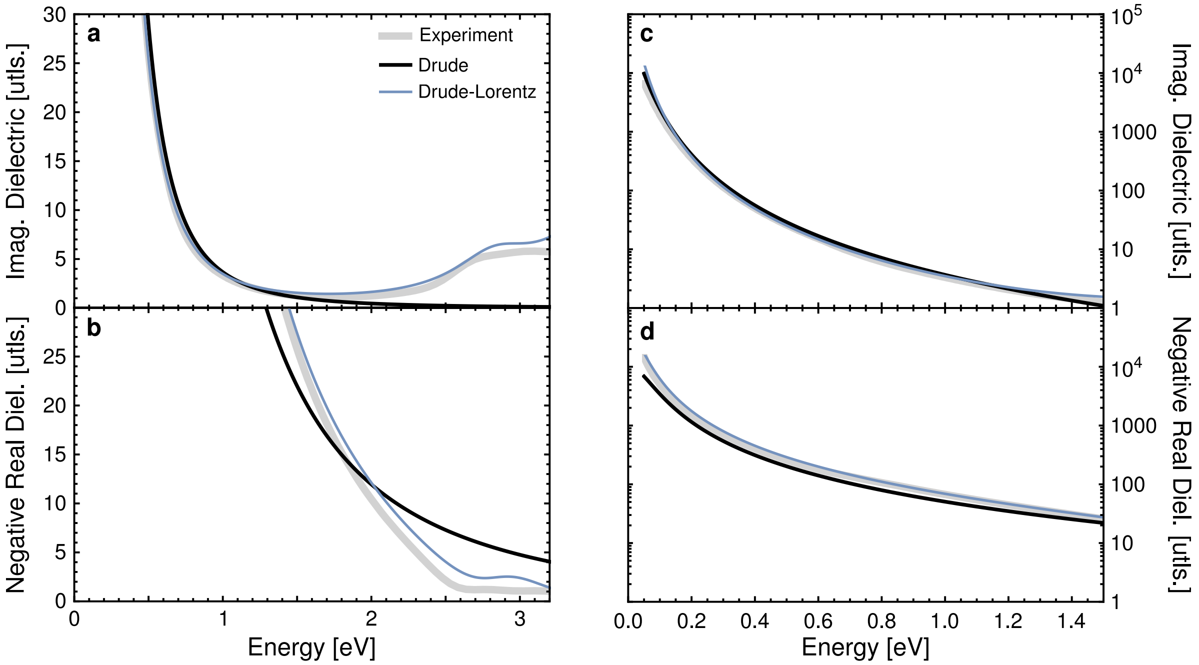

Figure S16: **Au dielectric function fit.** Demonstration of the agreement between Drude and Drude-Lorentz dielectric models (black, blue, red) of $\epsilon_1(\omega)$ and dielectric data from Ref. 4, both in the near-IR to near-UV range (a,b) and in the IR range (c,d).

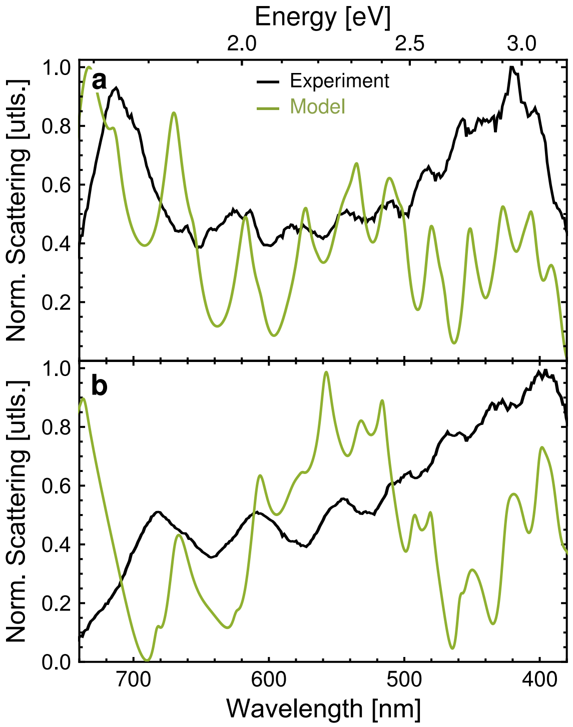

Figure S17: **Additional Mie scattering data.** Comparison of Mie theory (green) to individual bare $LiNbO_3$ scattering data (black) for samples of relatively high (a) and low (b) dielectric function. The experimental and theoretical data are normalized to range from 0 to 1 within this selected frequency window. The theoretical curves are produced using dielectric values of 7.0 + 0.075i (a) and 5.0 + 0.05i (b) such that, as in Figure 3c, the number and spectral position of the sharper visible peaks in either spectrum are in agreement.

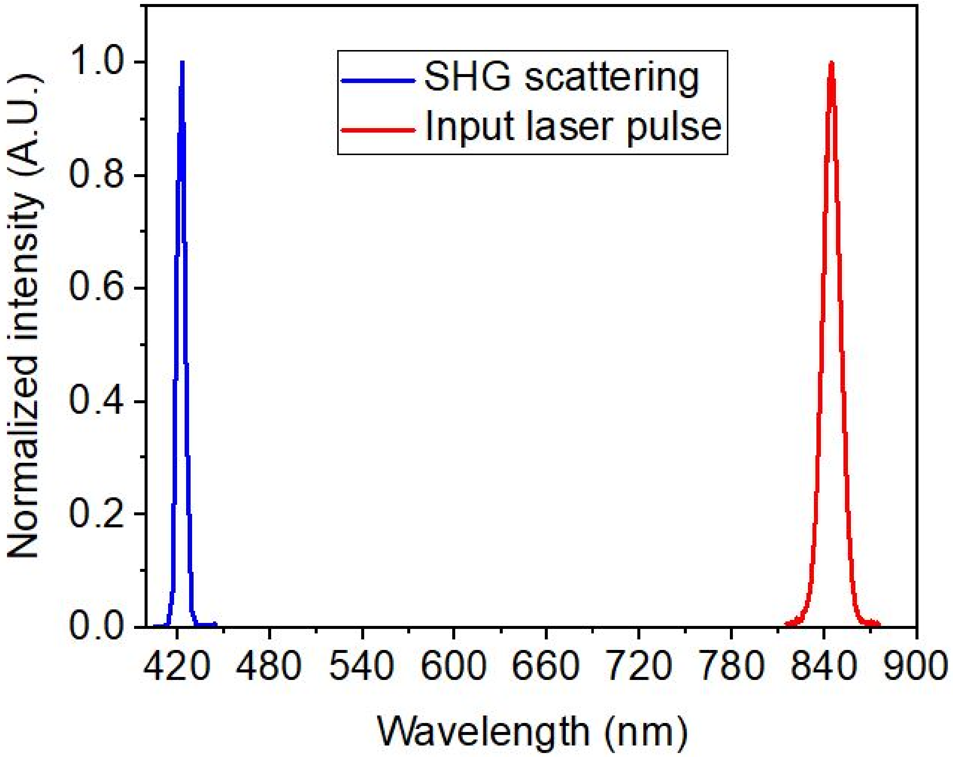

Figure S18: **Input and SHG lineshapes.** Normalized lineshapes of the input laser (red) and SHG (blue) power spectra.

Parameter	Value
$\hbar\omega_1$ (bare)	2.49 eV
$\hbar\omega_1$ ($LiNbO_3$-adjacent)	2.26 eV
$\hbar\omega_{L_1}$	3.07 eV
$\hbar\omega_{L_2}$	6.78 eV
$\hbar\gamma_1$	450 meV
$\hbar\gamma_{L_1}$	688 meV
$\hbar\gamma_{L_2}$	529 meV
ψ_1	-433 mrad
ψ_{L_1}	-260 mrad
ψ_{L_2}	26.5 mrad
μ_1	1.20×10^{-30} g
μ_{L_1}	1.66×10^{-30} g
μ_{L_2}	2.04×10^{-32} g

Table S1: **Au NP oscillator parameters.** Table of oscillator parameters for the model of Au used in this work.

Mode indices	$\hbar\omega_{\boldsymbol{\beta}}$ [eV]	$\hbar\gamma_{\boldsymbol{\beta}}$ [eV]	$\mu_{\boldsymbol{\beta}} \times 10^{38}$ [g]	$\psi_{\boldsymbol{\beta}}$ [rad]
$T = E$, $\ell = 6$	2.24	0.137	1.56	-0.139
$\ell = 7$	2.46	0.0898	0.824	-0.0539
$\ell = 9$	2.28	0.0140	1.70	3.14
$\ell = 10$	2.47	0.0149	1.06	3.14
$T = M$, $\ell = 7$	2.34	0.0394	1.39	-0.127
$\ell = 10$	2.31	0.0144	1.35	3.14
$\ell = 11$	2.50	0.0155	0.835	3.14

Table S2: **$LiNbO_3$ microsphere oscillator parameters.** Oscillator model parameters of the modeled Mie resonances of the $LiNbO_3$ microsphere.

Sample Number	Radius [nm]	Real Dielectric Constant
1	350	6.3
2	350	6.3
3	400	6.0
4	550	5.7
5	600	5.8
6	600	7.0
7	600	5.0
8	1000	6.3
9	1000	5.5

Table S3: **Estimated $LiNbO_3$ microsphere parameters.** Estimates of the dielectric constant of $LiNbO_3$ extracted from Mie theory reproductions of the scattering spectra of nine bare mesoporous $LiNbO_3$ microspheres in the spectra region 1.5–3.5 eV. The radii of the particles were measured by microscopy techniques with a precision of ±50 nm. The real parts of the dielectric functions are estimated to the nearest 0.1.

Parameter	Value
$\hbar\omega_{p1}$	8.94 eV
$\hbar\omega_{p2}$	2.58 eV
$\hbar\omega_{p3}$	6.31 eV
$\hbar\Gamma_1$	42.8 meV
$\hbar\Gamma_2$	660 meV
$\hbar\Gamma_3$	964 meV
$\hbar\Lambda_2$	2.84 eV
$\hbar\Lambda_3$	3.71 eV

Table S4: **Au Drude-Lorentz model parameters.** Table of Drude-Lorentz dielectric function values for the model of Au used in this work. See Figure S16 for further details.

References

[1] Edward D. Palik and Gorachand Ghosh, editors. *Handbook of Optical Constants of Solids*. Academic Press, San Diego, 1998.

[2] Philippe Lalanne, Wei Yan, Kevin Vynck, Christophe Sauvan, and Jean-Paul Hugonin. Light interaction with photonic and plasmonic resonances. *Laser Photonics Rev.*, 12(5): 1700113, 2018.

[3] Agust Olafsson, Jacob A. Busche, Jose J. Araujo, Arpan Maiti, Juan Carlos Idrobo, Daniel R. Gamelin, David J. Masiello, and Jon P. Camden. Electron beam infrared nano-ellipsometry of individual indium tin oxide nanocrystals. *Nano Lett.*, 20(11):7987–7994, 2020.

[4] Robert L. Olmon, Brian Slovick, Timothy W. Johnson, David Shelton, Sang-Hyun Oh, Glenn D. Boreman, and Markus B. Raschke. Optical dielectric function of gold. *Phys. Rev. B*, 86(23):235147, 2012.

[5] G. Pellegrini, G. Mattei, V. Bello, and P. Mazzoldi. Interacting metal nanoparticles: Optical properties from nanoparticle dimers to core-satellite systems. *Mater. Sci. Eng. C*, 27(5-8):1347–1350, 2007.

[6] Ulrich Hohenester and Andreas Trügler. MNPBEM – a Matlab toolbox for the simulation of plasmonic nanoparticles. *Comput. Phys. Commun.*, 183(2):370–381, 2012.

[7] Álvaro González. Measurement of areas on a sphere using fibonacci and latitude–longitude lattices. *Math. Geosci.*, 42(1):49–64, 2010.

[8] Arthur Riefer, Simone Sanna, Alexander V. Gavrilenko, and Wolf Gero Schmidt. Linear and nonlinear optical response of $LiNbO_3$ calculated from first principles. *IEEE Trans. Ultrason. Ferroelectr. Freq. Control*, 59(9):1929–1933, 2012.

[9] Wolfram Research, Inc. Mathematica, version 13.0.0. URL `https://www.wolfram.com/mathematica`. Champaign, IL, 2021.

[10] Romolo Savo, Andrea Morandi, Jolanda S. Müller, Fabian Kaufmann, Flavia Timpu, Marc Reig Escalé, Michele Zanini, Lucio Isa, and Rachel Grange. Broadband Mie driven random quasi-phase-matching. *Nat. Photonics*, 14(12):740–747, 2020.

[11] Y. R. Shen. *The Principles of Nonlinear Optics*. Wiley-Interscience, Hoboken, N.J, wiley classics library edition, 2003.

[12] Charles Cherqui, Guoliang Li, Jacob A. Busche, Steven C. Quillin, Jon P. Camden, and David J. Masiello. Multipolar nanocube plasmon mode-mixing in finite substrates. *J. Phys. Chem. Lett.*, 9(3):504–512, 2018.

[13] Chen-To Tai. *Dyadic Green Functions in Electromagnetic Theory*. IEEE Press series on electromagnetic waves. IEEE Press, Piscataway, NJ, 2nd ed. edition, 1994.

[14] Charles Cherqui, Niket Thakkar, Guoliang Li, Jon P. Camden, and David J. Masiello. Characterizing localized surface plasmons using electron energy-loss spectroscopy. *Annu. Rev. Phys. Chem.*, 67(1):331–357, 2016.

[15] Craig F. Bohren and Donald R. Huffman. *Absorption and Scattering of Light by Small Particles*. Wiley-VCH, Weinheim, 2004.

[16] Luis V. Rodríguez-de Marcos, Juan I. Larruquert, José A. Méndez, and José A. Aznárez. Self-consistent optical constants of SiO_2 and Ta_2O_5 films. *Opt. Mater. Express*, 6(11): 3622–3637, 2016.
